# Antibiotic synergy against *Staphylococcus aureus*: a systematic review and meta-analysis

**DOI:** 10.1128/aac.01199-24

**Published:** 2025-06-17

**Authors:** Madeline Mellett, Alexander Lawandi, Chelsea Caya, Todd C. Lee, Ahmed Babiker, Jesse Papenburg, Cedric P. Yansouni, Matthew P. Cheng

**Affiliations:** 1 Department of Microbiology and Immunology, McGill University 5620, Montreal, Québec, Canada; 2 Department of Critical Care Medicine, McGill University Health Centre 24481, Montreal, Québec, USA; 3 Division of Infectious Diseases, Department of Medicine, McGill University Health Centre 54473, Montreal, Québec, Canada; 4 Division of Medical Microbiology, Department of Laboratory Medicine, McGill University Health Centre, Montreal, Québec, Canada; 5 Research Institute of the McGill University Health Centre 507266, Montreal, Québec, Canada; 6 Division of Infectious Diseases, Department of Medicine, Emory School of Medicine 12239, Atlanta, Georgia, USA; 7 Department of Pathology and Laboratory Medicine, Emory University School of Medicine 12239, Atlanta, Georgia, USA; 8 Division of Pediatric Infectious Diseases, Department of Pediatrics, McGill University Health Centre 54473, Montreal, Québec, Canada; 9 J.D. MacLean Centre for Tropical Diseases, McGill University 5620, Montreal, Québec, Canada; University of California San Francisco, San Francisco, California, USA

**Keywords:** *Staphylococcus aureus*, synergy, antimicrobial combinations

## Abstract

Antimicrobial combinations have been extensively evaluated *in vitro* to identify synergistic combinations for clinical use. Despite the available literature, no studies comprehensively summarize the findings for antimicrobial combinations against *Staphylococcus aureus*. We performed a systematic review to identify synergistic combinations that may be beneficial for clinical use against *S. aureus*. The PubMed, Cochrane, and Web of Science databases were queried from inception to February 2024 for studies of *in vitro* assays evaluating two antimicrobials in combination against isolates of *S. aureus*. Studies were included if they used common methods to determine synergy including time-kill assays, checkerboard assays, or the combined gradient diffusion method. The proportion of isolates for which synergy was identified was compared for different antimicrobial combinations. Two hundred sixty-five studies were included for analysis. One hundred forty-two studies evaluated synergy against methicillin-resistant *S. aureus* (MRSA), 31 against methicillin-susceptible *S. aureus* (MSSA), and 92 assessed synergy against both MRSA and MSSA, or did not define the methicillin susceptibility profile of the isolates studied. Time-kill assays (*n* = 176) and checkerboard assays (*n* = 158) were the most frequently used methods, with few studies evaluating synergy using the combined gradient diffusion method (*n* = 13). The proportion of synergy varied based on the antimicrobial combination and isolate being evaluated. Antimicrobial synergy has been extensively studied for *S. aureus*, with combinations of glycopeptides and cephalosporins being studied most frequently. Future evaluations of synergy for *S. aureus* should focus on antimicrobial combinations with strong rationales and robust potential for clinical use.

## INTRODUCTION

Antimicrobial combinations for the treatment of bacterial infections have been evaluated due to theoretical advantages (1–3). Clinically, the treatment of polymicrobial infections may benefit from multiple antibiotics empirically as they widen the spectrum of activity compared to monotherapy (1–4). Combination therapy may also be useful for overcoming the Eagle effect, which describes how bacteria survive while in their stationary phase growth, despite being exposed to antimicrobial therapy. This phenomenon may be overcome with the addition of antibiotics with different mechanisms of action (5). Furthermore, combinations could potentially suppress the emergence of antimicrobial resistance, repurpose antibiotics no longer used therapeutically, and may improve clinical efficacy through synergistic activity (1–3, 6, 7).

An antimicrobial combination is said to be synergistic when the combination results in greater growth inhibition than the sum of the individual activities of the compounds (1–3, 6). In the laboratory, the assessment of antimicrobial synergy can be performed using several methods including the time-kill assay (TKA), the checkerboard assay, and combined gradient strip diffusion assays using the epsilometer (Etest) method. The TKA assesses the killing activity of antimicrobials alone and in combination at several time points over a 24 hour incubation period. Synergy is commonly defined as a ≥2log_10_ reduction in colony forming units (cfus)/mL at 24 hours for the combination in comparison to the single most active drug (8).

In contrast, checkerboard assays evaluate multiple concentrations of the combination of antibiotics at a fixed time point, and synergy is defined according to the fractional inhibitory concentration index (FICI). The FICI is a mathematical equation used to define the effect of antibiotics in combination, whereby synergy is defined as at least a fourfold decrease in the minimum inhibitory concentration (MIC) for each antibiotic when used together (9). The gradient strip diffusion method can also be used for synergy testing. This method has been modified from the standard operating procedure of the strip use to evaluate changes in MIC that reflect synergy or antagonism. Several methods for assessing synergy exist, including the direct overlay method, MIC:MIC method, agar diffusion method, and the cross method of gradient diffusion strips on inoculated agars, with recording of changes in the individual MICs on the different strips (6, 10, 11).

Despite this variety of methods for synergy determination, few studies have comprehensively evaluated these methods to identify synergistic antibiotic combinations against methicillin-susceptible *Staphylococcus aureus* (MSSA) and methicillin-resistant *S. aureus* (MRSA). These pathogens are of particular interest as they can both cause invasive infections with high mortality rates (12–19). While synergy has been described as a rationale for studying antibiotic combinations in clinical practice, including in randomized controlled trials, there is a paucity of data regarding effective combinations for both organisms (20–22). Given the impact clinical trials can have on local prescribing behavior, as well as the potential harms of using multiple antibiotics including increased risk of adverse events, it is imperative to establish a strong rationale for these combinations prior to study in a clinical setting or adoption into clinical practice (22–25).

We conducted a systematic review of *in vitro* synergy studies targeting MSSA and MRSA. Our objectives were to identify all antimicrobial combinations that have been evaluated through frequently used methods of synergy testing and establish antimicrobial combinations that result in a high proportion of synergy to be explored further in *in vivo* and clinical settings.

## MATERIALS AND METHODS

### Information sources and search strategy

We performed a systematic review of studies evaluating *in vitro* antimicrobial synergy to identify all combinations of antibiotics tested for synergy against *S. aureus* and *S. pyogenes*. Due to limited studies evaluating *S. pyogenes* being identified, studies evaluating synergy against *S. pyogenes* were removed from the analysis. This study was not registered in Prospero.

Studies were identified through searching PubMed on 28 September 2021 and Web of Science and Cochrane databases on 25 May 2022. All databases were re-searched on 15 February 2024. After deduplication, articles were rescreened to identify recently published articles eligible for inclusion.

Our search strategy included *S. pyogenes*, *S. aureus*, and commonly used antibiotics for the treatment of these pathogens in a clinical setting. The full search strategy is available in the Supplementary methods and materials and consists of the following keywords and their synonyms: combination, synergy, time-kill assay, *in vitro*, antibiotic, *S. pyogenes*, and *S. aureus*, and the following selected antibiotics are chosen to reflect frequently used antibiotics: penicillin, vancomycin, linezolid, quinupristin, dalfopristin, daptomycin, nafcillin, oxacillin, cefazolin, clindamycin, gatifloxacin, tedizolid, and ceftriaxone.

### Inclusion criteria

English language studies were eligible for inclusion if they used standardized criteria for the TKA, checkerboard assays, or combined gradient strip diffusion assays using the cross or MIC:MIC methods to assess for synergy against *S. aureus* (including MRSA and MSSA). Studies using alternative methods for synergy testing, including qualitative methods such as modified disk-diffusion assays and pharmacodynamic and pharmacokinetic models, were excluded, as well as studies using *in vivo* or biofilm models. Studies evaluating *S. aureus* isolates from non-human sources were excluded. We also excluded combinations tested against vancomycin-resistant *S. aureus* isolates, because those with complete resistance (MIC ≥ 16 mg/L) are rare compared to MSSA or MRSA (26).

### Outcome measurements

The outcome of interest was the number of isolates where synergy was identified for each combination tested. Synergy was evaluated according to existing standardized criteria for each assay. Synergy for the TKA was defined as a ≥2log_10_ decrease in cfus after 24 hours for the combination compared to the single most active agent alone (8). Using the checkerboard assay and the combined gradient strip diffusion, the FICI was used to evaluate synergy. FICI was calculated according to the following formula: ΣFICI = FIC_A_ + FI_CB_, where FIC_A_ = MIC_(A in the presence of B)_/MIC_(A alone)_, FIC_B_ = MIC_(B in the presence of A)_/MIC_(B alone)_ (9). An FICI ≤ 0.5 is consistent with synergism, 0.5 < FIC ≤ 4 with indifference, and an FIC > 4 with antagonism (9).

### Data collection processes

Potentially relevant studies were first screened by title and abstract using Rayyan software. They were then assessed for full-text eligibility by one reviewer (M.M.). The reviewer independently extracted data from each study. Study publication date, author names, method of synergy evaluation (checkerboard, combined gradient strip diffusion, or TKA), definition of synergy used, antimicrobial combinations studied, isolate type (MSSA or MRSA), the number of isolates evaluated, and the number of isolates against which synergy was identified for each antibiotic combination were recorded. Studies using rifampicin were recorded as using rifampin. When studies reported the findings of synergy for both MRSA and MSSA isolates together without distinguishing against which isolates synergy was identified, the results were classified and grouped as *S. aureus*. Studies that did not describe the methicillin-resistance profile of the *S. aureus* isolates were also categorized as *S. aureus*. When more than one method was used to evaluate synergy, results from the checkerboard assay were reported, when available. Studies using both the checkerboard and the TKA frequently only assessed a subset of isolates using the TKA (27–49). Therefore, we prioritized the checkerboard results. When FICI was presented as FICI_min_ and FICI_max_, FICI_min_ was used to report synergy. In instances when the results of the checkerboard assay were uninterpretable, the results of the other assay were recorded. If the checkerboard assay was not performed, the results of the TKA were reported at the concentration resulting in the greatest synergy for each isolate. If both the TKA and the combined gradient strip diffusion were performed, the results of the TKA were recorded. When no definition of synergy was provided, or if a non-standard synergy definition was used, we interpreted synergy according to the previously described criteria when possible. Otherwise, studies were excluded from the meta-analysis.

### Data synthesis and meta-analysis

Analysis of the proportion of synergy for each combination of antibiotic classes was determined by separating each study according to both the class of antibiotic combinations tested and the isolate studied. β-Lactam antibiotics were separated into either carbapenems, cephalosporins, mono-bactams, or other β-lactams, and vancomycin was defined as a glycopeptide. A detailed list of how antibiotics were classified can be found in Table S1.

The number and proportion of isolates against which synergy was identified were summed for each category and compared to the number of isolates tested to determine the proportion of synergy for each combination. A random effects meta-analysis of proportions was also performed for classes of antibiotics frequently tested using the MetaXL software package (version 5.3) for prevalence using pooled effects analysis. *I*^2^ heterogeneity and 95% confidence interval (CI) were also calculated using MetaXL. Highly synergistic combinations were identified as combinations tested in at least three separate studies that resulted in the greatest proportion of synergy relative to all combinations tested in at least three separate studies identified in this review.

Percent agreement and Cohen’s kappa were determined as previously described (50). Studies using more than one method were identified, and those testing the same isolate using more than one method were included in the analysis. Agreement for synergy or no synergy was determined for each antibiotic combination tested against each isolate in the studies identified.

## RESULTS

### Study selection and study characteristics

**Fig 1:**
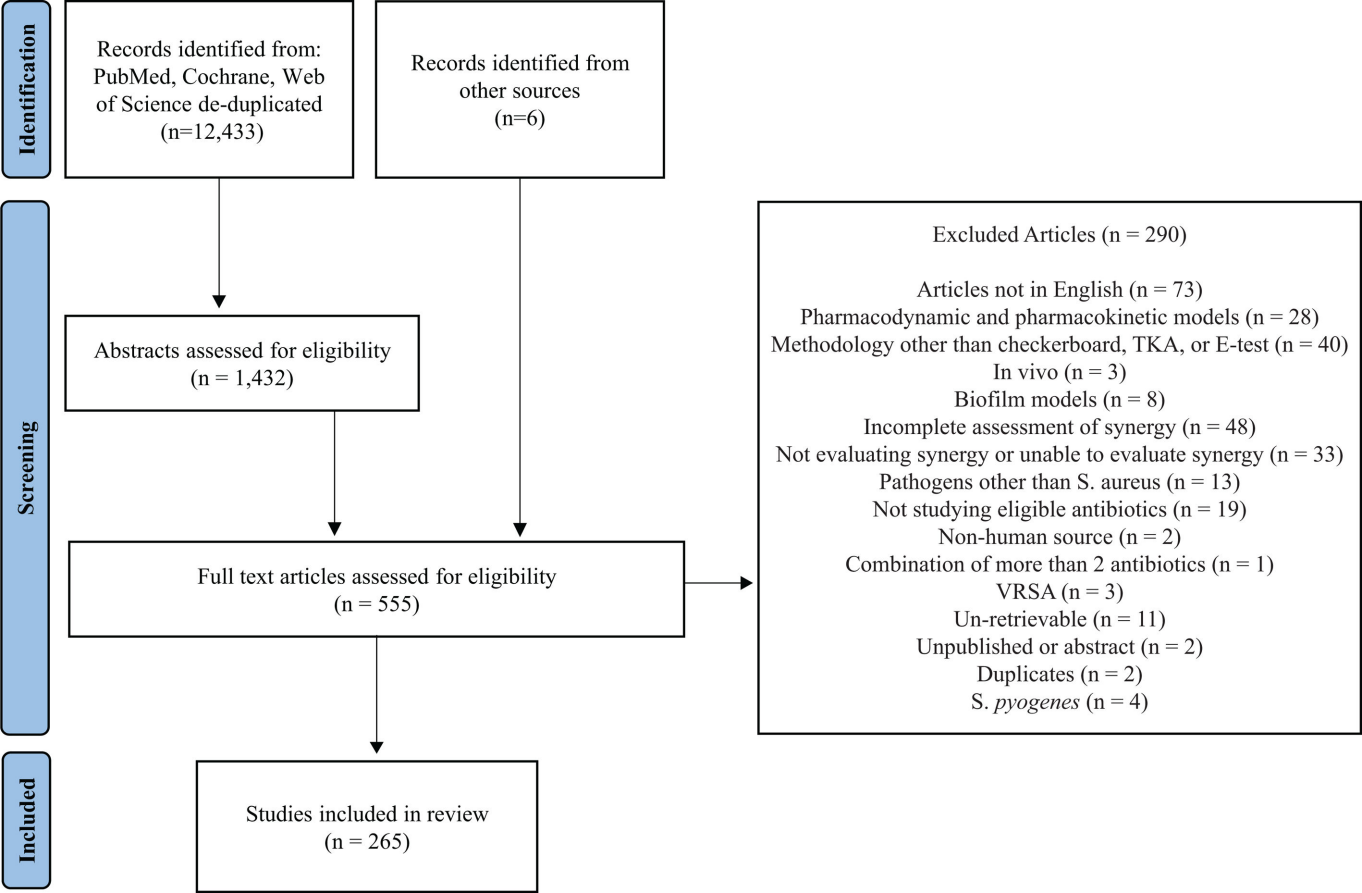
Flow diagram of included studies evaluating antibiotic synergy for *S. aureus*.

Our searches identified 12,433 articles. Following both title and abstract screening, 555 articles were assessed for full-text eligibility (Fig. 1). Full-text assessment identified 314 studies using appropriate *in vitro* synergy tools for synergy assessment, of which 49 were subsequently excluded due to incomplete or uninterpretable synergy data.

Thirty-three studies were excluded because there was no description of their approach for evaluating synergy, an alternative definition of synergy was used, or synergy was not determined despite using an eligible method for determining synergy. Sixteen other studies were excluded as they did not provide findings for individual isolates: 13 presented synergy results for all evaluated isolates together as either a mean log change of cfu for TKA or as a mean FICI or range of FICIs for the checkerboard assay, 1 combined the results of synergistic isolates with partially synergistic isolates, and 2 combined their findings of synergy with isolates other than *S. aureus*. Following the exclusion of these studies, 265 studies were included in our systematic review.

Characteristics of the included studies are summarized in Table S2. One hundred forty-two studies assessed synergy against MRSA isolates (142/265, 53.5%), 31 against MSSA isolates (31/265, 11.7%), and an additional 55 evaluated both MRSA and MSSA (55/265, 20.7%). Thirty-seven studies assessed synergy in *S. aureus* isolates where synergy outcomes were not separated for MRSA and MSSA isolates, or the methicillin-resistance profile of isolates was not described (37/265, 14.0%).

### Checkerboard, TKA, and combined gradient diffusion methods of synergy determination

The TKA and checkerboard methods were the most frequent methods of synergy evaluation identified in our systematic review. One hundred fifty-eight studies (158/265, 59.6%) used the checkerboard assay and 176 (176/265, 66.4%) used the TKA. The combined gradient strip diffusion method was used for synergy determination in 12 studies (12/265, 4.6%). Eighty-one studies (81/265, 30.6%) used more than one method to assess for synergy, 76 of which used the checkerboard and TKA (76/265, 28.7%), 2 used the TKA and combined gradient strip diffusion method (2/265, 0.8%), 2 employed both the checkerboard and combined gradient diffusion method (2/265, 0.8%), and 1 used all three methods (1/265, 0.4%).

Different studies using the same methodology did not consistently present their data using established criteria for synergy. To fully capture discrepancies related to synergy criteria, we evaluated the 314 studies identified as using *in vitro* synergy methods during full-text screening and classified each study as meeting or not meeting standard criteria. Synergy was least frequently described according to standard criteria for the TKA. Details for all methods can be found in the supplementary materials (see Table S3).

### *In vitro* antimicrobial synergy

Synergy was evaluated in 11,738 isolates, where MRSA isolates were studied most frequently (8,760/11,738; 74.6%), followed by MSSA isolates (1,576/11,738; 13.4%) and *S. aureus* isolates (1,402/11,738; 12.0%). The number of isolates tested by each individual study ranged from 1 to 114 isolates. Synergy results for the 10 frequently tested combinations against all isolates included combinations of glycopeptides with cephalosporins, carbapenems, aminoglycosides, rifamycins, and β-lactams, along with combinations of β-lactams with aminoglycosides and lipopeptides, and cephalosporins with carbapenems, lipopeptides, and fosfomycin. The estimated proportion of synergy for frequently tested combinations is provided in Table 1; Fig. S1 to S3 for each isolate type. Meta-analyses for all combinations tested against each isolate can be found in Fig. S4 to S6.

**TABLE 1:** Number of synergistic isolates for each type of isolate evaluated for the most frequently evaluated antibiotic combinations

Isolate type	Drug combinations^*a*^	Isolates tested and estimated proportion of synergy (proportion, 95% CI)
MRSA	All	4,037/8,760 (0.46, 0.42–0.50)
	Cephalosporins and glycopeptides	700/1,362 (0.56, 0.47–0.65)
	Cefamandole combinations^*b*^	63/102 (0.47, 0.03–0.93)
	Cefazolin combinations	121/205 (0.68, 0.55–0.81)
	Cefozopran combinations	58/98 (0.65, 0.00–1.00)
	Cefepime combinations	71/158 (0.53, 0.31–0.75)
	Cefdinir combinations	0/6 (0.06, 0.00–0.33)
	Cefmetazole combinations	64/98 (0.80, 0.46–1.00)
	Cefotaxime combinations	139/223 (0.67, 0.51–0.82)
	Cefoxitin combinations	34/58 (0.66, 0.41–0.89)
	Cefpirome combinations	48/78 (0.60, 0.43–0.76)
	Ceftaroline combinations	29/204 (0.28, 0.08–0.52)
	Ceftobiprole combinations	2/30 (0.29, 0.00–0.81)
	Ceftriaxone combinations	13/17 (0.70, 0.14–1.00)
	Cephalothin combinations	28/40 (0.61, 0.00–1.00)
	Carbapenems and glycopeptides	505/643 (0.78, 0.69–0.85)
	Doripenem combinations	46/54 (0.85, 0.69–0.97)
	Imipenem combinations	254/330 (0.74, 0.57–0.89)
	Meropenem combinations	120/158 (0.78, 0.66–0.88)
	Panipenem combinations	85/101 (0.85, 0.72–0.95)
	Carbapenems and cephalosporins	334/476 (0.72, 0.56–0.85)
	Imipenem combinations	180/233 (0.81, 0.59–0.98)
	Meropenem combinations	152/240 (0.56, 0.37–0.74)
	Cephalosporins and lipopeptides	145/330 (0.58, 0.40–0.76)
	Cefazolin combinations	19/19 (0.98, 0.89–1.00)
	Cefepime combinations	13/35 (0.39, 0.00–1.00)
	Cefotaxime combinations	17/17 (0.98, 0.88–1.00)
	Ceftaroline combinations	45/165 (0.39, 0.19–0.60)
	Ceftobiprole combinations	6/17 (0.48, 0.00–1.00)
	Ceftriaxone combinations	26/54 (0.41, 0.00–1.00)
	Aminoglycosides and β-lactams^*c*^	17/23 (0.72, 0.52–0.88)
	Ampicillin-sulbactam combinations	7/10 (0.54, 0.00–1.00)
	β-Lactams and lipopeptides	155/325 (0.63, 0.42–0.82)
	Ampicillin combinations	40/67 (0.49, 0.05–0.94)
	Cloxacillin combinations	6/6 (0.94, 0.69–1.00)
	Oxacillin combinations	36/149 (0.49, 0.04–0.95)
	Nafcillin combinations	16/25 (0.76, 0.38–1.00)
	Piperacillin and piperacillin-tazobactam combinations	31/46 (0.64, 0.31–0.92)
	Aminoglycosides and glycopeptides	90/280 (0.38, 0.21–0.56)
	Amikacin combinations	3/36 (0.10, 0.00–0.44)
	Arbekacin combinations	25/29 (0.85, 0.70–0.96)
	Gentamicin combinations	58/137 (0.46, 0.25–0.67)
	Tobramycin combinations	4/46 (0.09, 0.02–0.20)
	Glycopeptides and rifamycins	17/220 (0.09, 0.02–0.18)
	Teicoplanin combinations	1/7 (0.25, 0.00–0.86)
	Vancomycin combinations	5/191 (0.03, 0.01–0.07)
	β-Lactams and glycopeptides	139/246 (0.58, 0.44–0.72)
	Ampicillin-sulbactam combinations	6/9 (0.08, 0.00–0.45)
	Nafcillin combinations	12/37 (0.81, 0.44–1.00)
	Oxacillin combinations	107/159 (0.60, 0.44–0.75)
	Piperacillin, piperacillin-tazobactam combinations	12/37 (0.20, 0.00–0.65)
	Cephalosporins and fosfomycin	243/287 (0.87, 0.74–0.96)
	Cefazolin combinations	79/83 (0.95, 0.88–0.99)
	Cefmetazole combinations	55/84 (0.66, 0.55–0.75)
MSSA	All	417/1,576 (0.33, 0.28–0.38)
	Cephalosporins and glycopeptides	11/37 (0.43, 0.10–0.79)
	Cefdinir combinations	0/6 (0.07, 0.00–0.33)
	Ceftaroline combinations	6/6 (0.54, 0.00–1.00)
	Cefotaxime combinations	2/11 (0.85, 0.28–1.00)
	Carbapenems and glycopeptides	22/24 (0.90, 0.73–1.00)
	Imipenem combinations	11/12 (0.91, 0.65–1.00)
	Meropenem combinations	11/12 (0.87, 0.54–1.00)
	Carbapenems and cephalosporins	32/82 (0.45, 0.22–0.70)
	Ertapenem combinations	6/10 (0.71, 0.19–1.00)
	Imipenem combinations	19/47 (0.40, 0.06–0.78)
	Cephalosporins and lipopeptides	9/69 (0.16, 0.02–0.39)
	Ceftaroline combinations	5/22 (0.27, 0.00–0.74)
	Aminoglycosides and β-lactams	54/225 (0.30, 0.21–0.41)
	Amikacin combinations	2/2 (0.86, 0.28–1.00)
	Gentamicin combinations	13/44 (0.50, 0.18–0.82)
	Netilmicin combinations	15/71 (0.23, 0.10–0.37)
	Sisomicin combinations	12/71 (0.17, 0.09–0.27)
	Tobramycin combinations	12/37 (0.55, 0.06–1.00)
	β-Lactams and lipopeptides	9/48 (0.26, 0.00–0.66)
	Ampicillin combinations	0/21 (0.02, 0.00–0.09)
	Oxacillin combinations	5/21 (0.21, 0.05–0.39)
	Aminoglycosides and glycopeptides	19/35 (0.53, 0.37–0.69)
	Gentamicin combinations	14/24 (0.57, 0.35–0.77)
	Glycopeptides and rifamycins	0/69 (0.02, 0.00–0.06)
	Vancomycin combinations	0/68 (0.02, 0.00–0.06)
	β-Lactams and glycopeptides	7/46 (0.25, 0.00–0.65)
	Oxacillin combinations	2/40 (0.11, 0.00–0.32)
	Cephalosporins and fosfomycin	7/12 (0.52, 0.00–1.00)
	Cefazolin combinations	7/9 (0.71, 0.00–1.00)
*S. aureus^d^*	All	304/1,402 (0.16, 0.12–0.21)
	Cephalosporins and glycopeptides	3/20 (0.16, 0.03–0.36)
	Carbapenems and glycopeptides	10/30 (0.37, 0.19–0.56)
	Imipenem combinations	9/21 (0.44, 0.23–0.66)
	Aminoglycosides and β-lactams	32/150 (0.24, 0.08–0.44)
	Gentamicin combinations	9/44 (0.24, 0.01–0.55)
	Netilmicin combinations	15/40 (0.00–1.00)
	Aminoglycosides and glycopeptides	28/41 (0.68, 0.53–0.81)
	Gentamicin combinations	27/40 (0.67, 0.52–0.81)
	Glycopeptides and rifamycins	0/4 (0.09, 0.00–0.45)

^*a*^ Only a subset of combinations are shown. To see forest plots for the top 10 frequently tested combinations refer to Fig. S1 to S3. To see all combinations tested, refer to Fig. S4 to S6.

^*b*^ Subgroup analysis was conducted when the same antibiotic was tested 2 or more times.

^*c*^ The β-lactam group includes non-carbapenem, non-cephalosporin β-lactams. Refer to supplementary table 1 for additional details on how antibiotics were categorized.

^*d*^ Studies evaluating MRSA and MSSA where results of synergy testing for both isolates were presented together, or the methicillin resistance profile was not provided were classified as *S. aureus*.

**Fig 2:**
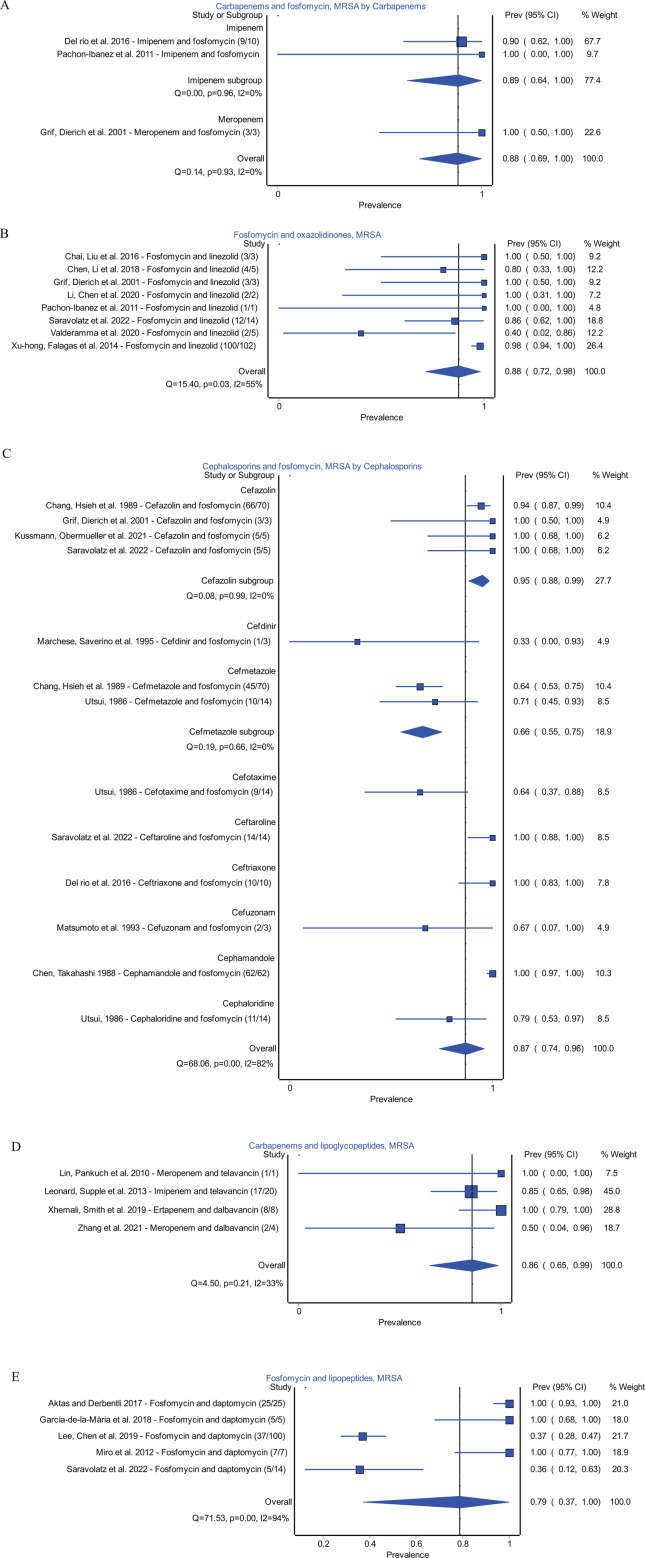
Top five synergistic combinations for MRSA. (**A**) Fosfomycin and carbapenems. (**B**) Fosfomycin and oxazolidinones. (**C**) Cephalosporins and fosfomycin. (**D**) Carbapenems and lipoglycopeptides. (**E**) Fosfomycin and lipopeptides.

In order to determine combinations associated with a high proportion of synergy against MRSA and MSSA isolates, we assessed the proportion of synergy for combinations studied in at least three independent studies. The five combinations with the greatest proportion of synergy for MRSA or MSSA were identified. These combinations for MRSA isolates included carbapenems and fosfomycin (synergistic isolates/isolates tested: 13/14; *n* = 3 studies) (41, 51, 52), fosfomycin and oxazolidinone combinations (127/135; *n* = 8 studies) (36, 41, 52–57), cephalosporins and fosfomycin combinations (233/277; *n* = 9 studies) (41, 51, 57–63), carbapenems and lipoglycopeptides (28/33; *n* = 4 studies) (64–67), and combinations of fosfomycin and lipopeptides (78/151, *n* = 5 studies) (57, 68–71). Forest plots for these combinations and estimated proportions can be found in Fig. 2.

**Fig 3:**
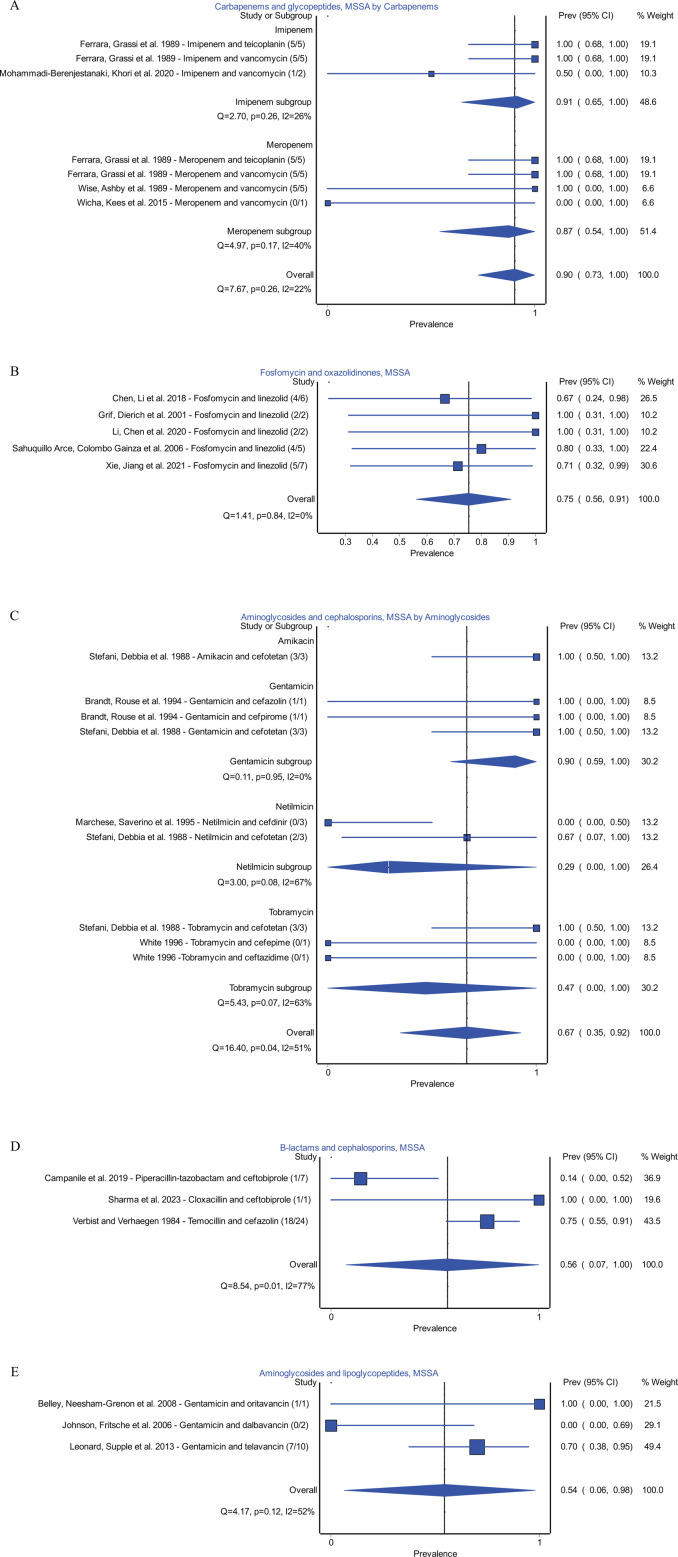
Top five synergistic combinations for MSSA. (**A**) Carbapenems and glycopeptides. (**B**) Fosfomycin and oxazolidinones. (**C**) Aminoglycosides and cephalosporins. (**D**) β-Lactams and cephalosporins. (**E**) Aminoglycosides and lipoglycopeptides.

Combinations with a high proportion of synergy for MSSA isolates included combinations of carbapenems and glycopeptides (22/24, *n* = 4 studies) (35, 72–74), fosfomycin and oxazolidinone combinations (17/22; *n* = 5 studies) (36, 41, 44, 53, 75), aminoglycosides and cephalosporins (13/19, *n* = 4 studies) (62, 76–78), β-lactams and cephalosporins (20/32; *n* = 3 studies) (79–81), and aminoglycosides with lipoglycopeptides (8/13, *n* = 3 studies) (65, 82, 83). Forest plots can be found in Fig. 3.

A subgroup analysis was performed for each frequently tested combination and each synergistic combination when possible. Notable combinations resulting in a higher proportion of synergy compared to other antibiotics of the same class include cephalosporin and fosfomycin combinations using cefazolin in MRSA isolates and aminoglycoside and cephalosporin combinations using gentamicin for MSSA isolates. A detailed subgroup analysis for synergistic and frequently tested combinations can be found in Fig. 1 to 3; Fig. S1 to S3 .

Synergism, therefore, appears to vary based on both the antibiotic combination and isolate being evaluated.

### Method of synergy determination

In order to address the issue of comparability between methods of synergy testing, we compared the results of synergy assessment for isolates treated with the same combinations of antibiotics using more than one method in the same study. Although 81 studies were identified to use more than one method to test synergy, only 43 studies provided synergy results for the same isolate through more than one method, with 40 studies testing checkerboard and TKA methods, 2 studies testing checkerboard and combined gradient diffusion methods, and 1 study testing TKA and combined gradient diffusion methods with the same isolate. Overall percent agreement between the checkerboard and TKA methods was 70.3% with a kappa statistic of 0.41 (95% CI: 0.31–0.50), indicating moderate agreement between the checkerboard and TKA (50).

## DISCUSSION

In this study, we report the first comprehensive systematic review evaluating antimicrobial synergy against each of MRSA and MSSA. We found combinations of cephalosporins and glycopeptides to be most frequently tested and combinations of fosfomycin with oxazolidinones to be synergistic against *S. aureus* isolates, with even greater levels of synergy against MRSA isolates.

Our findings affirm those of several previous literature reviews of antibiotic synergy and build upon them in several ways. We investigated all studied combinations evaluating synergy for MRSA and MSSA, while previous reviews have only focused upon specific antimicrobial combinations. Steenbergen et al. performed a literature review investigating combinations of daptomycin against MRSA using animal models and pharmacodynamic models in addition to *in vitro* TKA and checkerboard assays (84). Similarly, Neu performed two reviews evaluating antimicrobial combinations with quinolones through *in vitro* approaches, whereas Antonello et al. explored synergy for daptomycin or fosfomycin combinations for *S. aureus* among other isolates (85–88). Although these reviews identified similar studies to our own, they are limited in that their focus is on specific combinations. Some of these studies also used different synergy definitions to our approach, with TKA experiments being defined as only >2log_10_ killing without specifying comparison to the most active single antibiotic or the time point for this interpretation, further highlighting challenges with adherence to standard definitions of synergy (85, 86).

Direct comparison between different methods of synergy determination is also required for future testing of *S. aureus* to establish the comparability of methods (89). White et al. evaluated the combinations of ceftazidime and cefepime with ciprofloxacin and tobramycin through the checkerboard, TKA, and combined gradient diffusion methods against isolates of *Pseudomonas aeruginosa*, *Enterobacter cloacae*, *Escherichia coli*, and MSSA isolates (78). Agreement between the checkerboard and time-kill methods ranged from 44% to 88%, with different concentrations used in the TKA (78). Similarly, agreement between the TKA and combined gradient diffusion method ranged from 63% to 75% (78). These findings are consistent with the percent agreement of 70.3% between checkerboard and TKA approaches for *S. aureus* identified in our study. Considering that the *in vitro* methods evaluate different aspects of antibiotic combinations, namely bactericidal activity for the TKA and inhibitory activity for the checkerboard assay, it is not surprising that these assays do not fully correlate (78).

Rather, it may be more pertinent to establish which approach is better associated with *in vivo* synergy and clinical benefit, as the applicability of *in vitro* testing results to clinical outcomes is poorly established (3, 90). Identifying the predictive capacity of *in vitro* testing for outcomes of *in vivo* assessment of combinations is limited by study design. Although many studies have used *in vivo* models to confirm *in vitro* synergy, studies frequently assess *in vivo* efficacy against a subset of isolates for which combinations have already demonstrated synergy (33, 56, 91). This is further challenged by few studies reporting findings of *in vitro* synergy testing alongside patient outcomes for those treated with a combination of antibiotics (92), despite synergy being the rationale for the combination used. Moreover, the use of antimicrobial combinations for *S. aureus* infections has yet to result in improved clinical efficacy. This challenge is highlighted by combination therapy use in severe *S. aureus* bacteremia not translating to improved mortality outcomes for patients (93, 94). However, antimicrobial combinations selected in recent randomized clinical trials are not among those identified as resulting in a high proportion of synergy in this study. Combinations of fosfomycin with cloxacillin for MSSA bacteremia, adjunctive rifampin for MSSA and MRSA bacteremia, and daptomycin with fosfomycin for MRSA bacteremia have recently been explored in randomized clinical trials (24, 95). No significant benefit was seen for either combination over monotherapy, although the trial evaluating the combination of fosfomycin and cloxacillin may have benefited from a later endpoint and increased sample size (24, 95, 96). In this work, the clinically tested combinations of fosfomycin and β-lactams for MSSA isolates or rifampin-based combinations for MSSA and MRSA isolates were not among combinations identified to result in a high estimated proportion of synergy. In contrast, the combination of fosfomycin and daptomycin was among synergistic combinations (Fig. 2E). Although treatment success associated with mortality at 6 weeks was not significantly improved with this combination, microbiological failure associated with persistent and recurrent bacteria was significantly reduced, suggesting the potential microbiological benefits of this synergistic combination (96). Nonetheless, these clinical studies of antimicrobial combinations have not directly compared clinical and microbiological outcomes for patients alongside *in vitro* synergy testing using patient isolates, which would be needed to fully understand the translational potential of synergy testing. This systematic review aims to serve as a comprehensive tool to select established synergistic antibiotic combinations for future study through *in vivo* models and in clinical settings.

Our systematic review has several limitations. While we succeeded in comprehensively summarizing antibiotic combinations that have been tested against *S. aureus*, our findings did not exclude combinations with agents that may no longer be available for clinical use. In addition, to summarize the results of synergy testing for many antimicrobial combinations, we did not account for discrepancies in synergy interpretation at different antimicrobial concentrations, with different bacterial inocula sizes, or different testing conditions. Although this allowed for a broad interpretation of synergy, further comparison focusing on antibiotic combinations at clinically relevant concentrations and inocula size may be beneficial. We also did not assess dynamic *in vitro* models or *in vivo* models of assessing the efficacy of antimicrobial combinations, as this was outside the scope of our review. However, this may have excluded the assessment of clinically relevant antimicrobial combinations from our analysis. Future work to assess these models and determine the correlation to clinical outcomes would be valuable. Lastly, our systematic review is limited in that only studies in English were included, thereby potentially excluding relevant articles from our analysis, and only one reviewer performed the screening and data extraction, increasing the likelihood that relevant articles or data were missed during the completion of the review.

Future evaluation of antimicrobial synergy should focus on antimicrobial combinations in parallel with robust clinical or *in vivo* studies (3). Similarly, efforts targeting synergistic combinations with potential clinical benefits and favorable safety profiles that can be practically implemented in clinical settings should be prioritized, particularly if *in vitro* synergy has been demonstrated at clinically relevant antimicrobial concentrations. We identified that combinations of fosfomycin with linezolid, cefazolin, or carbapenems resulted in high proportions of synergy for MRSA isolates, and combinations of fosfomycin with linezolid resulted in high proportions of synergy for MSSA isolates. Synergy observed with fosfomycin and oxazolidinone combinations is thought to be attributed to the distinct activities of these antibiotics. Fosfomycin inhibits bacterial cell wall synthesis, potentially enhancing linezolid entry into the cell to then inhibit bacterial protein synthesis (53, 54). Comparatively, in combinations of fosfomycin with cephalosporins and carbapenems, it is thought that fosfomycin may alter penicillin-binding protein expression or function, increasing sensitivity to β-lactams (51, 60). The exact mechanism of these synergies remains to be fully established. Further study into *in vivo* and clinical use of these combinations may be warranted as these antibiotics can be used clinically for *S. aureus* infections including bacteremia.

Future evaluation of synergy may also benefit from increased evaluation of the combined gradient strip diffusion method given its ease of use. This method was infrequently assessed to evaluate synergy, with only 13 studies employing this method. Yet, there was a strong agreement between the combined gradient strip diffusion and checkerboard approaches when comparing results for the same isolates tested in the same study using both methods (78, 97). Given that this approach is advantageous for large-scale synergy studies due to the simplicity of performing the combined gradient strip diffusion and interpreting the results, a high agreement between these methods suggests that combined gradient strip diffusion may be useful in future synergy assessment and parallel use in clinical settings (98).

In summary, this meta-analysis presents a comprehensive review of previously performed synergy assessments that serves as a valuable tool for directing antibiotic selections in future *in vitro* synergy testing and serves as a guide for selecting synergistic antimicrobial combinations to explore within a clinical setting.

## SUPPLEMENTAL MATERIAL

Supplemental figures aac.01199-24-s0001.docx Fig. S1 to S6.

Supplementary methods and materials aac.01199-24-s0002.docx Tables S1 to S3.

ASM does not own the copyrights to Supplemental Material that may be linked to, or accessed through, an article. The authors have granted ASM a non-exclusive, world-wide license to publish the Supplemental Material files. Please contact the corresponding author directly for reuse.
