# Supplementary material for: Antibiotic synergy against Staphylococcus aureus: a systematic review and meta-analysis
Source: Antimicrob Agents Chemother. 2025 Jun 17;69(8):e01199-24. doi: 10.1128/aac.01199-24 (PMC12326989; doi:10.1128/aac.01199-24)
Supplement: Supplemental figures — Fig. S1 to S6. [file aac.01199-24-s0001.docx]

**Antibiotic Synergy against Staphylococcus aureus: A Systematic Review**

**Supplementary Figures**

A


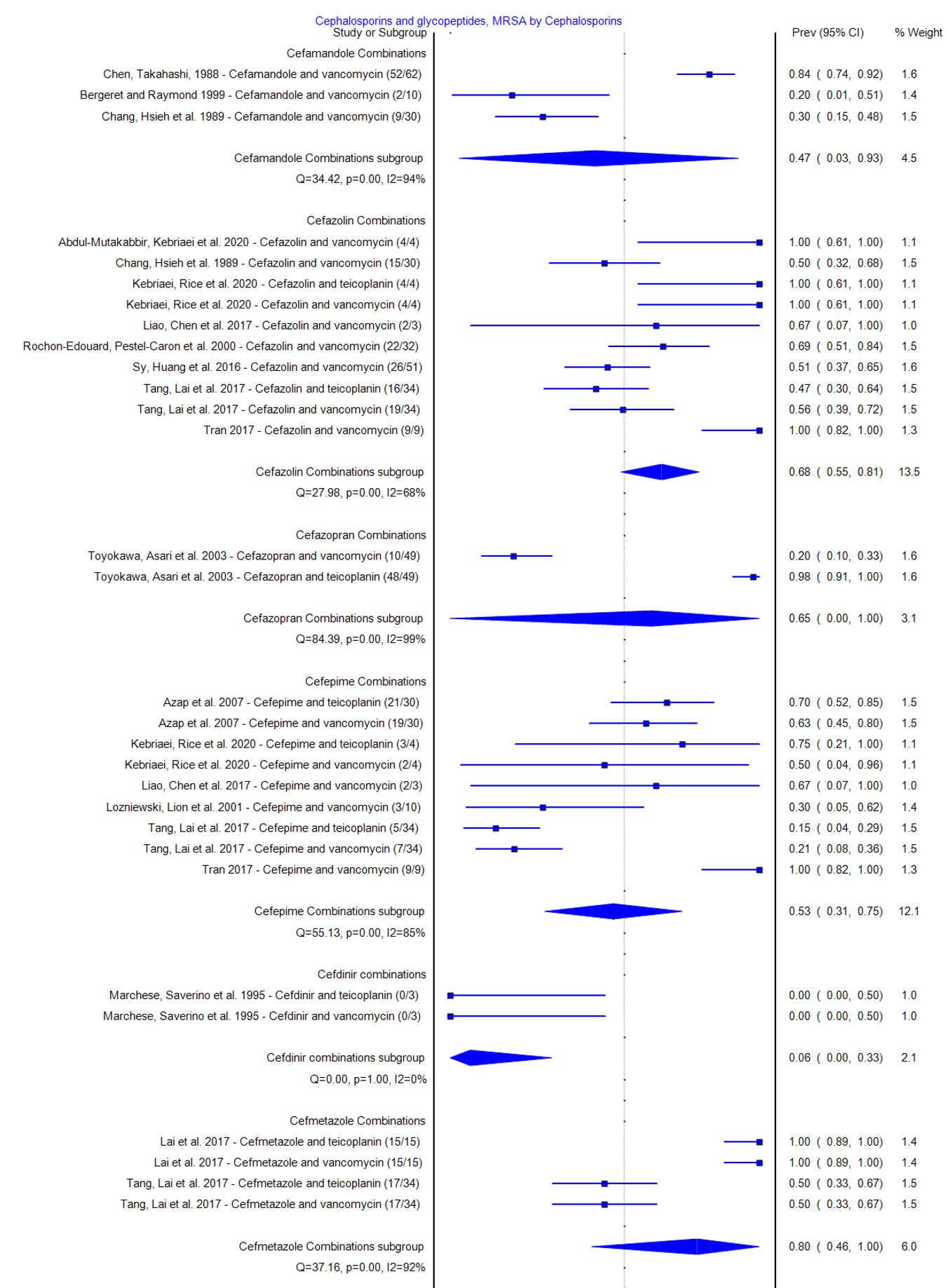


Figure continued on next page.

A continued


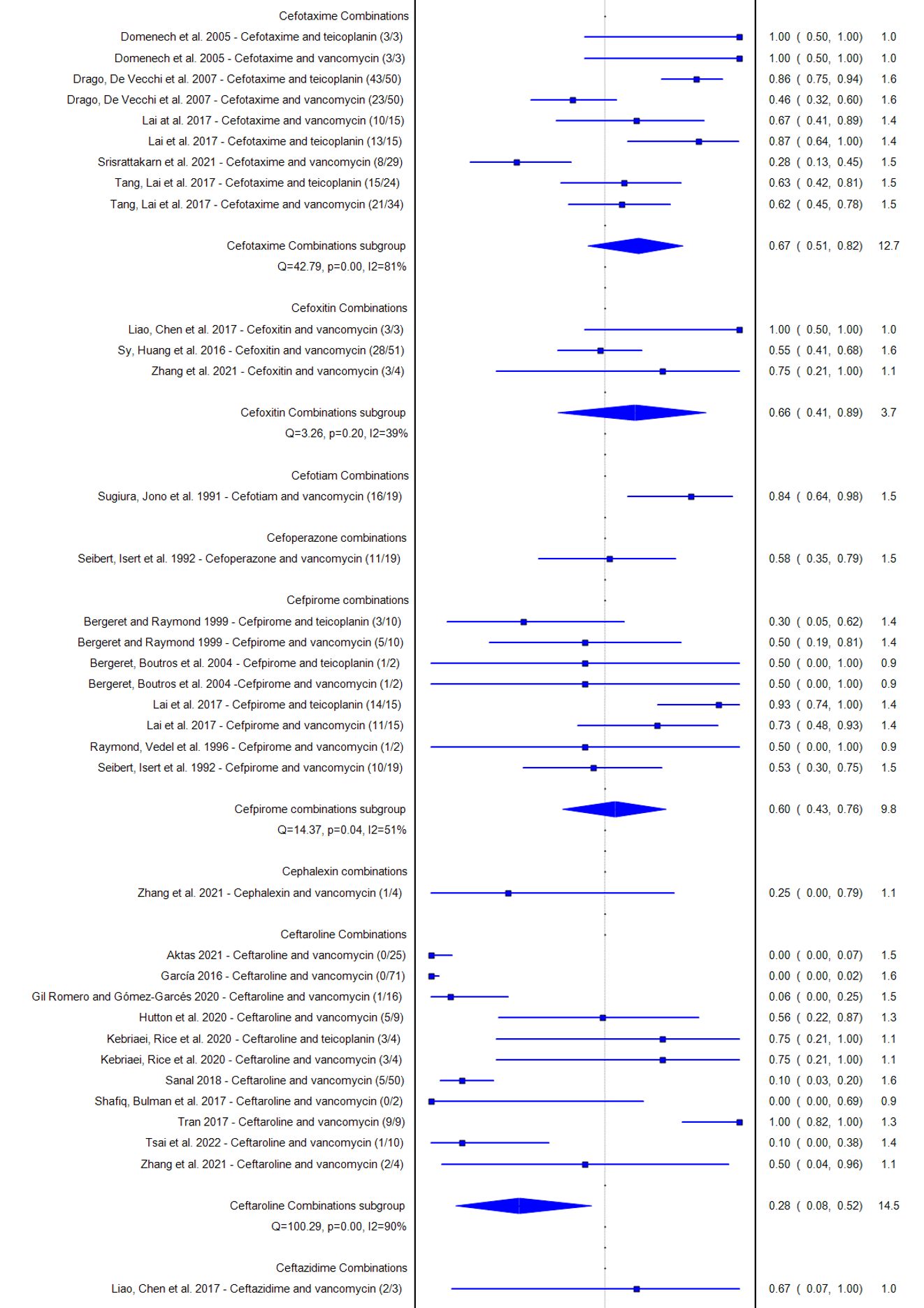


Figure continued on next page

A continued


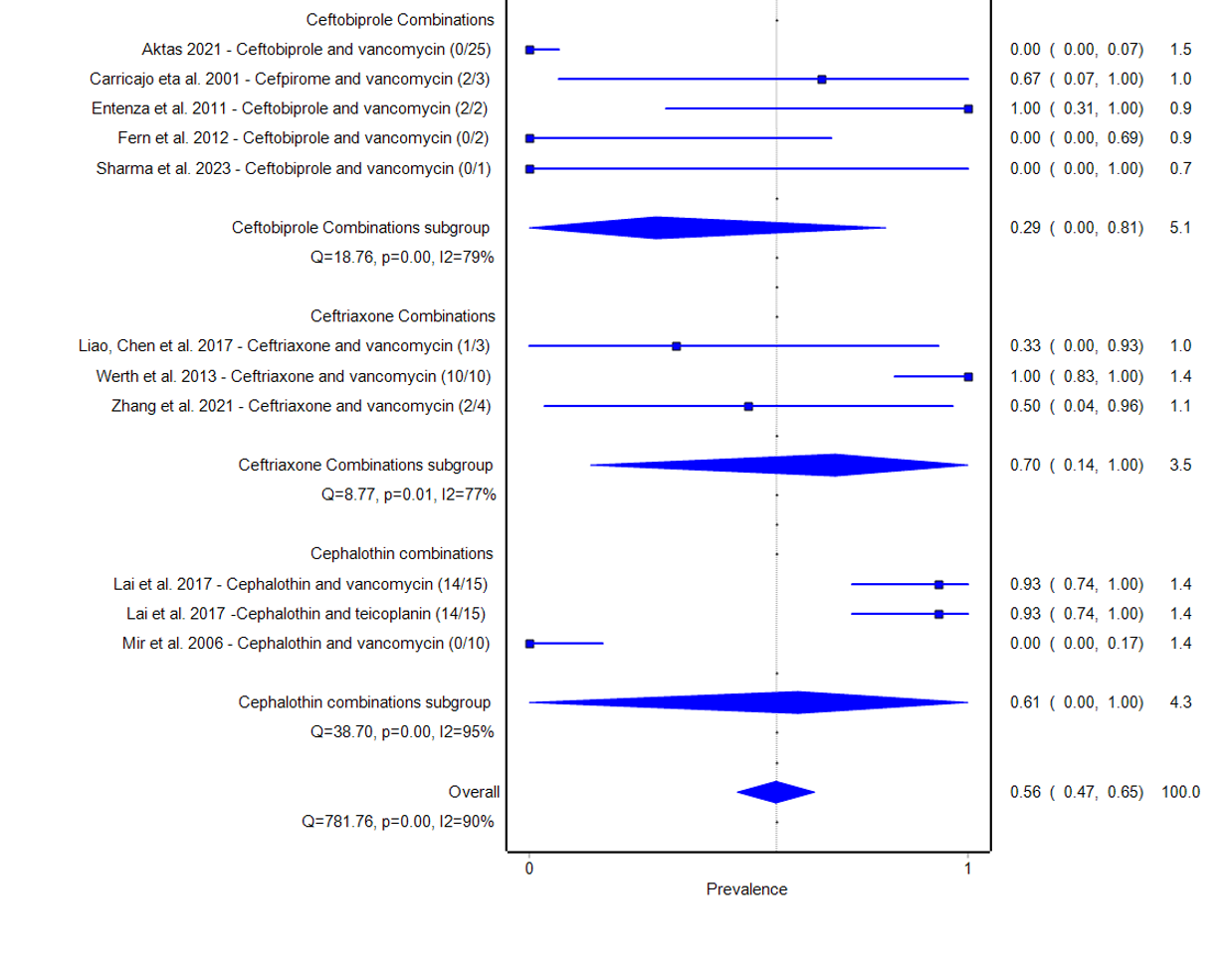


B


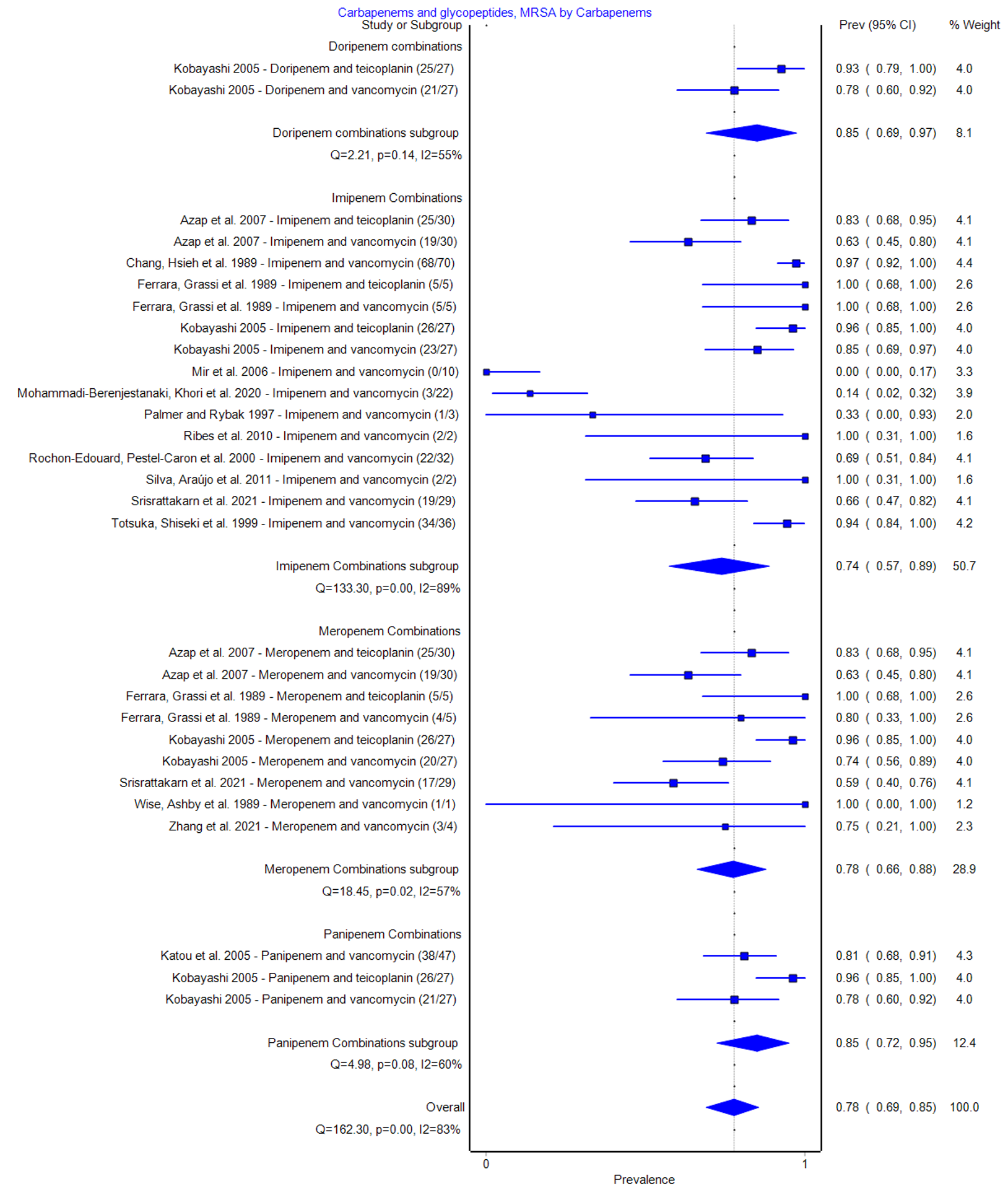


C


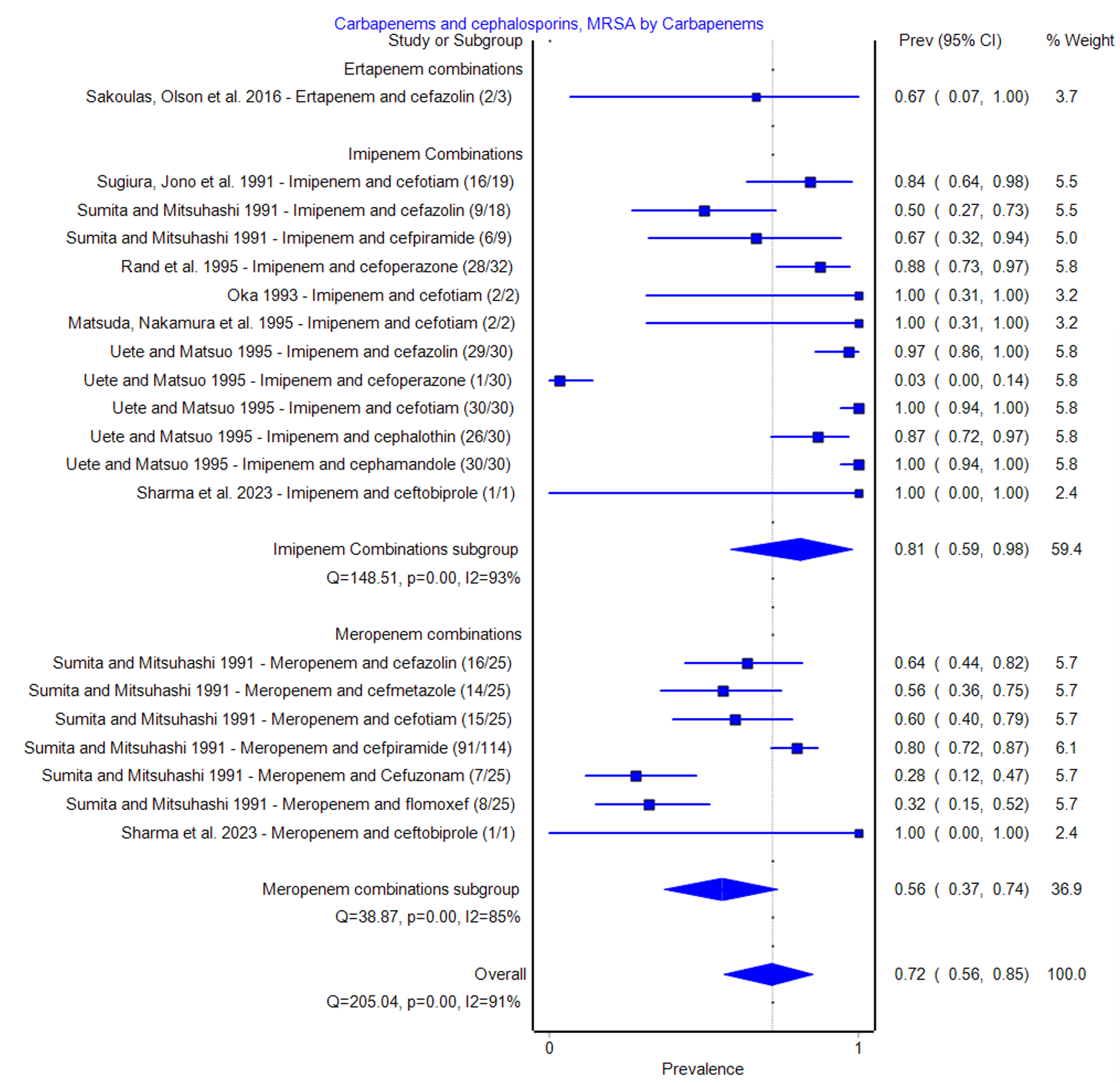


D


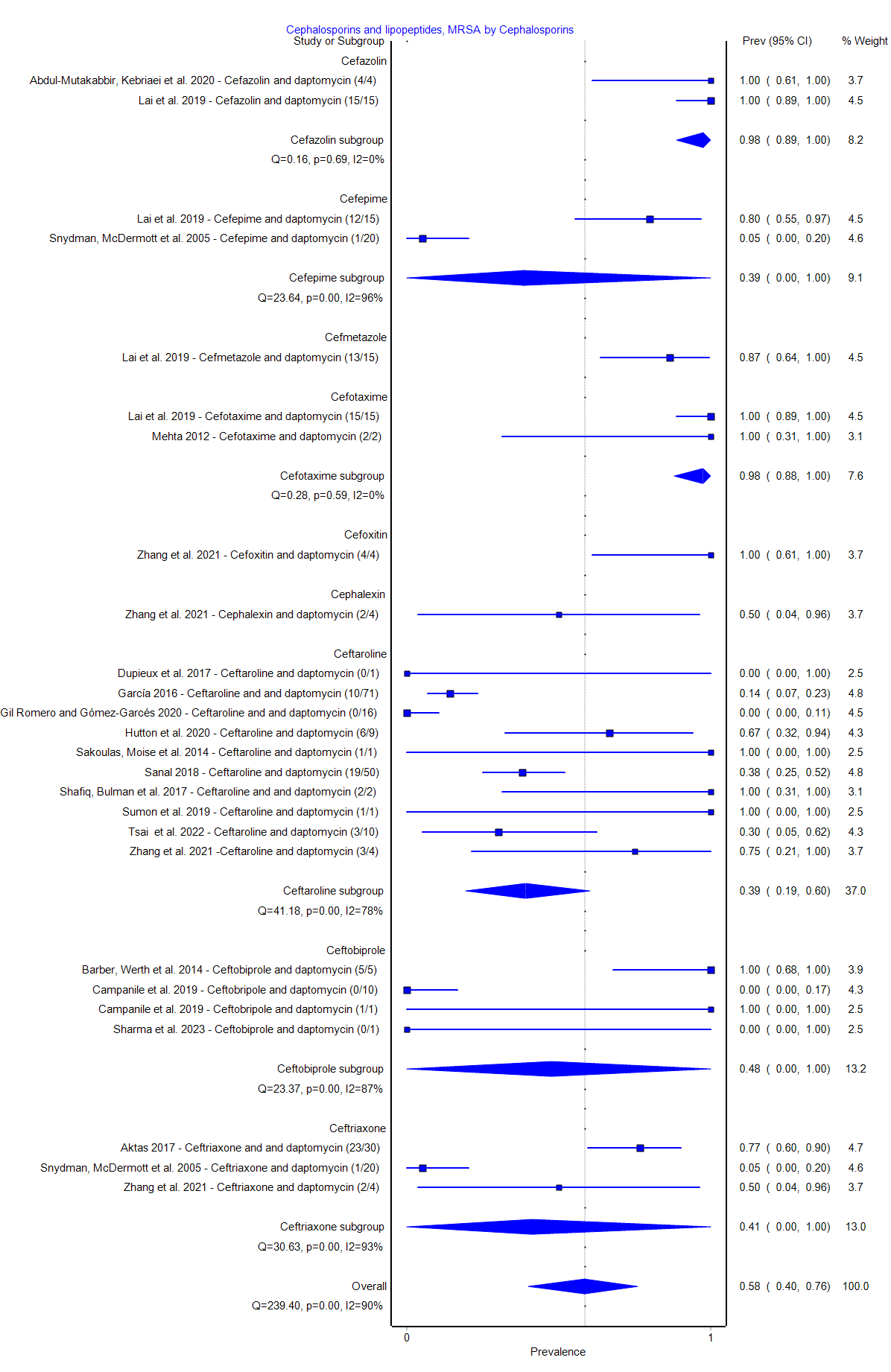


E


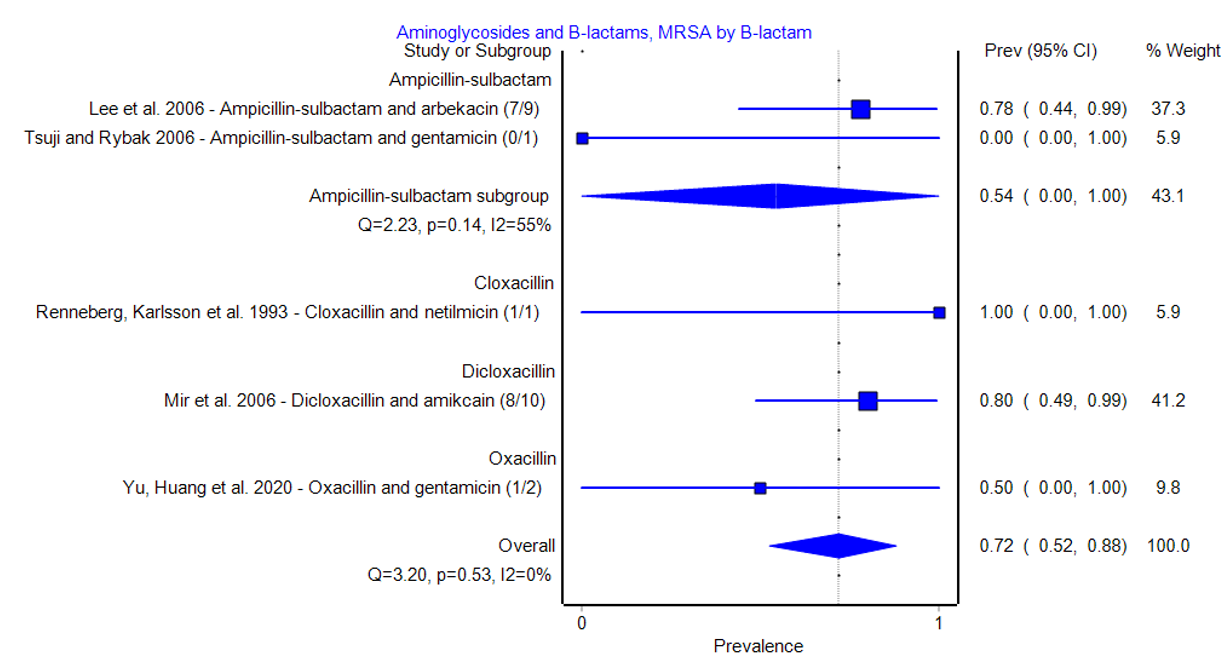


F


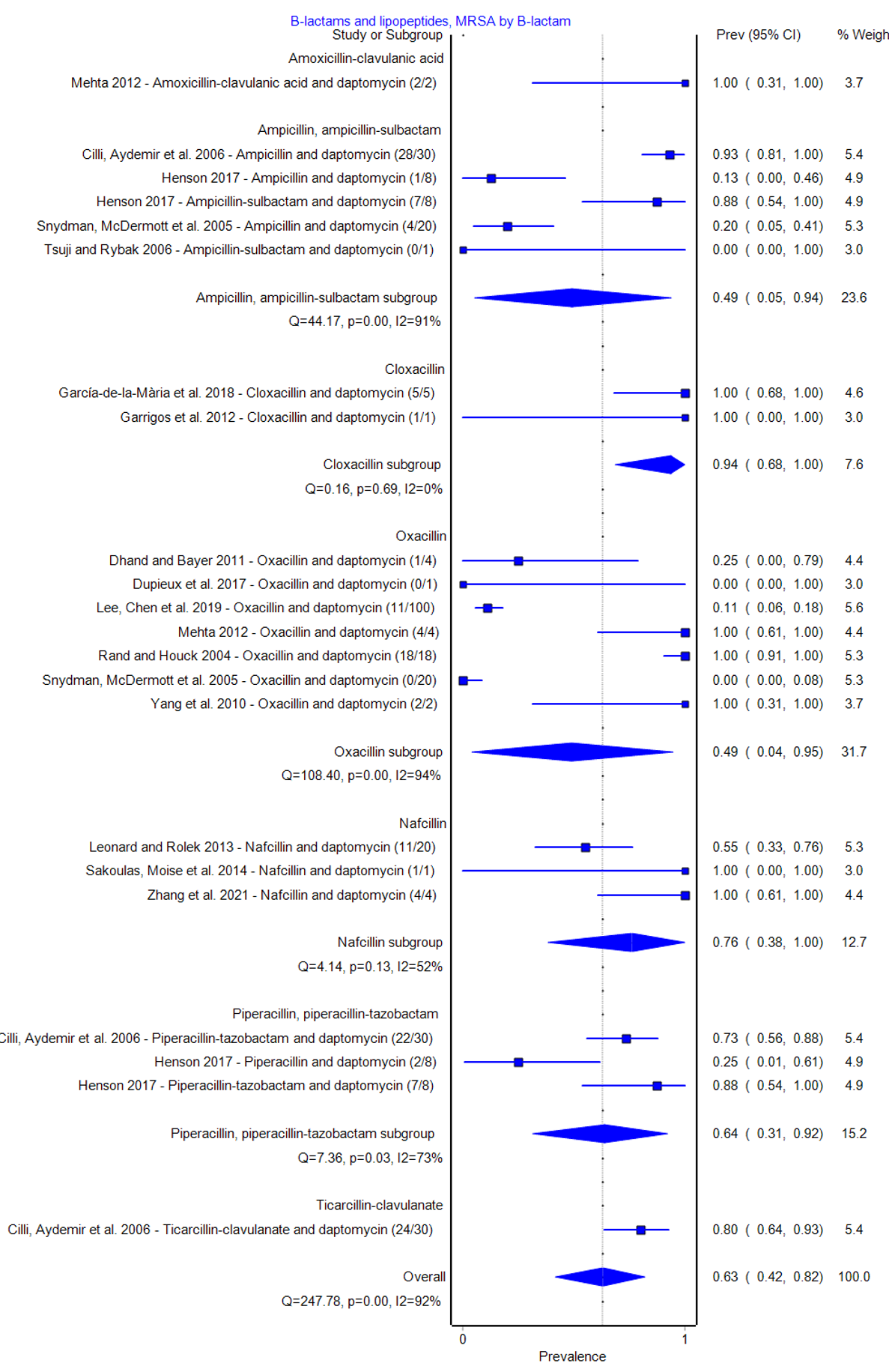


G


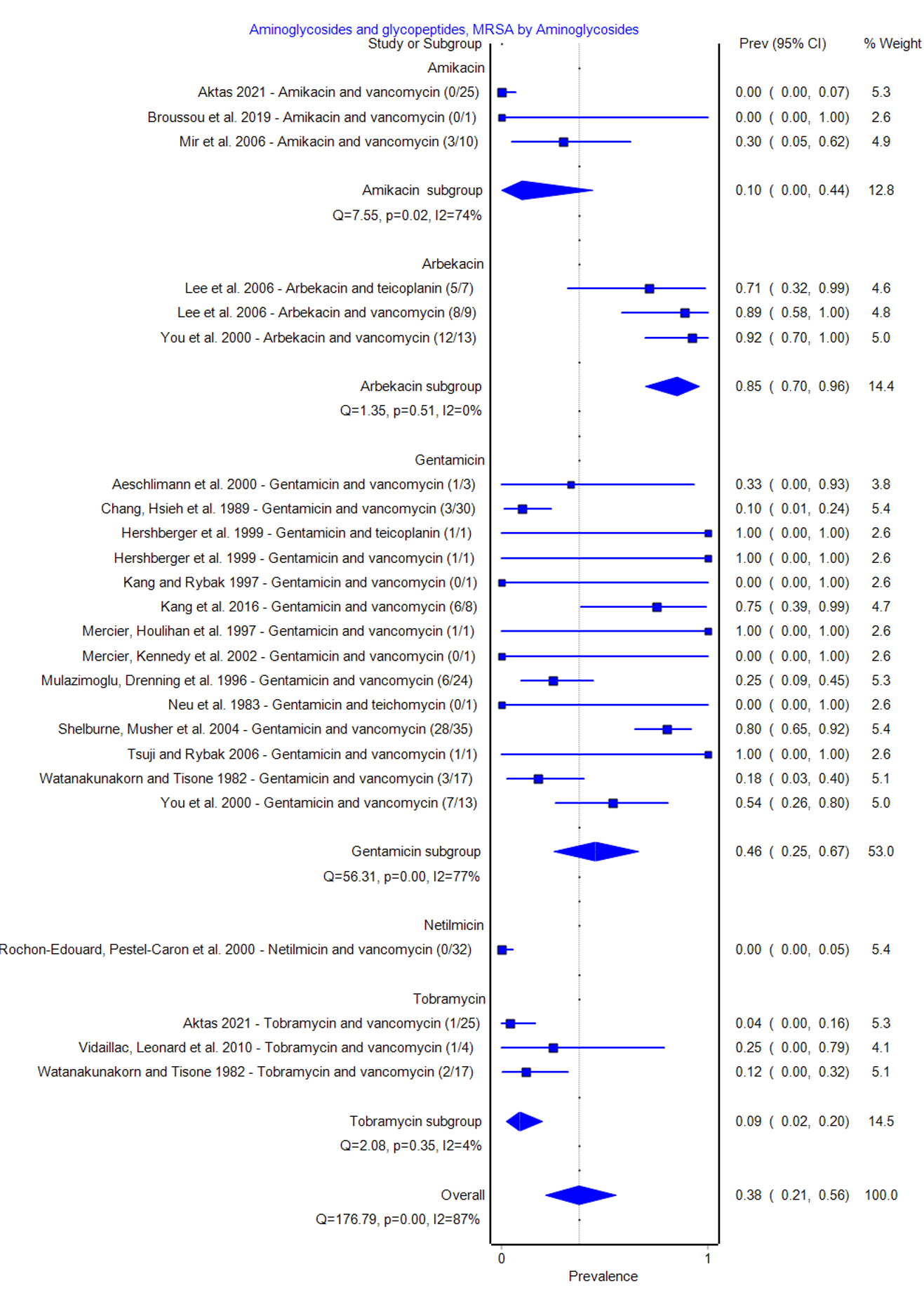


H

I


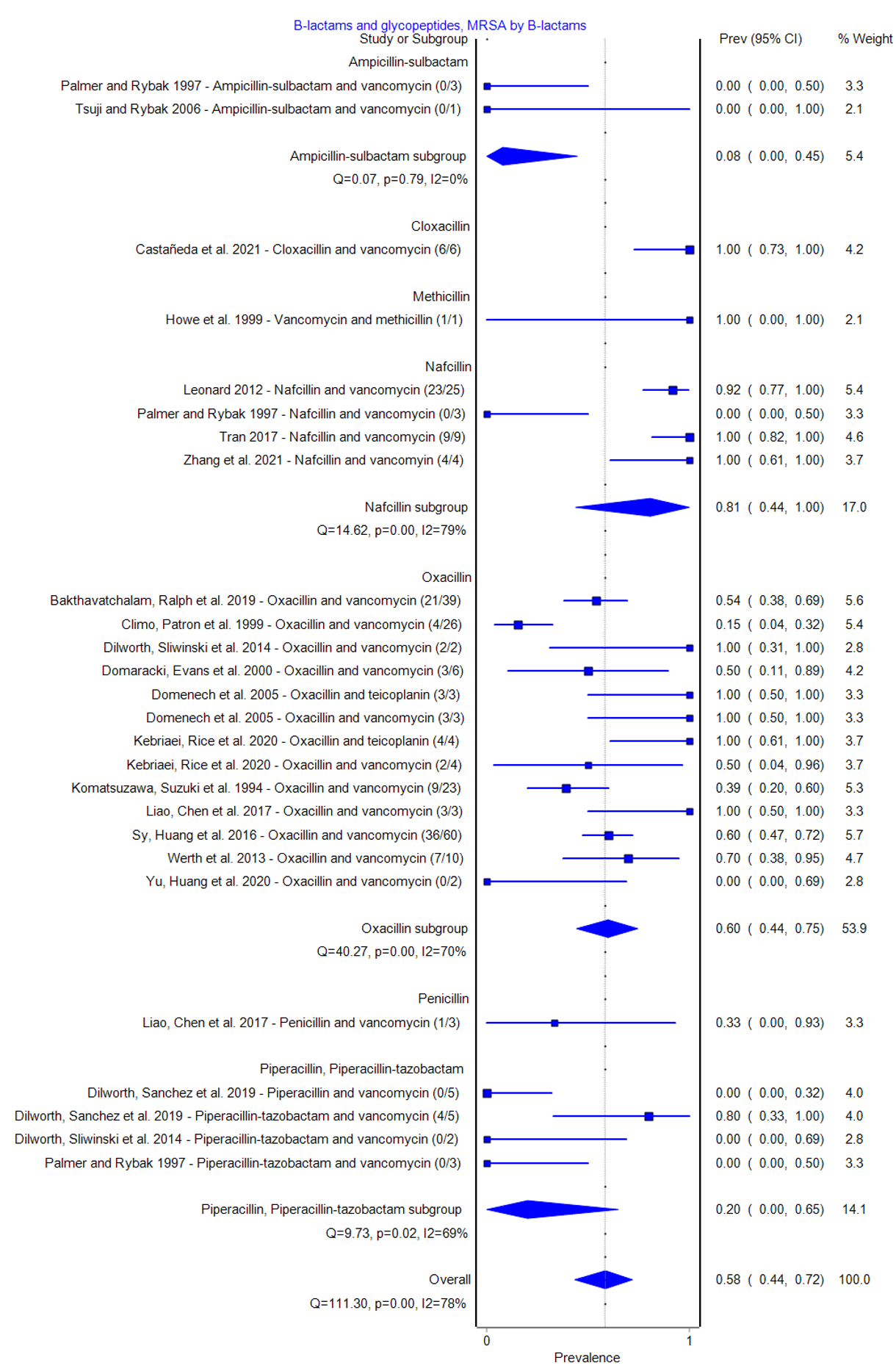


**Supplementary Figure 1**. Forest plots for meta-analysis of proportions for the top 10 most frequently studied combinations in this study against MRSA isolates.

1. Cephalosporins and glycopeptides
2. Carbapenems and glycopeptides
3. Carbapenems and cephalosporins
4. Cephalosporins and lipopeptides
5. Aminoglycosides and β-lactams
6. β-lactams and lipopeptides
7. Aminoglycosides and glycopeptides
8. Glycopeptides and rifamycins
9. β-lactams and glycopeptides

*See Figure 2C in the main text for the combination of Cephalosporins and fosfomycin, the tenth most frequently tested antibiotic combination.

A


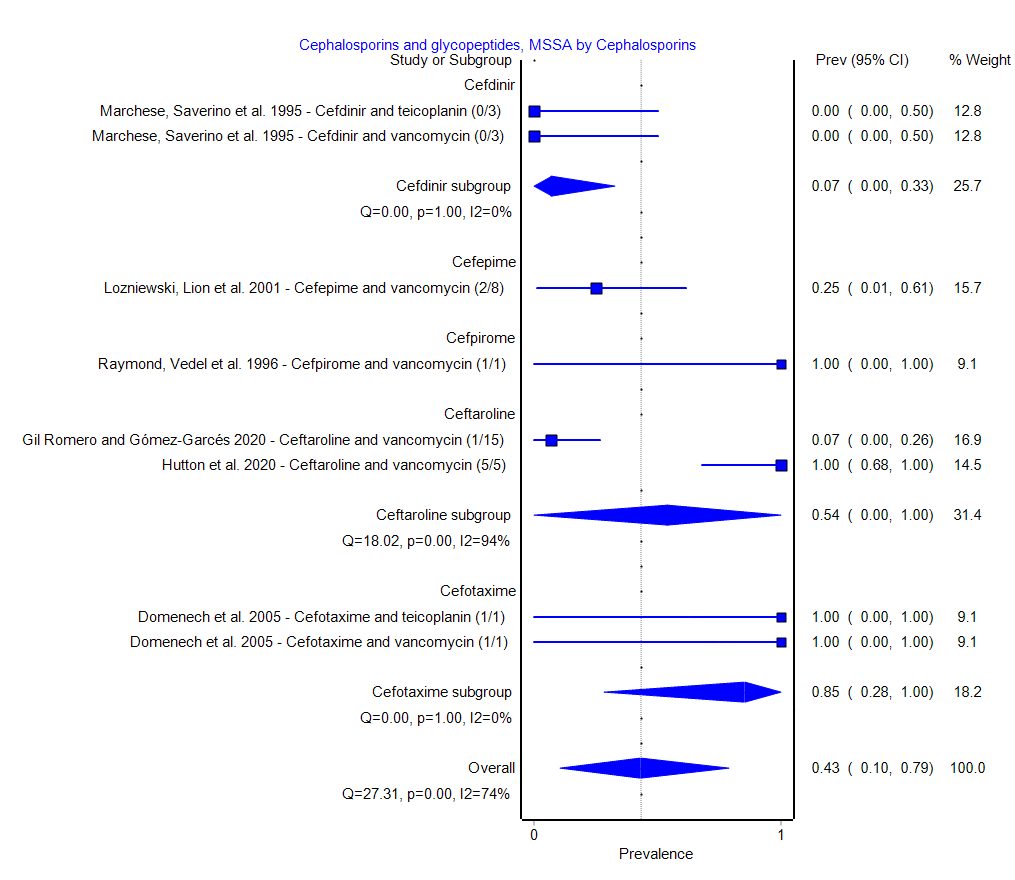


B


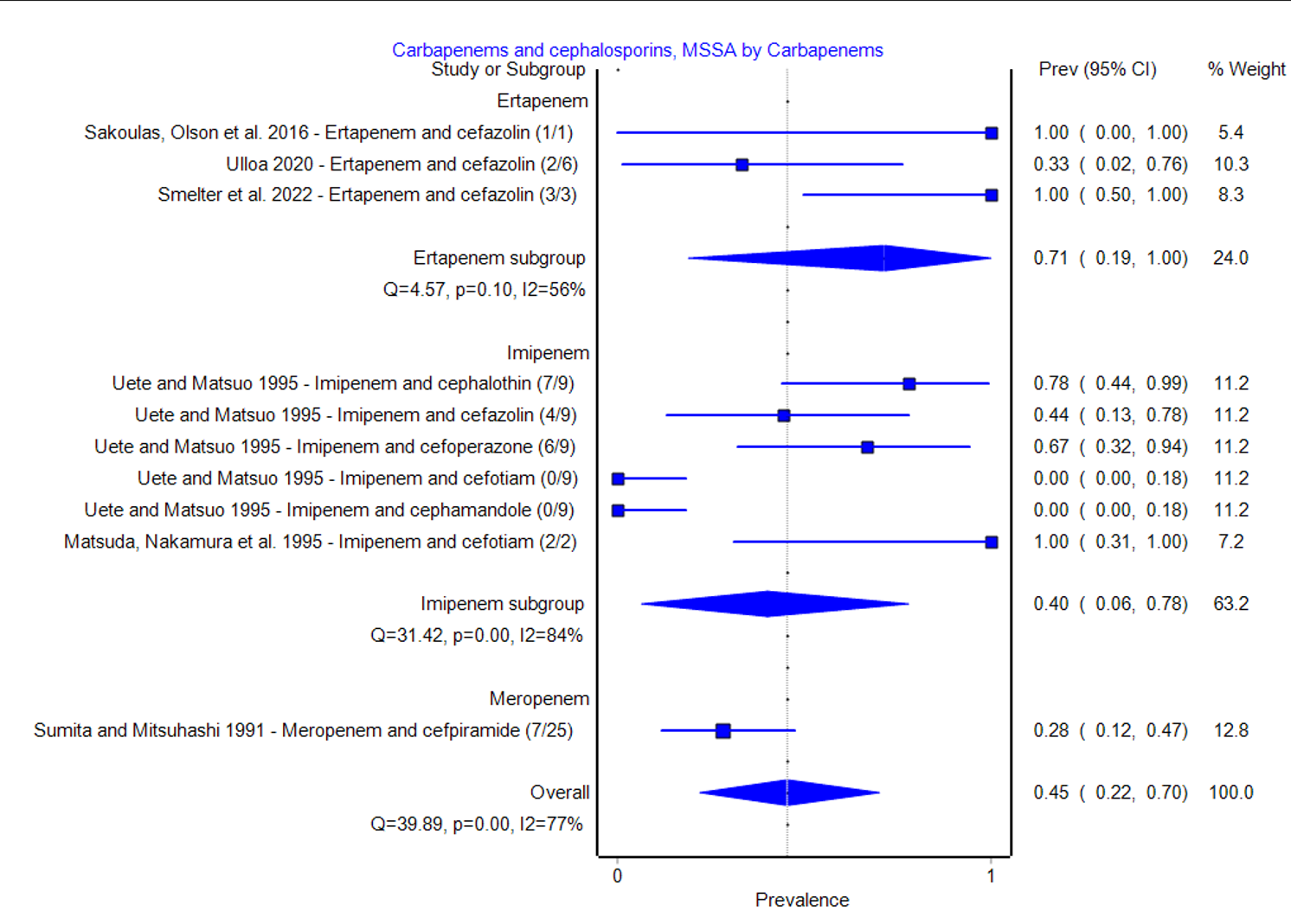


C

D


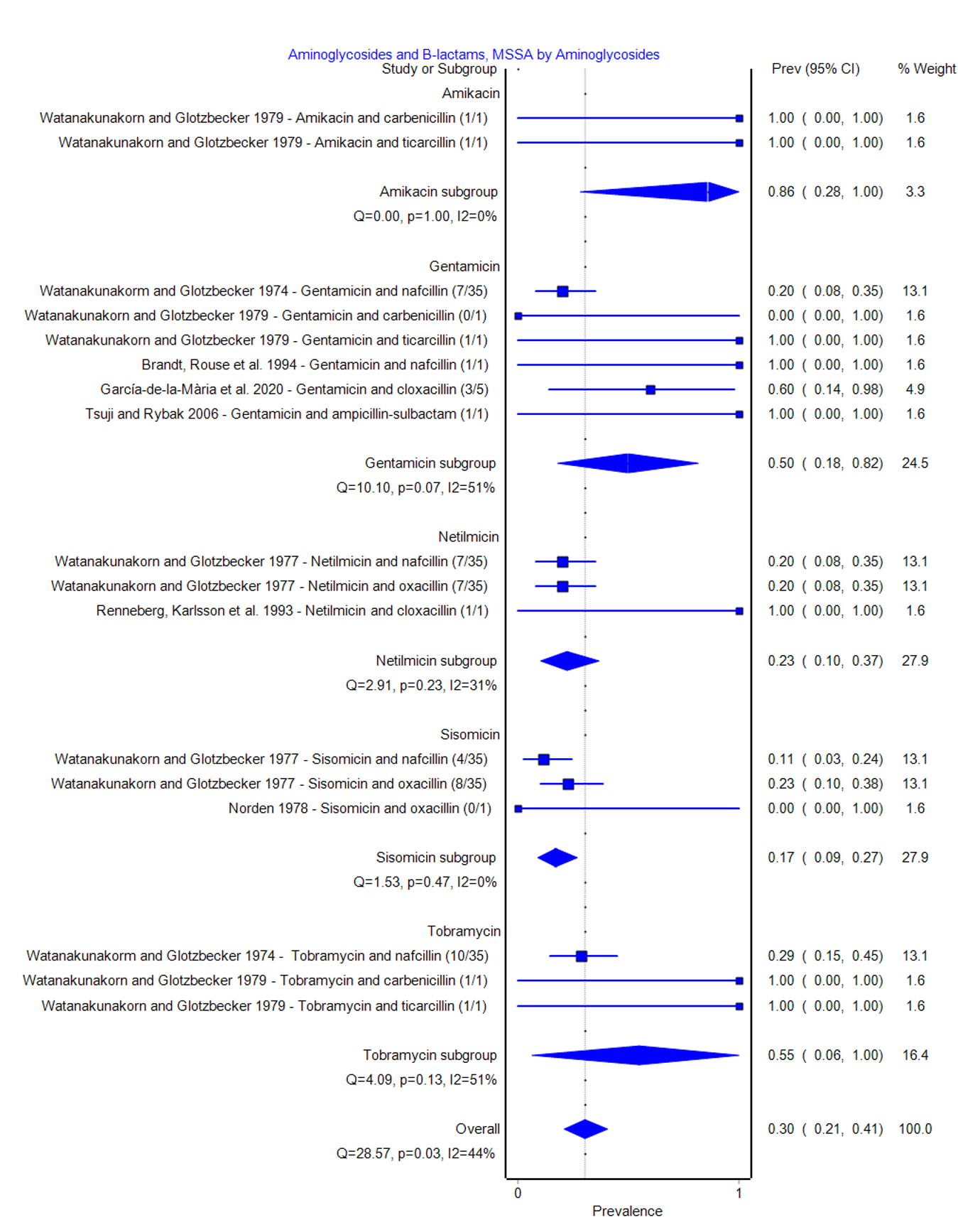


E


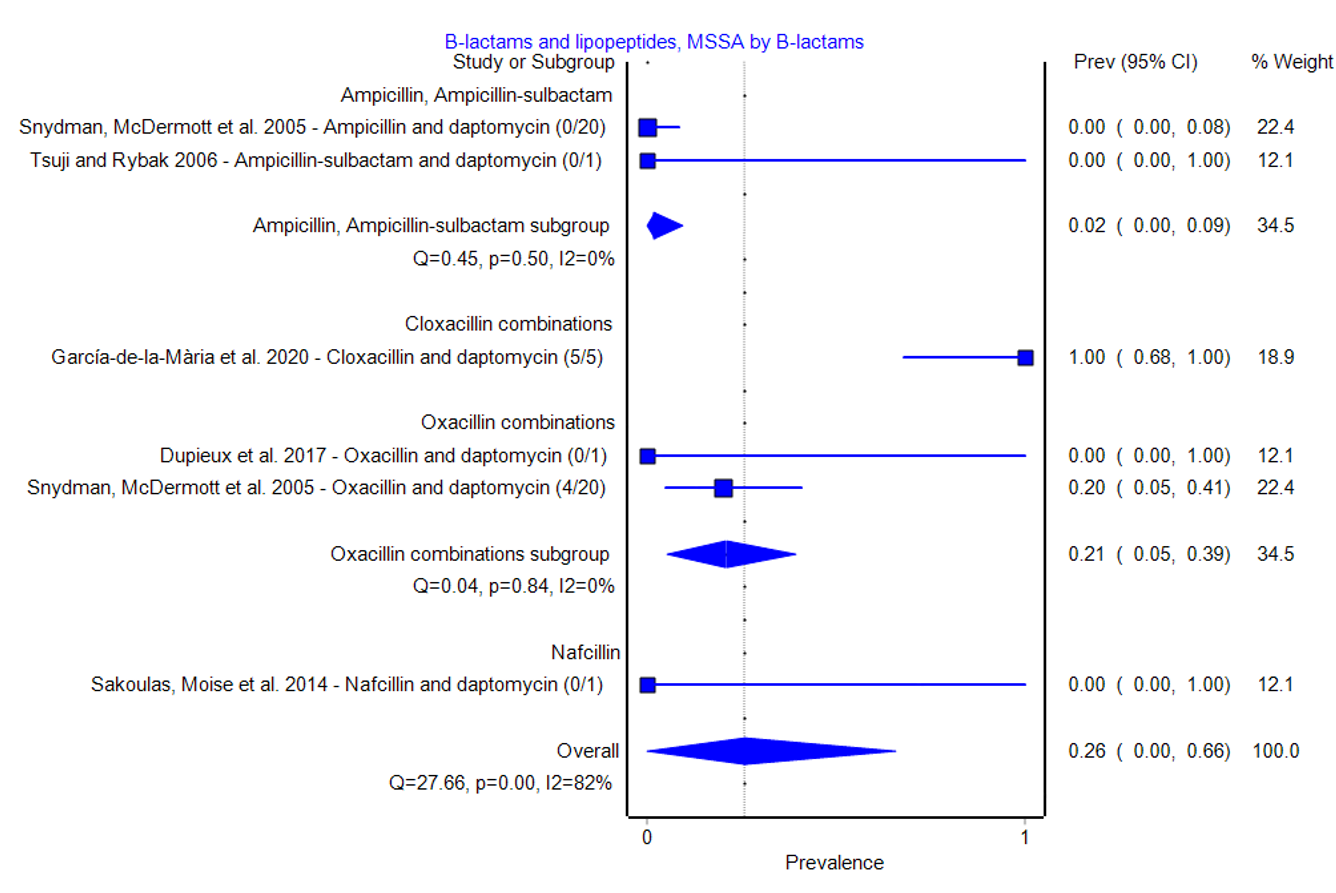


F


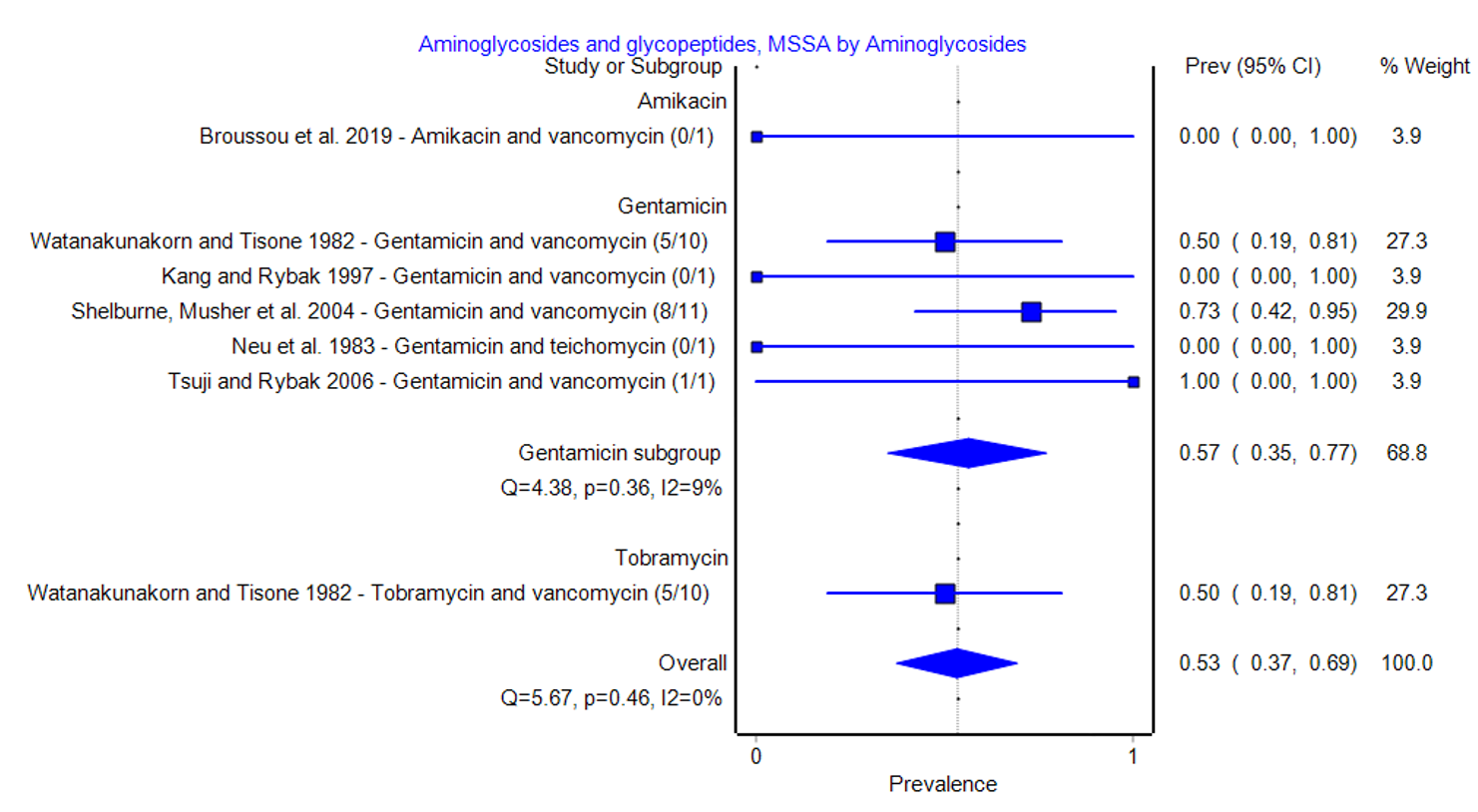


G


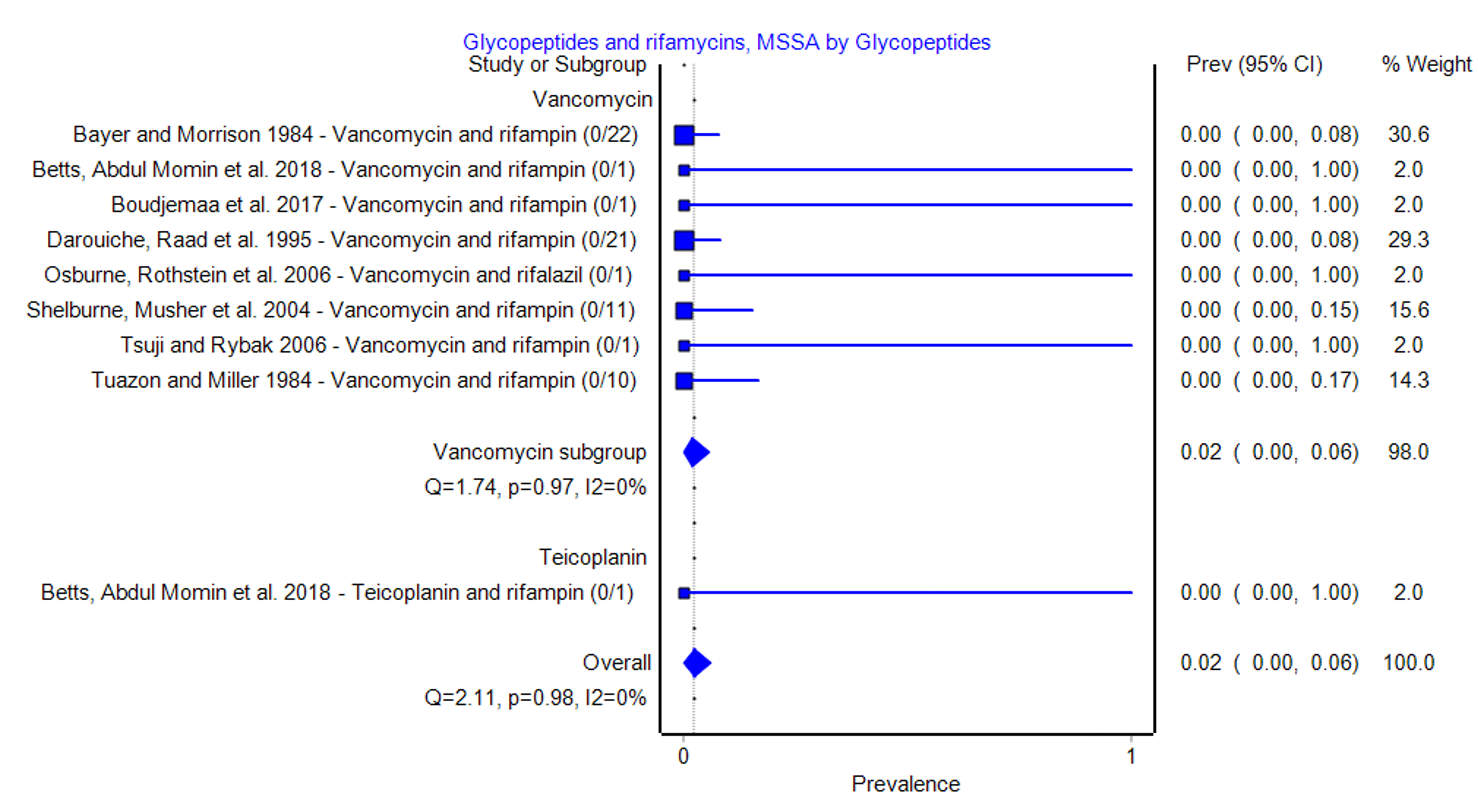


H


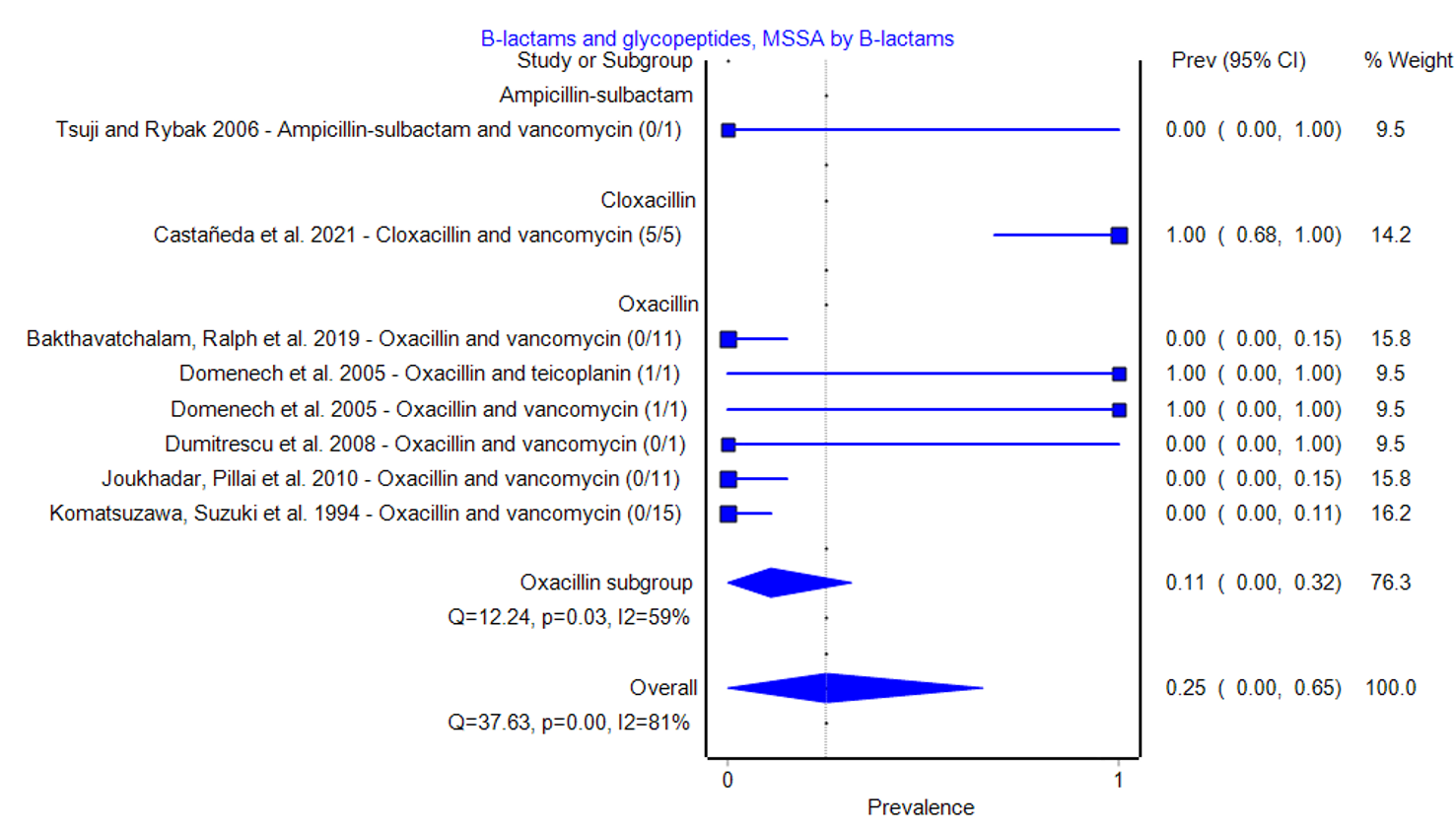


I

**Supplementary Figure 2**. Forest plots for meta-analysis of proportions for the top 10 most frequently studied combinations in this study against MSSA isolates.

1. Cephalosporins and glycopeptides
2. Carbapenems and cephalosporins
3. Cephalosporins and lipopeptides
4. Aminoglycosides and β-lactams
5. β-lactams and lipopeptides
6. Aminoglycosides and glycopeptides
7. Glycopeptides and rifamycins
8. β-lactams and glycopeptides
9. Cephalosporins and fosfomycin

*See Figure 3A in the main text for the combination of carbapenems and glycopeptides, the second most frequently tested combination

A

B


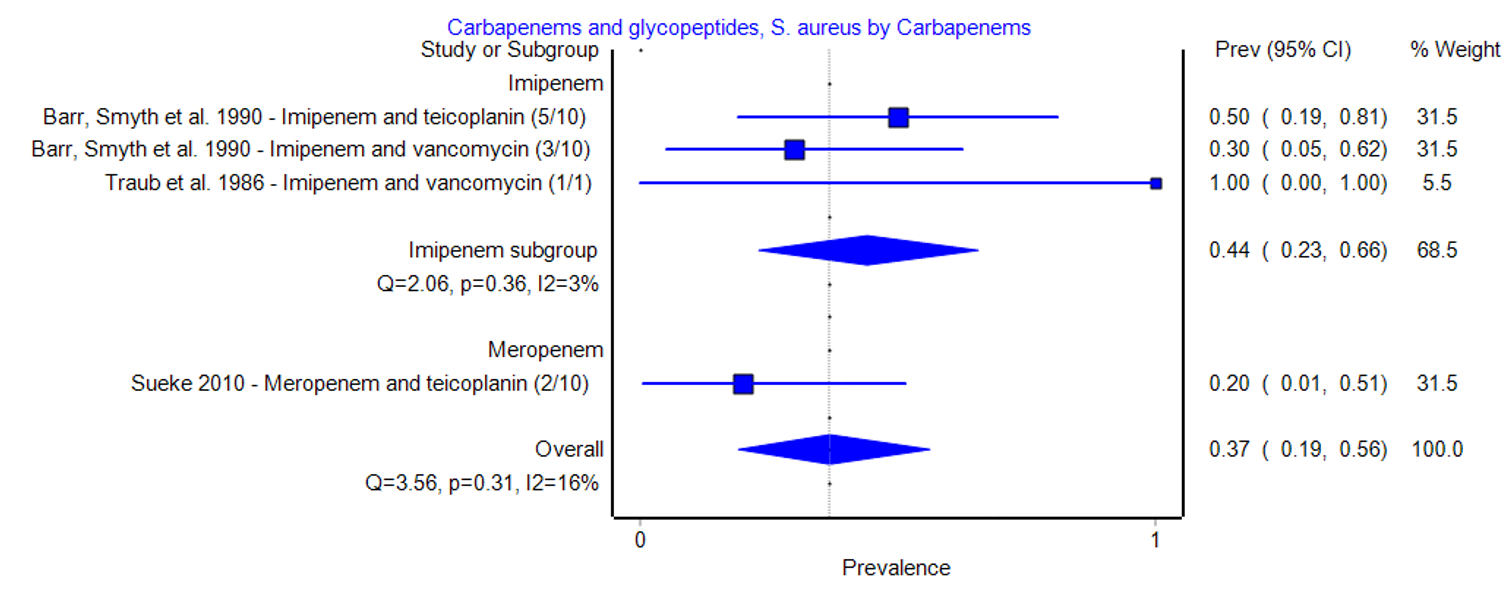


C


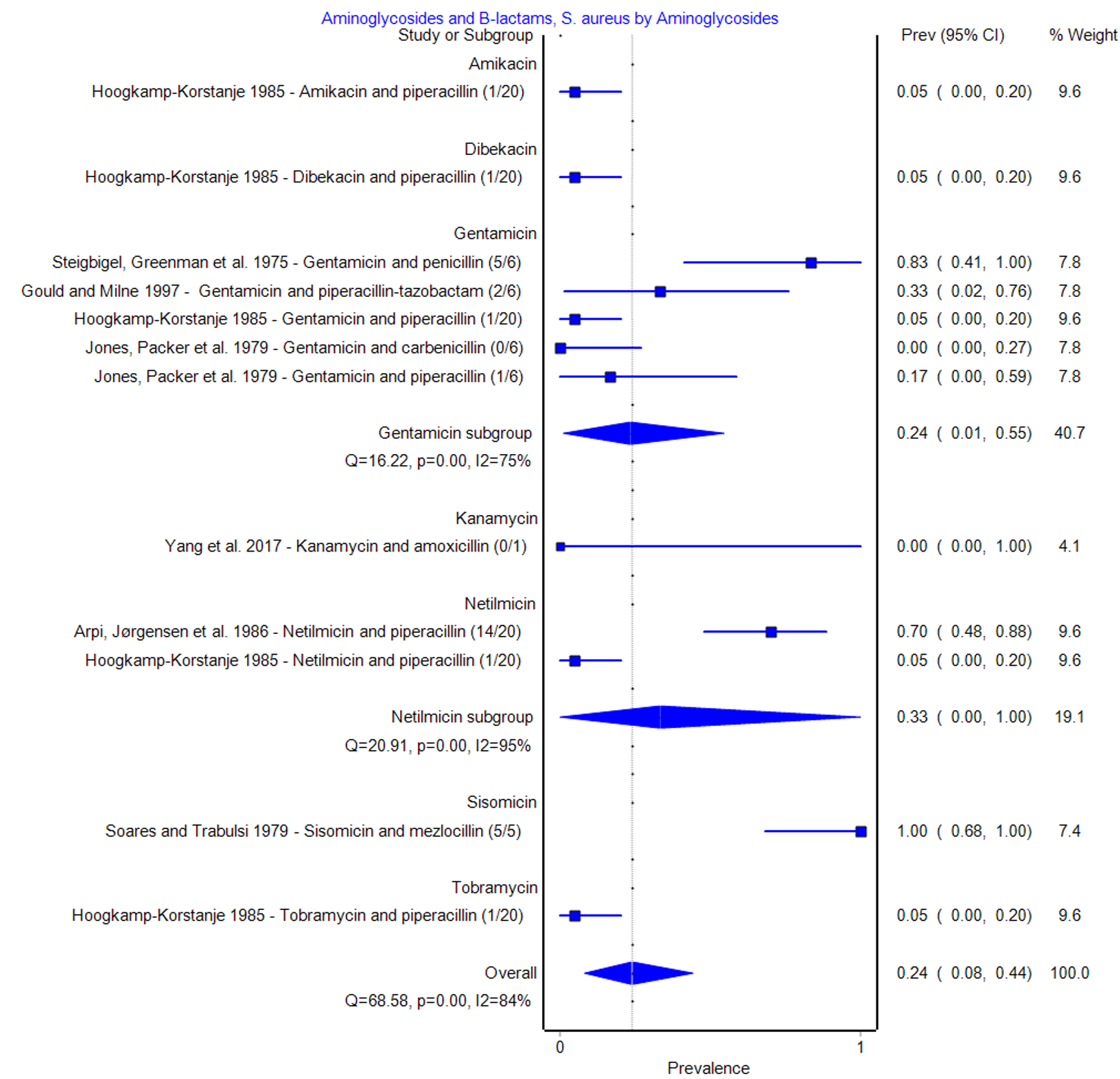


D

E

**Supplementary Figure 3**. Forest plots for meta-analysis of proportions for the top 10 most frequently studied combinations in this study against *S. aureus* isolates.

1. Cephalosporins and glycopeptides
2. Carbapenems and glycopeptides
3. Aminoglycosides and β-lactams
4. Aminoglycosides and glycopeptides
5. Glycopeptides and rifamycins

*Other combinations in the top 10 frequently studied combinations were not studied for isolates in the *S. aureus* category


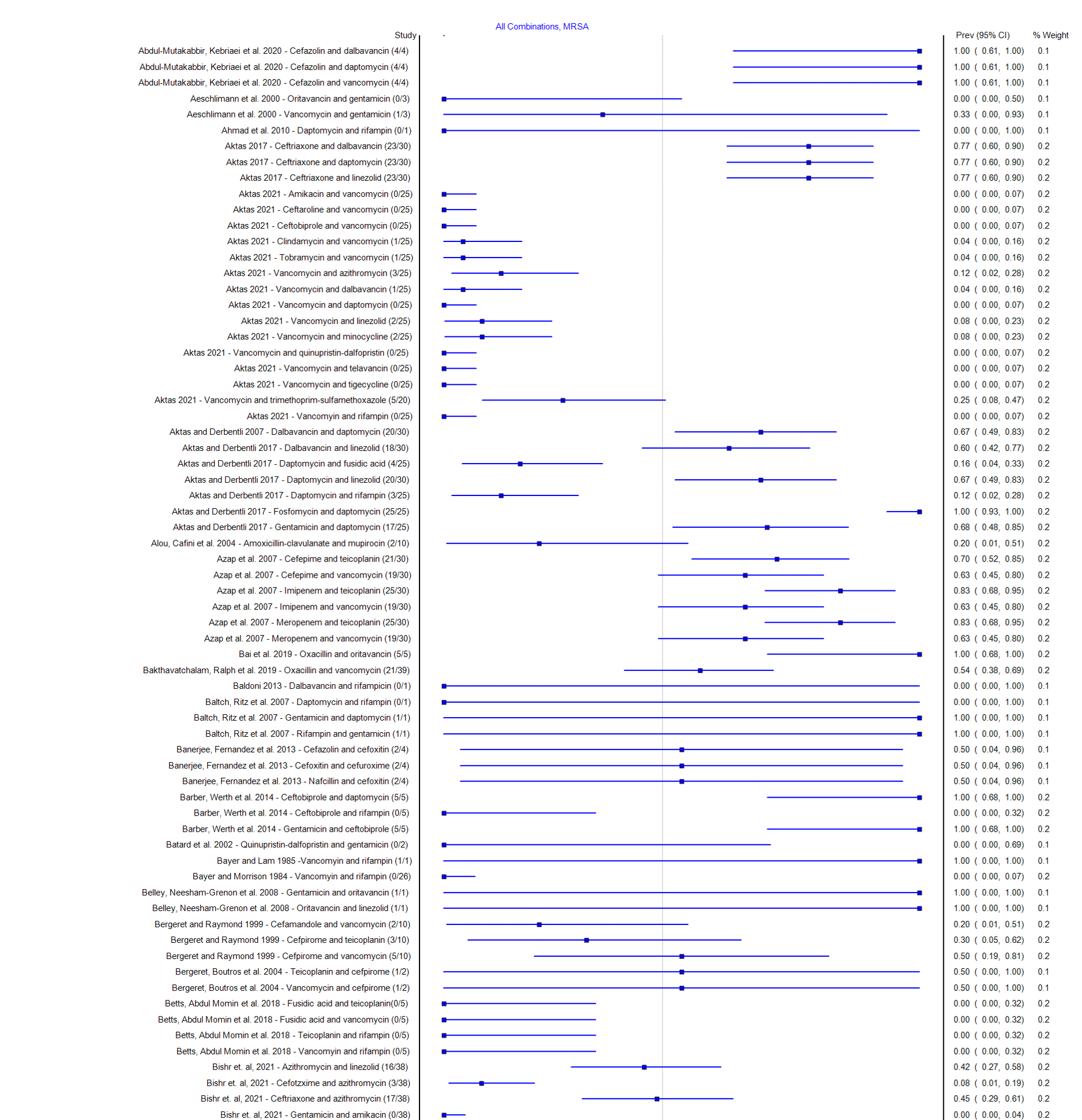
Figure continued on next page


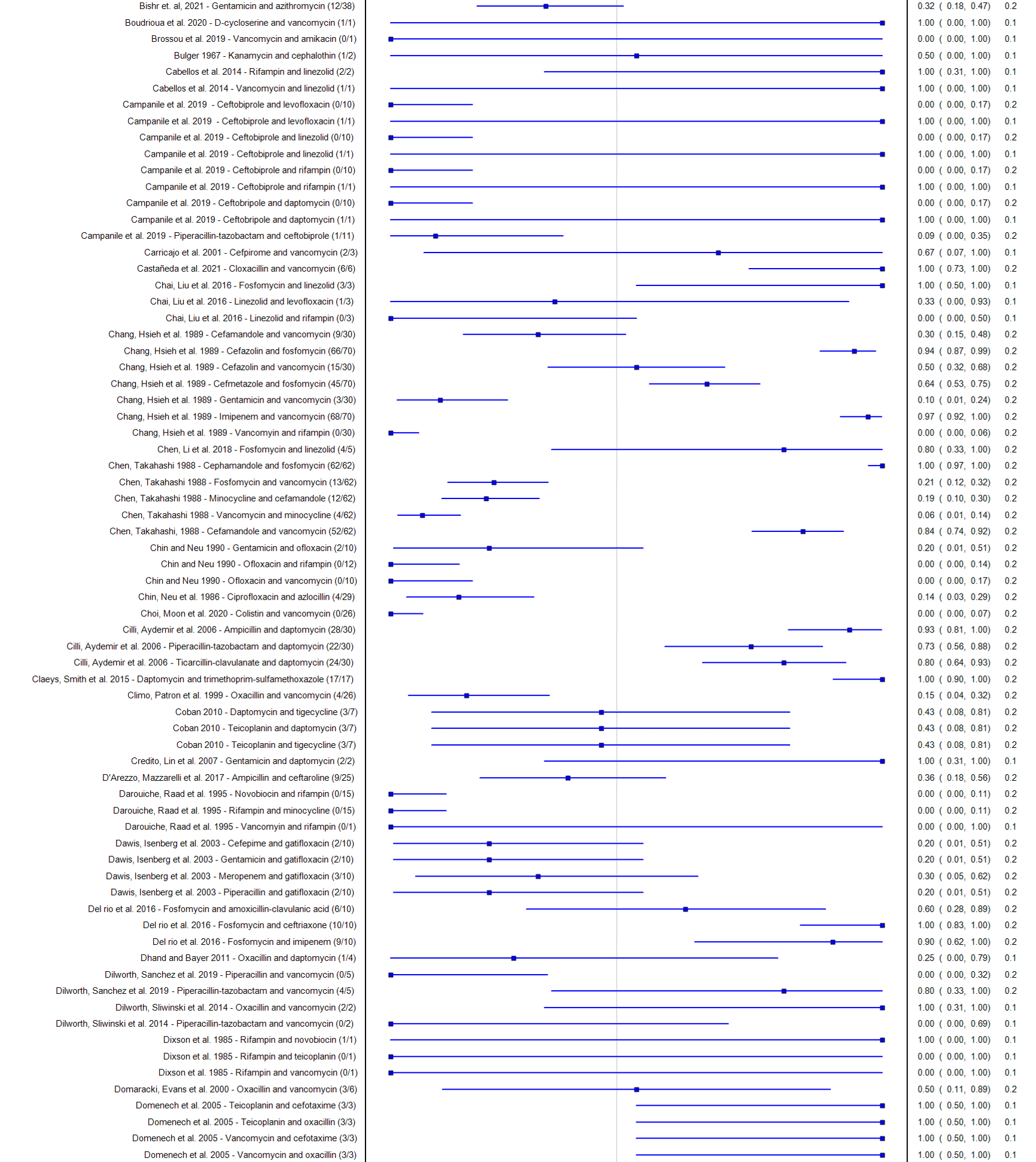
Figure continued on next page


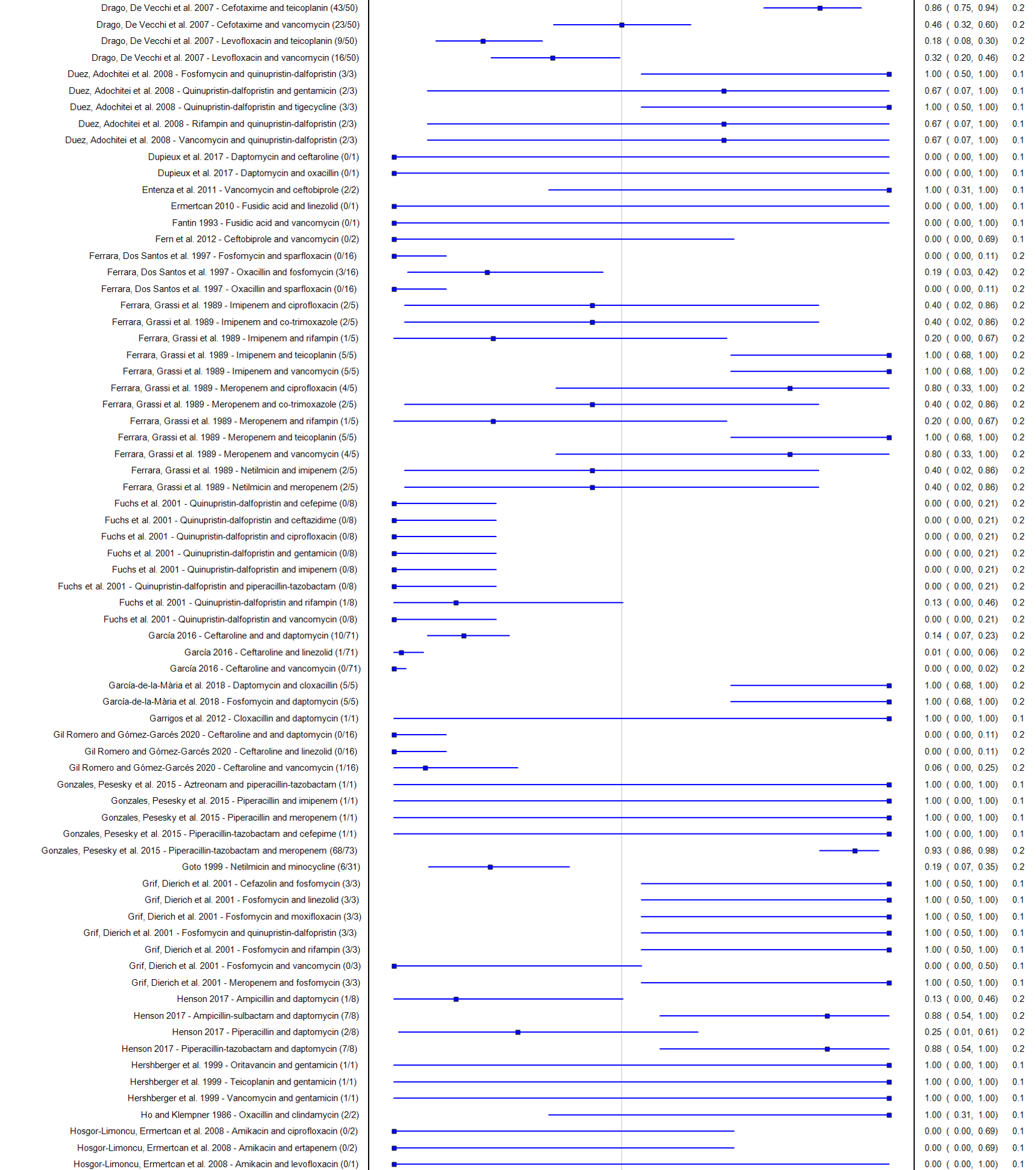
Figure continued on next page


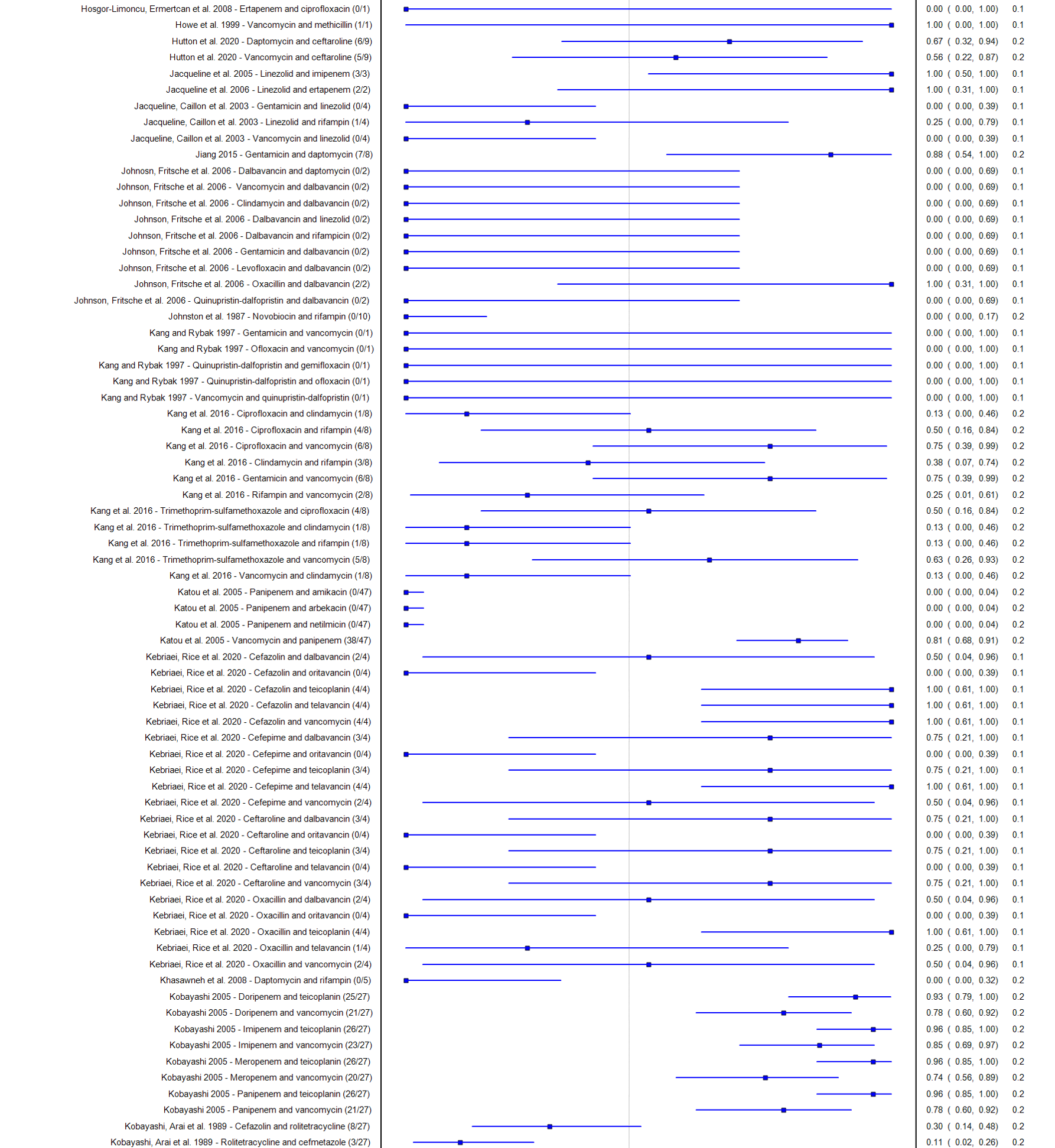
Figure continued on next page


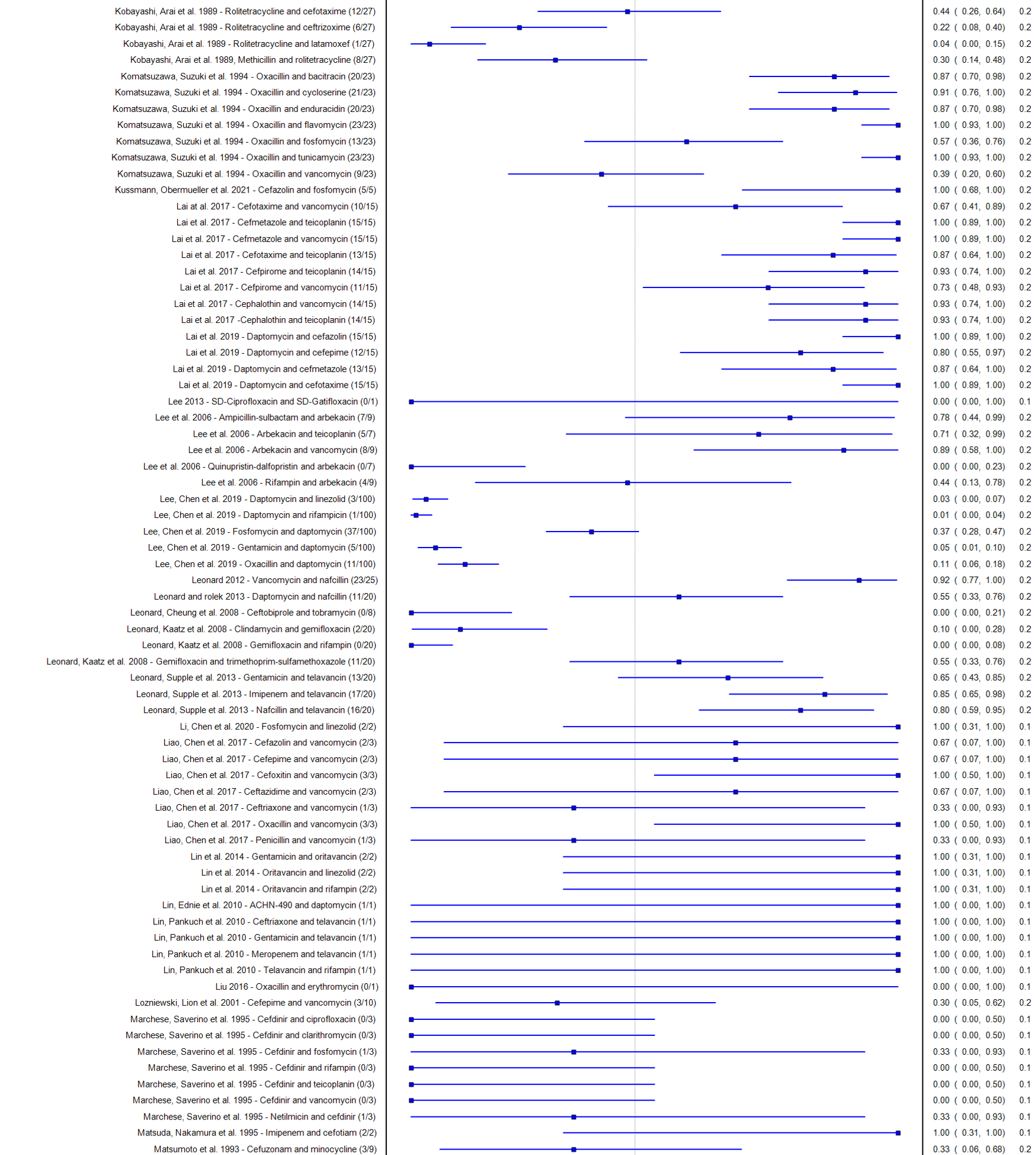
Figure continued on next page


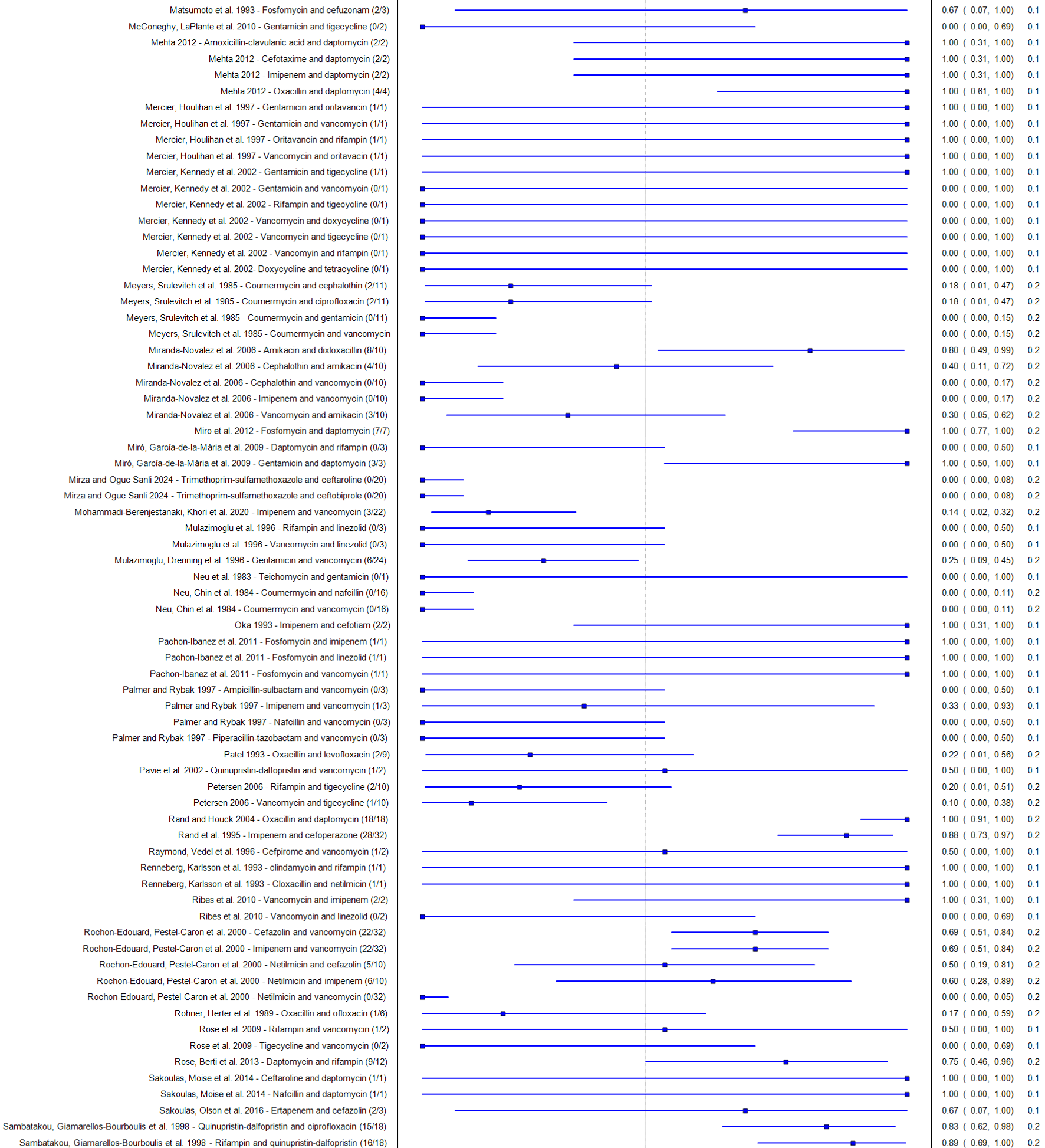
Figure continued on next page


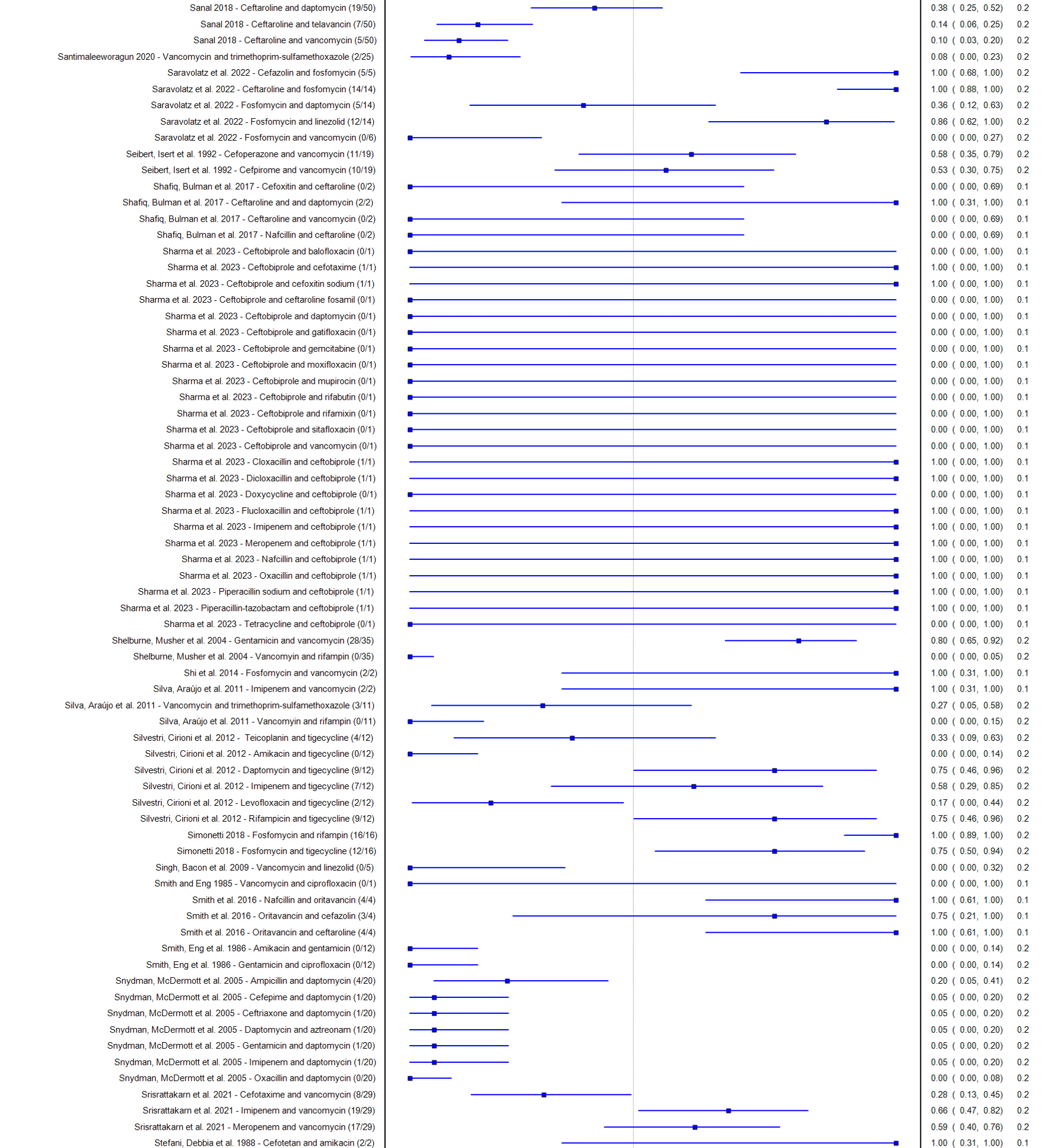
 Figure continued on next page


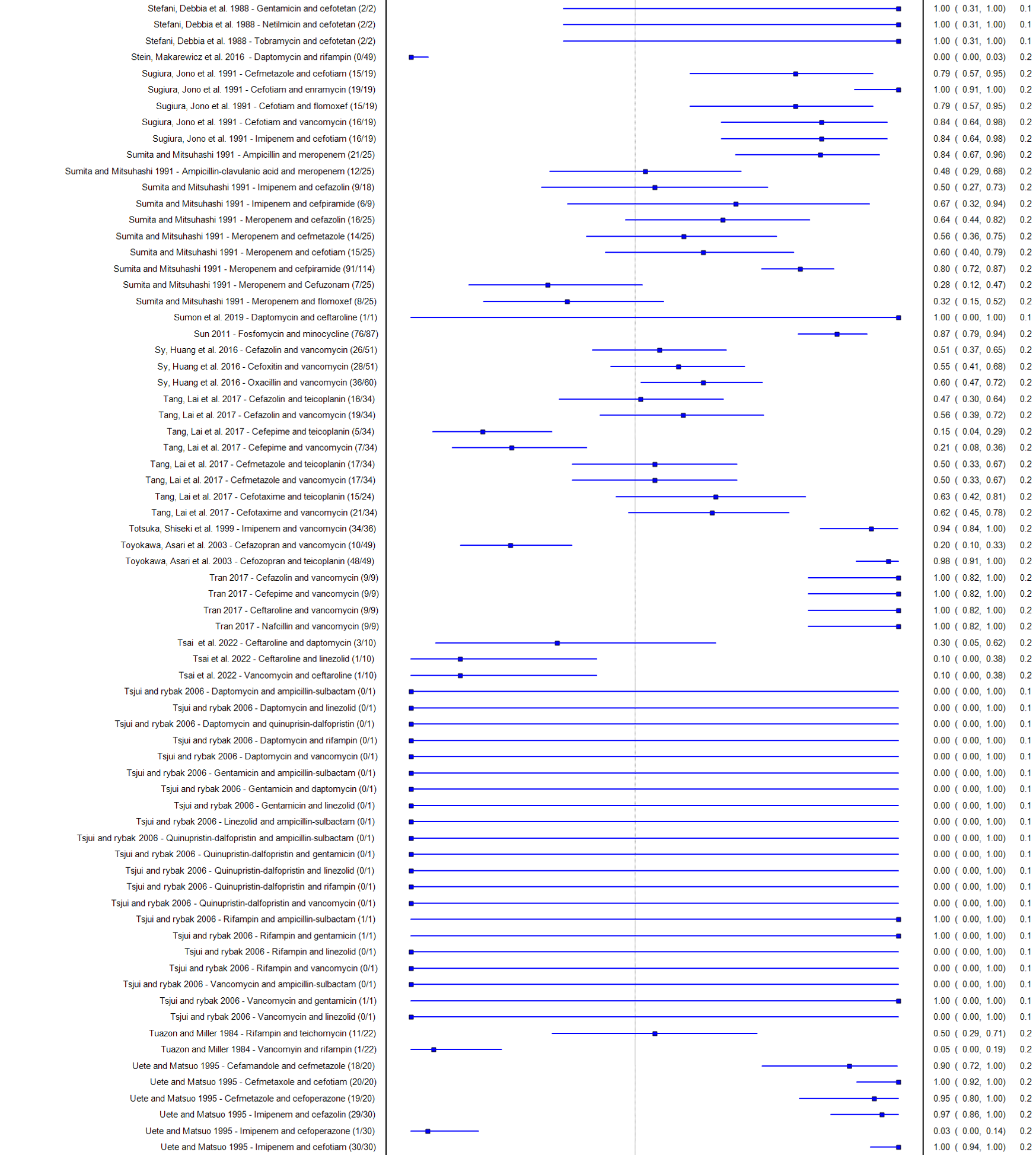
Figure continued on next page


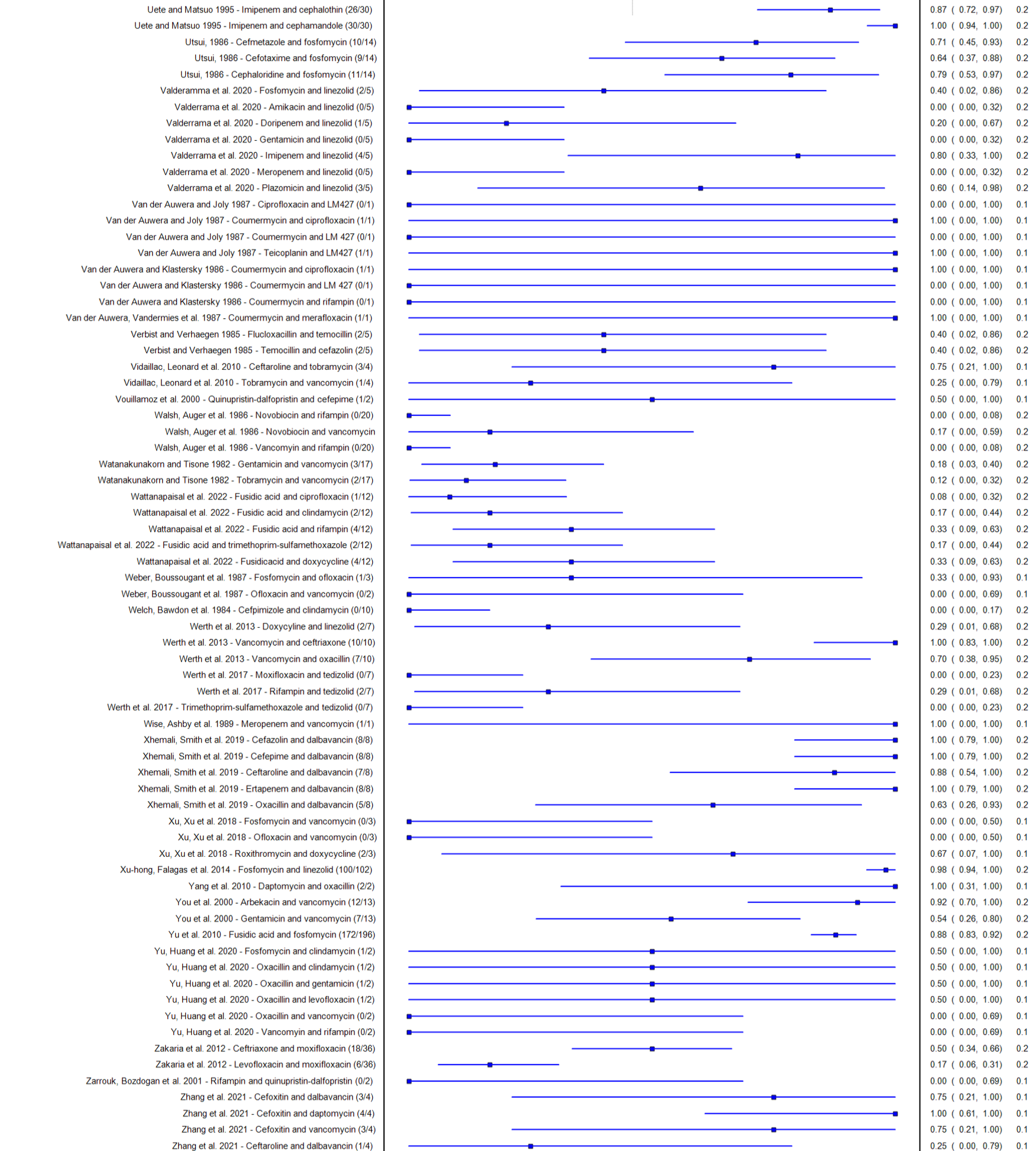
Figure continued on next page


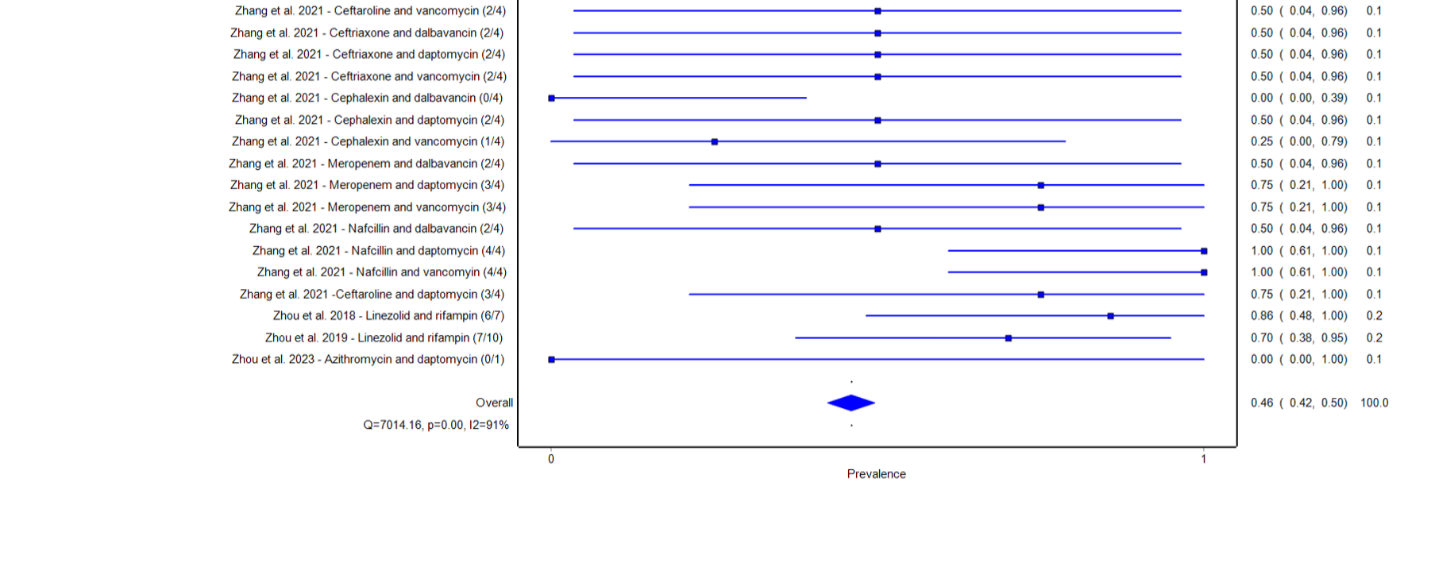
**Supplementary Figure 4.** Forrest plots for meta-analysis of proportions of all tested antimicrobial combinations for MRSA isolates.


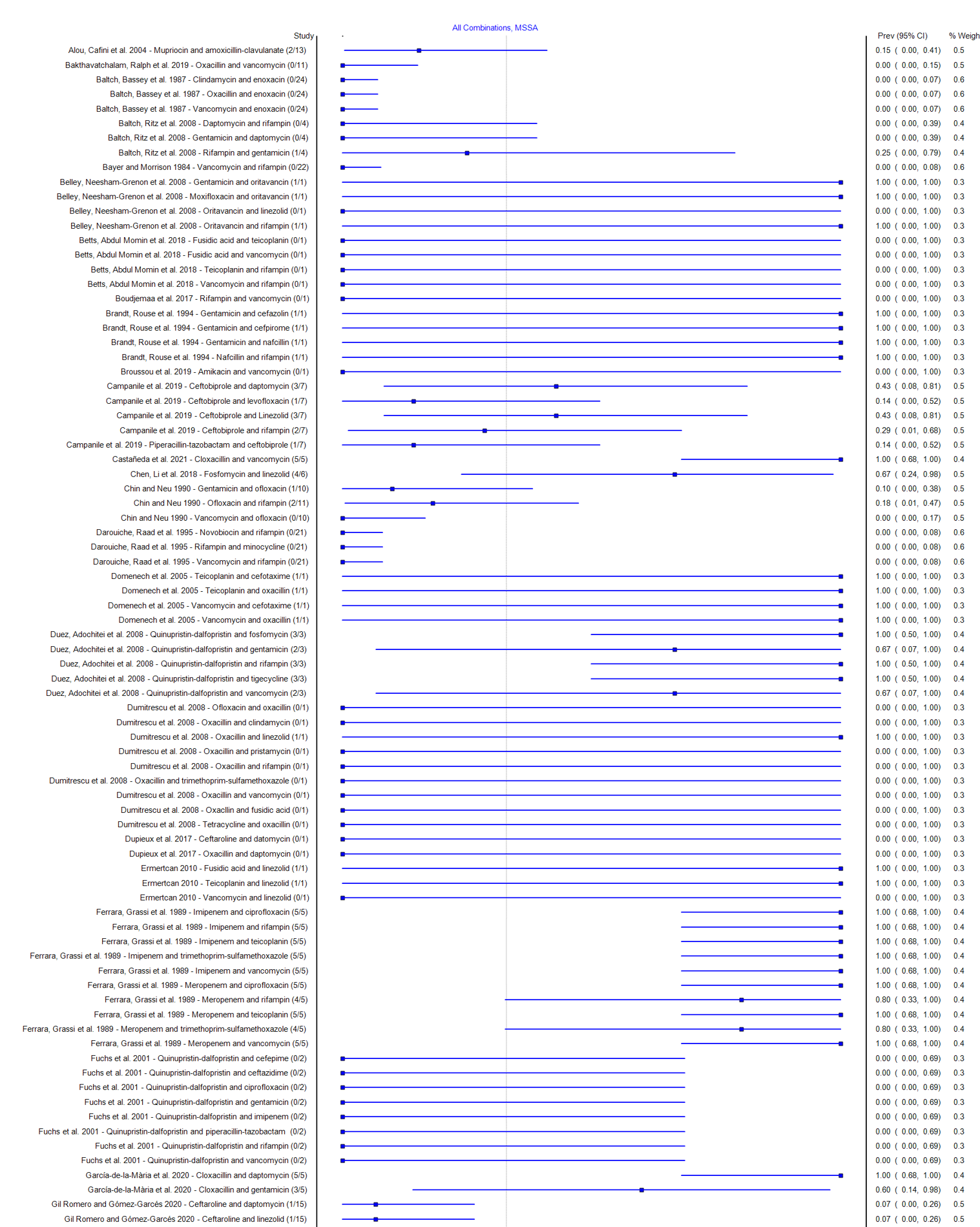


Figure continued on next page


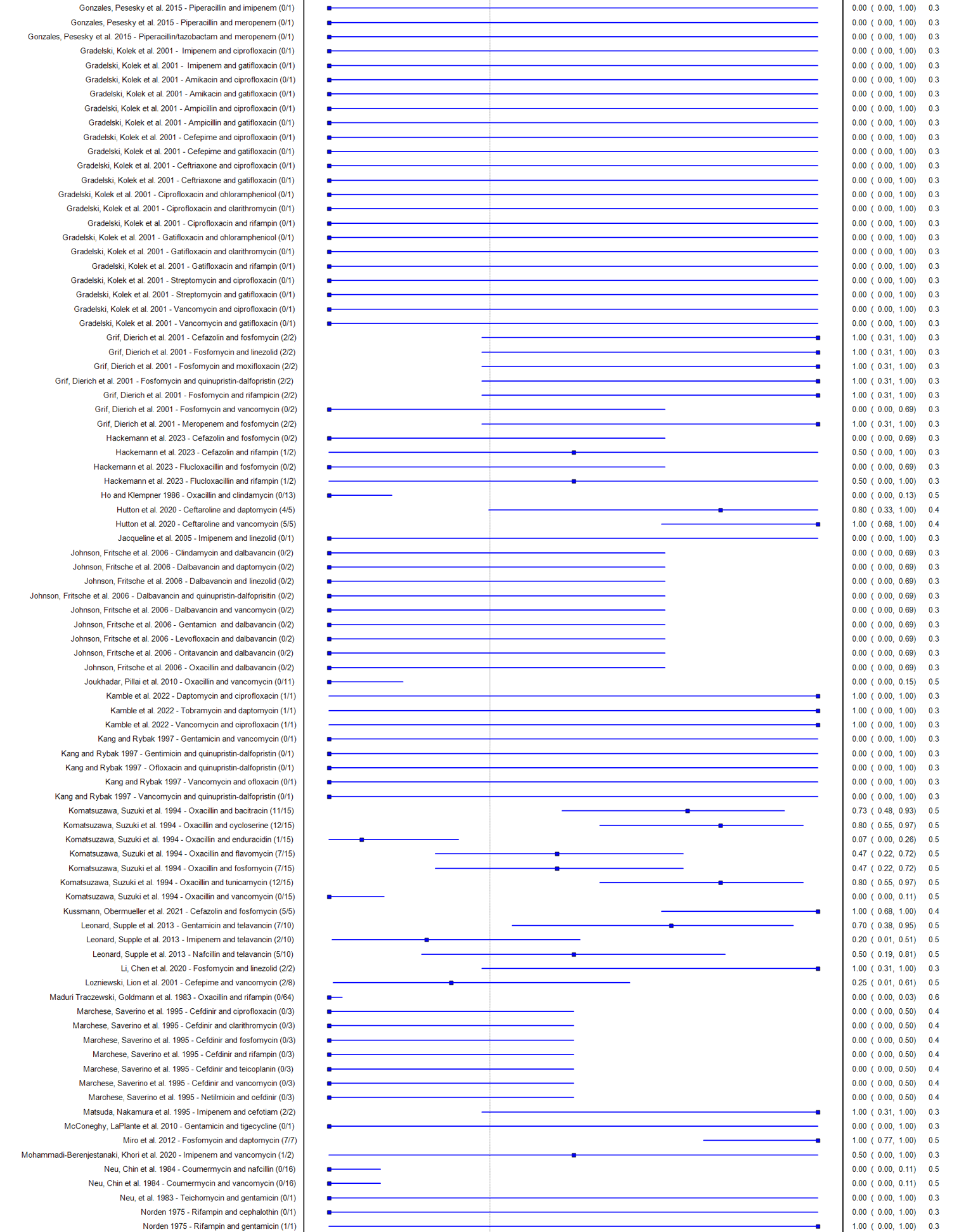


Figure continued on next page


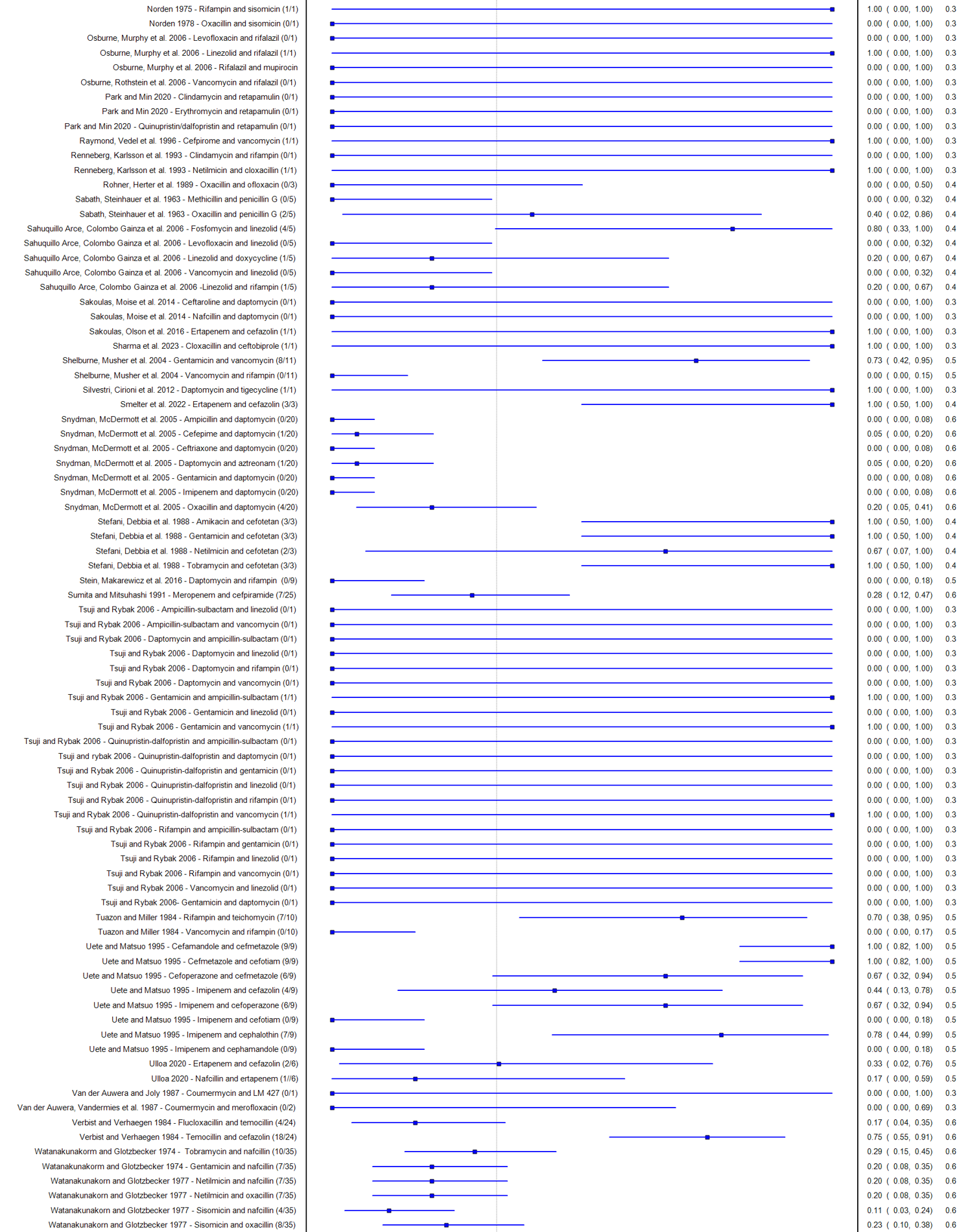


Figure continued on next page


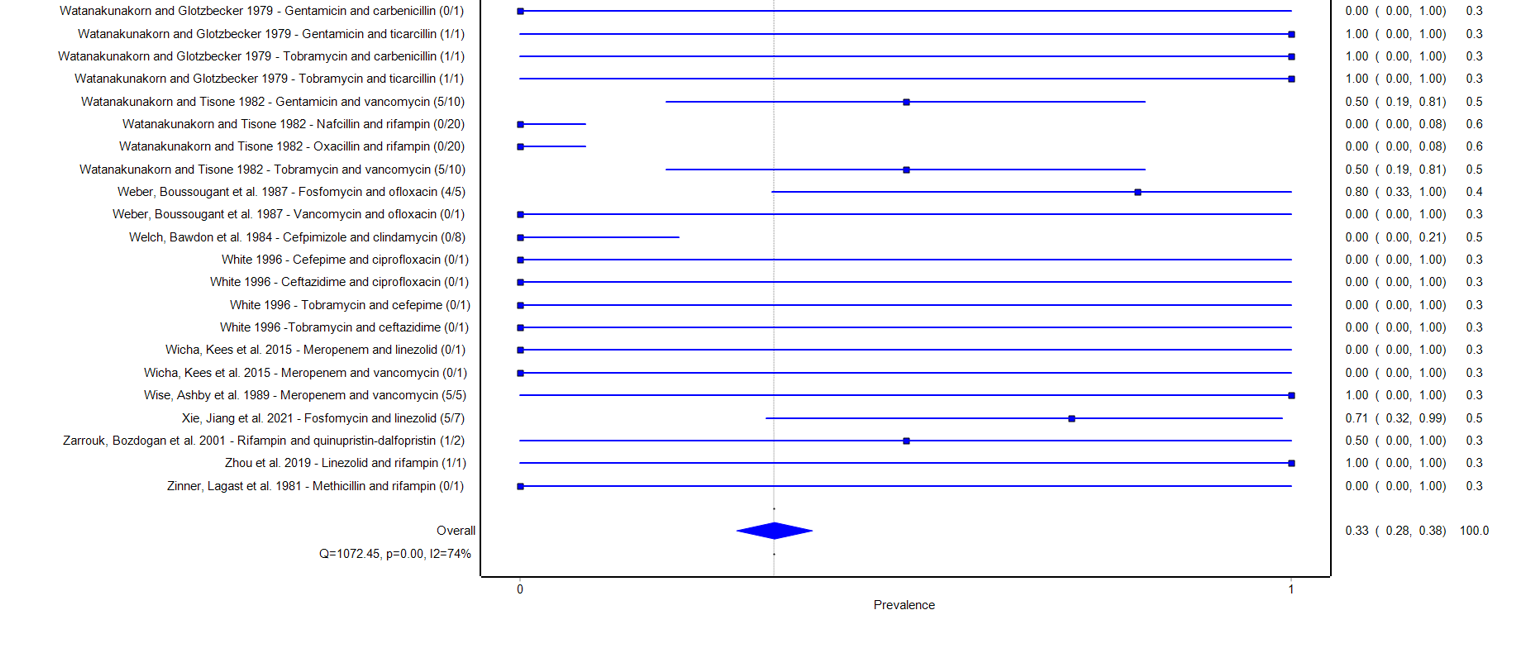


**Supplementary Figure 5.** Forrest plots for meta-analysis of proportions of all tested antimicrobial combinations for MSSA isolates.


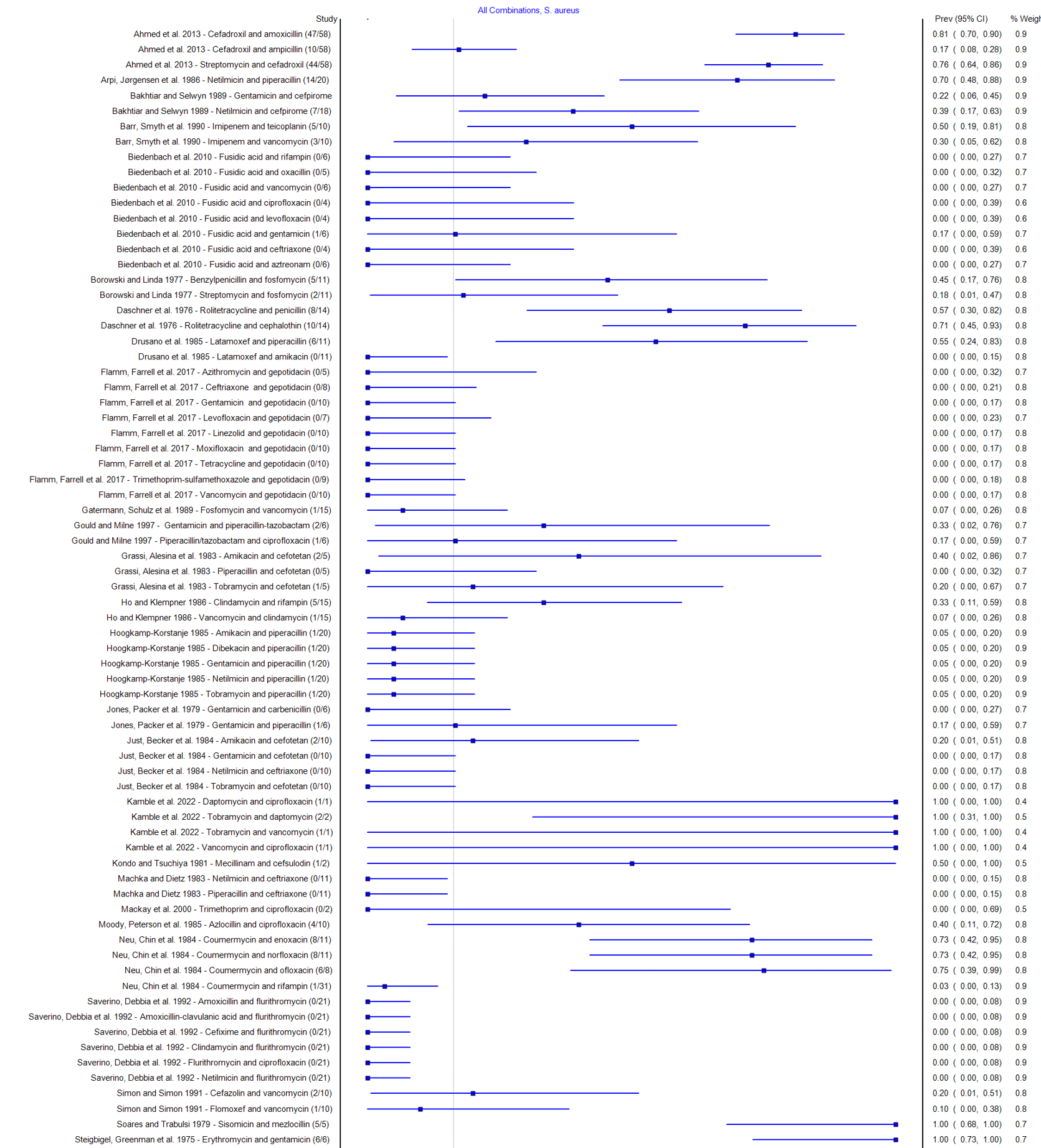


Figure continued on next page


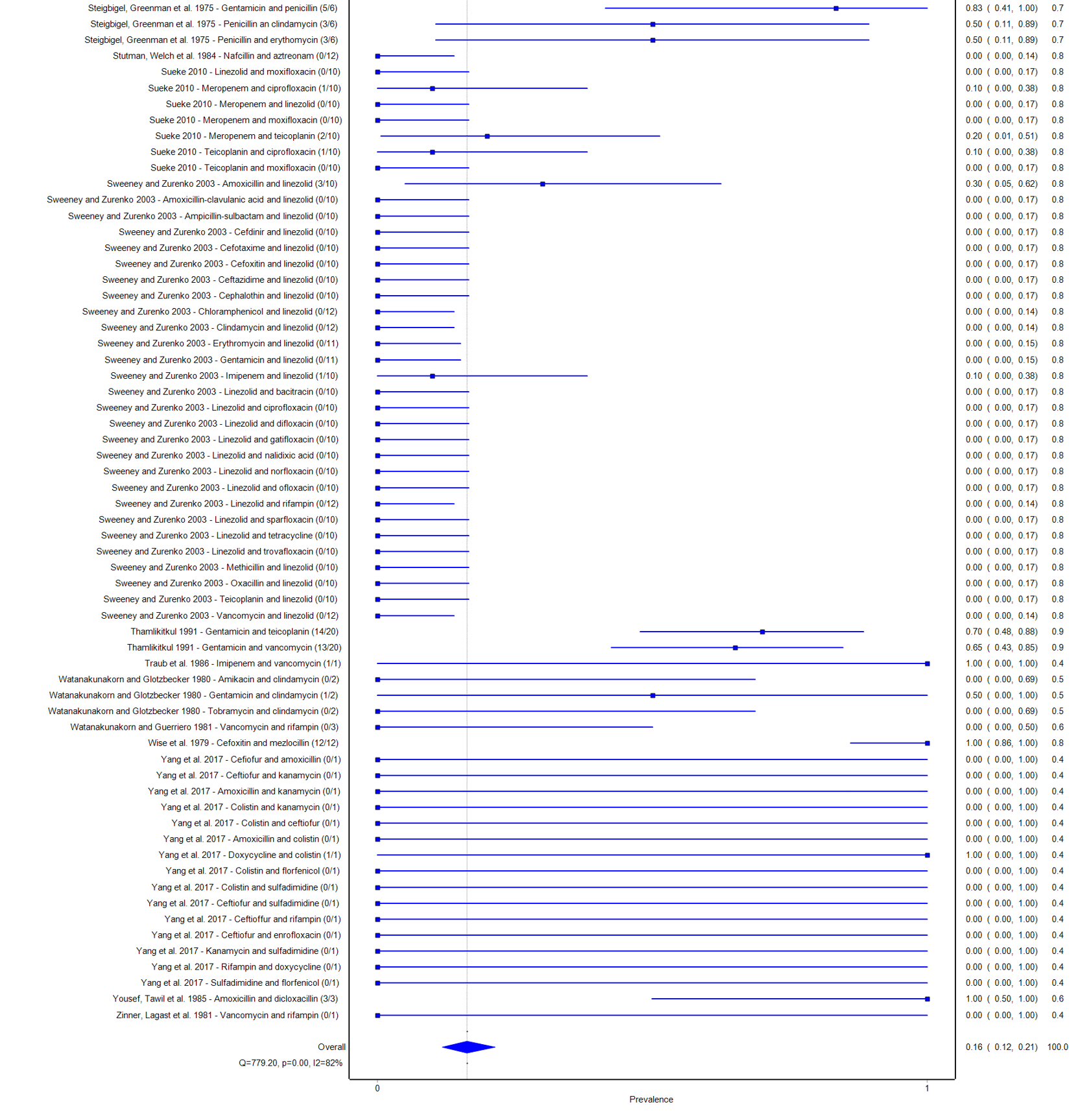


**Supplementary Figure 6.** Forrest plots for meta-analysis of proportions of all tested antimicrobial combinations for S. aureus isolates.
