# Supplementary material for: Antibiotic synergy against Staphylococcus aureus: a systematic review and meta-analysis
Source: Antimicrob Agents Chemother. 2025 Jun 17;69(8):e01199-24. doi: 10.1128/aac.01199-24 (PMC12326989; doi:10.1128/aac.01199-24)
Supplement: Supplementary methods and materials — Tables S1 to S3. [file aac.01199-24-s0002.docx]

**Antibiotic Synergy against Staphylococcus aureus: A Systematic Review**

**Supplementary Materials**

(((((combination) OR (synergy)) OR ((time kill assay) OR (in vitro))) AND (((antibiotic) OR (antimicrobial)) OR (antibacterial))) AND (((((("Streptococcus pyogenes") OR ("group A streptococcus")) OR ("staphylococcus aureus")) OR (staph aureus)) OR (MSSA)) OR (MRSA))) AND (((((((((((((penicillin) OR (vancomycin)) OR (linezolid)) OR (quinupristin)) OR (dalfopristin)) OR (daptomycin)) OR (nafcillin)) OR (oxacillin)) OR (cefazolin)) OR (clindamycin)) OR (gatifloxacin)) OR (tedizolid)) OR (ceftriaxone)).

**Supplementary Method 1:** Search strategy

PubMed was searched on September 28^th^, 2021, Web of Science Core Collection and Cochrane were searched on May 25^th^, 2022. PubMed, Web of Science and Cochrane were re-screened and de-duplicated on February 15^th^, 2024 to include recently published studies.

| **Class** | **Antibiotics** |
| --- | --- |
| Aminocoumarins | Coumermycin  Novobiocin |
| Aminoglycosides | Amikacin  Arbekacin  Azithromycin  Dibekacin  Gentamicin  Kanamycin  Netilmicin  Plazomicin (ACH-490)  Sisomicin  Streptomycin  Tobramycin |
| Beta-lactams | Amoxicillin  Amoxicillin-clavulanate  Ampicillin  Ampicillin-sulbactam  Azlocillin  Benzylpenicillin  Carbenicillin  Cloxacillin  Dicloxacillin  Flucloxacillin  Mezlocillin  Nafcillin  Oxacillin  Penicillin, penicillin-G  Piperacillin  Piperacillin-tazobactam  Temocillin  Ticarcillin-clavulanate |
| Carbapenems | Doripenem  Ertapenem  Imipenem  Meropenem  Panipenem |
| Cephalosporins | Cefadroxil  Cefamandole  Cefazolin  Cefazopran  Cefdinir  Cefepime  Cefixime  Cefmetazole  Cefoperazone  Cefotaxime  Cefotetan  Cefotiam  Cefoxitin  Cefpiramide  Cefpirome  Ceftaroline  Ceftazidime  Ceftiofur  Ceftobiprole  Ceftriaxone  Ceftrizoxime  Cefuroxime  Cefuzonam  Cephalexin  Cephaloridine  Cephalothin  Flomoxef  Latamoxef |
| Chloramphenicols | Chloramphenicol  Florfenicol |
| Cycloserines | D-cycloserine |
| Fosfomycin | Fosfomycin |
| Fusidic acid | Fusidic acid |
| Glycopeptides | Teichomycin  Teicoplanin  Vancomycin |
| Glycophospolipids | Flavomycin |
| Lincosamides | Clindamycin |
| Lipoglycopeptides | Dalbavancin  Oritavancin  Telavancin |
| Lipopeptides | Daptomycin |
| Macrolides | Clarithromycin  Erythromycin  Flurithromycin  Roxithromycin |
| Mupirocin | Mupirocin |
| Nucleosides | Gemcitabine |
| Oxazolidinones | Linezolid  Tedizolid |
| Pleuromutilins | Retapamulin |
| Polymixins | Colistin |
| Polypeptides | Bacitracin  Enduracidin  Enramycin |
| Quinolones | Balofloxacin  Ciprofloxacin  Difloxacin  Enoxacin  Enrofloxacin  Gatifloxacin  Gemifloxacin  Levofloxacin  Merafloxacin  Moxifloxacin  Nalidixic acid  Norfloxacin  Ofloxacin  Sitafloxacin  Sparfloxacin  Trovafloxacin |
| Rifamycins | Rifabutin (LM427)  Rifalazil  Rifamixin  Rifampin |
| Streptogramins | Pristamycin  Quinupristin-dalfopristin |
| Sulfonamides | Sulfadimidine  Trimethoprim  Trimethoprim-sulfamethoxazole (co-trimoxazole) |
| Tetracyclines | Doxycycline  Minocycline  Rolitetracycline |
| Topoisomerases | Gepotidacin |

**Supplementary Table 1.** Classification of included antibiotics

| **Reference** | **Isolates studied*** | **Antibiotics tested** | **Method used** |
| --- | --- | --- | --- |
| **Abdul-Mutakabbir, Kebriaei et al. 2020 (1)** | MRSA | Daptomycin and cefazolin, dalbavancin and cefazolin, vancomycin and cefazolin | TKA |
| **Aeschlimann et al. 2000 (2)** | MRSA | Gentamicin and vancomycin, gentamicin and oritavancin | Checkerboard and TKA |
| **Ahmad et al. 2010 (3)** | MRSA | Daptomycin and rifampin | TKA |
| **Ahmed et al. 2013 (4)** | *S. aureus** | Cefadroxil and amoxicillin, cefadroxil and ampicillin, cefadroxil and streptomycin | Checkerboard |
| **Aktas 2017 (5)** | MRSA | Ceftriaxone and linezolid, dalbavancin and ceftriaxone, daptomycin and ceftriaxone | Checkerboard |
| **Aktas 2021 (6)** | MRSA | Amikacin and vancomycin, azithromycin and vancomycin, ceftaroline and vancomycin, ceftobiprole and vancomycin, clindamycin and vancomycin, dalbavancin and vancomycin, daptomycin and vancomycin, linezolid and vancomycin, minocycline and vancomycin, quniupristin-dalfopristin and vancomycin, rifampin and vancomycin, telavancin and vancomycin, tigecycline and vancomycin, tobramycin and vancomycin, trimethoprim-sulfamethoxazole and vancomycin | E-test |
| **Aktas and Derbentli 2017 (7)** | MRSA | Daptomycin and fosfomycin, daptomycin and fusidic acid, daptomycin and gentamicin, daptomycin and rifampicin | Checkerboard |
| **Aktas and Derbentli 2017 (8)** | MRSA | Dalbavancin and linezolid, daptomycin and dalbavancin, daptomycin and linezolid | Checkerboard |
| **Alou, Cafini et al. 2004 (9)** | MSSA, MRSA | Mupriocin and amoxicillin-clavulanate | Checkerboard and TKA |
| **Arpi, Jørgensen et al. 1986 (10)** | *S. aureus* | Piperacillin and netilmicin | Checkerboard |
| **Azap et al. 2007 (11)** | MRSA | Vancomycin and imipenem, vancomycin and meropenem, vancomycin and cefepime, teicoplanin and imipenem, teicoplanin and meropenem, teicoplanin and cefepime | Checkerboard |
| **Bai et al. 2019 (12)** | MRSA | Oxacillin and oritavancin | Checkerboard |
| **Bakhtiar and Selwyn 1989 (13)** | *S. aureus* | Cefpirome and gentamicin, cefpirome and netilmicin | Checkerboard |
| **Bakthavatchalam, Ralph et al. 2019 (14)** | MSSA, MRSA | Oxacillin and vancomycin | Checkerboard and TKA |
| **Baldoni 2013 (15)** | MRSA | Rifampin and dalbavancin | TKA |
| **Baltch, Bassey et al. 1987 (16)** | MSSA | Enoxacin and clindamycin, enoxacin and oxacillin, enoxacin and vancomycin | Checkerboard |
| **Baltch, Ritz et al. 2007 (17)** | MSSA | Daptomycin and gentamicin, daptomycin and rifampin, rifampin and gentamicin; rifampin, daptomycin and gentamicin | TKA |
| **Baltch, Ritz et al. 2008 (18)** | MRSA | Daptomycin and gentamicin, daptomycin and rifampin, rifampin and gentamicin | TKA |
| **Banerjee, Fernandez et al. 2013 (19)** | MRSA | Cefazolin and cefoxitin, cefoxitin and nafcillin, cefuroxime and cefoxitin | TKA |
| **Barber, Werth et al. 2014 (20)** | MRSA | Daptomycin and ceftobiprole, gentamicin and ceftobiprole, rifampin and ceftobiprole | TKA |
| **Barr, Smyth et al. 1990 (21)** | *S. aureus* | Imipenem and vancomycin, imipenem and teicoplanin | Checkerboard and TKA |
| **Batard et al. 2002 (22)** | MRSA | Gentamicin and quinupristin-dalfopristin | Checkerboard and TKA |
| **Bayer and Lam 1985 (23)** | MRSA | Rifampin and vancomycin | Checkerboard and TKA |
| **Bayer and Morrison 1984 (24)** | MSSA, MRSA | Rifampin and vancomycin | Checkerboard and TKA |
| **Belley, Neesham-Grenon et al. 2008 (25)** | MSSA, MRSA | Gentamicin and oritavancin, linezolid and oritavancin, moxifloxacin and oritavancin, rifampin and oritavancin | TKA |
| **Bergeret and Raymond 1999 (26)** | MRSA | Vancomycin and cefamandole, vancomycin and cefpirome, teicoplanin and cefpirome | TKA |
| **Bergeret, Boutros et al. 2004 (27)** | MRSA | Cefprirome and teicoplanin, cefprirome and vancomycin | TKA |
| **Betts, Abdul Momin et al. 2018 (28)** | MSSA, MRSA | Fusidic acid and teicoplanin, fusidic acid and vancomycin, rifampin and vancomycin, rifampin and teicoplanin | Checkerboard and TKA |
| **Biedenbach et al. 2010 (29)** | *S. aureus* | Fusidic acid and rifampin, fusidic acid and ciprofloxacin, fusidic acid and levofloxacin, fusidic acid and gentamicin, fusidic acid and oxacillin, fusidic acid and ceftriaxone, fusidic acid and vancomycin, fusidic acid and aztreonam | Checkerboard and TKA |
| **Bishr et. al, 2021 (30)** | MRSA | Azithromycin and amikacin, azithromycin and cefotaxime, azithromycin and ceftriaxone, azithromycin and gentamicin, azithromycin and linezolid | Checkerboard |
| **Borowski and Linda 1977 (31)** | *S. aureus* | Fosfomycin and benzylpenicillin, fosfomycin and streptomycin | Checkerboard |
| **Boudjemaa et al. 2017 (32)** | MSSA | Vancomycin and rifampin | TKA |
| **Boudrioua et al. 2020 (33)** | MRSA | D-cycloserine and vancomycin | Checkerboard |
| **Brandt, Rouse et al. 1994 (34)** | MSSA | Gentamicin and cefazolin, gentamicin and cefpirome, gentamicin and nafcillin, rifampin and nafcillin | TKA |
| **Broussou et al. 2019 (35)** | MSSA, MRSA | Vancomycin and amikacin | Checkerboard and TKA |
| **Bulger 1967 (36)** | MRSA | Cephalothin and kanamycin | TKA |
| **Cabellos et al. 2014 (37)** | MRSA | Vancomycin and linezolid, rifampin and linezolid | TKA |
| **Campanile et al. 2019 (38)** | MSSA, MRSA | Ceftobiprole and daptomycin, ceftobiprole and levofloxacin, ceftobiprole and linezolid, ceftobiprole and piperacillin-tazobactam, ceftobiprole and rifampin | E-test, TKA |
| **Carricajo et al. 2001 (39)** | MRSA | Cefpirome and vancomycin | TKA |
| **Castañeda 2021 (40)** | MSSA, MRSA | Vancomycin and cloxacillin | TKA |
| **Chai, Liu et al. 2016 (41)** | MRSA | Fosfomycin and linezolid, levofloxacin and linezolid, rifampin and linezolid | Checkerboard and TKA |
| **Chang, Hsieh et al. 1989 (42)** | MRSA | Fosfomycin and cefazolin, fosfomycin and cefmetazole, vancomycin and cefamandole, vancomycin and cefazolin, vancomycin and gentamicin, vancomycin and imipenem, vancomycin and rifampin | Checkerboard |
| **Chen, Li et al. 2018 (43)** | MSSA, MRSA | Fosfomycin and linezolid | Checkerboard and TKA |
| **Chen, Takahashi 1988 (44)** | MRSA | Cefamandole and vancomycin, fosfomycin and cefamandole, fosfomycin and vancomycin, minocycline and cefamandole, minocycline and vancomycin | Checkerboard |
| **Chin and Neu 1990 (45)** | MSSA, MRSA | Rifampin and ofloxacin, gentamicin and ofloxacin, vancomycin and ofloxacin | Checkerboard |
| **Chin, Neu et al. 1986 (46)** | MRSA | Azlocillin and ciprofloxacin | Checkerboard |
| **Choi, Moon et al. 2020 (47)** | MRSA | Colistin and vancomycin | Checkerboard and TKA |
| **Cilli, Aydemir et al. 2006 (48)** | MRSA | Daptomycin and ampicillin-sulbactam, daptomycin and piperacillin-tazobactam, daptomycin and ticarcillin-clavulanate | E-test |
| **Claeys, Smith et al. 2015 (49)** | MRSA | Trimethoprim-sulfamethoxazole and daptomycin | TKA |
| **Climo, Patron et al. 1999 (50)** | MRSA | Vancomycin and oxacillin | Checkerboard and TKA |
| **Coban 2010 (51)** | MRSA | Daptomycin and teicoplanin, daptomycin and tigecycline, teicoplanin and tigecycline | E-test |
| **Credito, Lin et al. 2007 (52)** | MRSA | Daptomycin and gentamicin | TKA |
| **D'Arezzo, Mazzarelli et al. 2017 (53)** | MRSA | Ampicillin and ceftaroline | E-test |
| **Darouiche, Raad et al. 1995 (54)** | MSSA, MRSA | Rifampin and minocycline, rifampin and novobiocin, rifampin and vancomycin | TKA |
| **Daschner et al. 1976 (55)** | *S. aureus* | Rolitetracycline and penicillin, rolitetracycline and cephalothin | Checkerboard |
| **Dawis, Isenberg et al. 2003 (56)** | MRSA | Gatifloxacin and cefepime, gatifloxacin and gentamicin, gatifloxacin and meropenem, gatifloxacin and piperacillin | Checkerboard |
| **Del rio et al. 2016 (57)** | MRSA | Fosfomycin and amoxicillin-clavulanic acid, fosfomycin and ceftriaxone, fosfomycin and imipenem | TKA |
| **Dhand and Bayer 2011 (58)** | MRSA | Daptomycin and oxacillin | TKA |
| **Dilworth, Sanchez et al. 2019 (59)** | MRSA | Vancomycin and piperacillin, vancomycin and piperacillin-tazobactam | TKA |
| **Dilworth, Sliwinski et al. 2014 (60)** | MRSA | Vancomycin and oxacillin, vancomycin and piperacillin-tazobactam | TKA |
| **Dixson et al. 1985 (61)** | MRSA | Novobiocin and rifampin, teicoplanin and rifampin, vancomycin and rifampin | Checkerboard and TKA |
| **Domaracki, Evans et al. 2000 (62)** | MRSA | Vancomycin and oxacillin | Checkerboard and TKA |
| **Domenech et al. 2005 (63)** | MSSA, MRSA | Vancomycin and oxacillin, vancomycin and cefotaxime, teicoplanin and oxacillin, teicoplanin and cefotaxime | TKA |
| **Drago, De Vecchi et al. 2007 (64)** | MRSA | Vancomycin and piperacillin/tazobactam, teicoplanin and cefotaxime, teicoplanin and levofloxaci, vancomycin and cefotaxime, vancomycin and levofloxacin | Checkerboard and TKA |
| **Drusano et al. 1985 (65)** | *S. aureus* | Latamoxef and piperacillin, latamoxef and amikacin | Checkerboard |
| **Duez, Adochitei et al. 2008 (66)** | MSSA, MRSA | Vancomycin and piperacillin/tazobactam, teicoplanin and cefotaxime, teicoplanin and levofloxaci, vancomycin and cefotaxime, vancomycin and levofloxacin | Checkerboard |
| **Dumitrescu et al. 2008 (67)** | MSSA | Oxacillin and vancomycin, oxacillin and ofloxacin, oxacillin and co-trimoxazole, oxacillin and pristamycin, oxacillin and clindamycin, oxacillin and rifampin, oxacillin and fusidic acid, oxacillin and tetracycline, oxacillin and fusidic acid, oxacillin and tetracycline, oxacillin and linezolid | Checkerboard |
| **Dupieux et al. 2017 (68)** | MSSA, MRSA | Daptomycin and oxacillin, daptomycin and ceftaroline | Checkerboard |
| **Entenza et al. 2011 (69)** | MRSA | Vancomycin and ceftobiprole | TKA |
| **Ermertcan 2010 (70)** | MSSA, MRSA | Linezolid and fusidic acid, linezolid and vancomycin, linezolid and teicoplanin | TKA |
| **Fantin 1993 (71)** | MRSA | Vancomycin and fusidic acid | TKA |
| **Fern et al. 2012 (72)** | MRSA | Ceftobiprole and vancomycin | TKA and checkerboard |
| **Ferrara, Dos Santos et al. 1997 (73)** | MRSA | Sparfloxacin and oxacillin, fosfomycin and oxacillin, fosfomycin and sparfloxacin | TKA |
| **Ferrara, Grassi et al. 1989 (74)** | MSSA, MRSA | Meropenem and ciprofloxacin, imipenem and ciprofloxacin, meropenem and rifampin, imipenem and rifampin, meropenem and teicoplanin, imipenem and teicoplanin, meropenem and vancomycin, imipenem and vancomycin, meropenem and co-trimoxazole, imipenem and co-trimoxazole, meropenem and netilmicin, imipenem and netilmicin | Checkerboard |
| **Flamm, Farrell et al. 2017 (75)** | *S. aureus* | Gepotidacin and azithromycin, gepotidacin and aztreonam, gepotidacin and ceftriaxone, gepotidacin and gentamicin, gepotidacin and levofloxacin, gepotidacin and linezolid, gepotidacin and moxifloxacin, gepotidacin and tetracycline, gepotidacin and trimethoprim-sulfamethoxazole, gepotidacin and vancomycin | Checkerboard |
| **Fuchs et al. 2001 (76)** | MSSA, MRSA | Quinupristin-dalfopristin and vancomycin, quinupristin-dalfopristin and cefepime, quinupristin-dalfopristin and ceftazidime, quinupristin-dalfopristin and imipenem, quinupristin-dalfopristin and piperacillin-tazobactam, quinupristin-dalfopristin and ciprofloxacin, quinupristin-dalfopristin and gentamicin, quinupristin-dalfopristin and rifampin | TKA |
| **García et al. 2016 (77)** | MRSA | Daptomycin and ceftaroline, linezolid and ceftaroline, vancomycin and ceftaroline | Checkerboard |
| **García-de-la-Mària et al. 2018 (78)** | MRSA | Daptomycin and fosfomycin, daptomycin and cloxacillin | TKA |
| **García-de-la-Mària et al. 2020 (79)** | MSSA | Daptomycin and cloxacillin, gentamicin and cloxacillin | TKA |
| **Garrigos et al. 2012 (80)** | MRSA | Daptomycin and cloxacillin | TKA |
| **Gatermann, Schulz et al. 1989 (81)** | *S. aureus* | Fosfomycin and vancomycin | Checkerboard |
| **Gil Romero and Gómez-Garcés 2020 (82)** | MSSA, MRSA | Daptomycin and ceftaroline, linezolid and ceftaroline, vancomycin and ceftaroline | E-test |
| **Gonzales, Pesesky et al. 2015 (83)** | MSSA, MRSA | Aztreonam and piperacillin-tazobactam, cefepime and piperacillin-tazobactam, imipenem and piperacillin, meropenem and piperacillin, meropenem and piperacillin-tazobactam | Checkerboard |
| **Goto 1999 (84)** | MRSA | Minocycline and netilmicin | Checkerboard |
| **Gould and Milne 1997 (85)** | *S. aureus* | Ciprofloxacin and piperacillin-tazobactam, gentamicin and piperacillin-tazobactam | Checkerboard and TKA |
| **Gradelski, Kolek et al. 2001 (86)** | MSSA | Amikacin and ciprofloxacin, amikacin and gatifloxacin, cefepime and ciprofloxacin, cefepime and gatifloxacin, ceftriaxone and ciprofloxacin, ceftriaxone and gatifloxacin, ciprofloxacin and ampicillin, ciprofloxacin and chloramphenicol, ciprofloxacin and vancomycin, clarithromycin and ciprofloxacin, gatifloxacin and ampicillin, gatifloxacin and chloramphenicol, gatifloxacin and clarithromycin, gatifloxacin and vancomycin, imipenem and ciprofloxacin, imipenem and gatifloxacin, rifampin and ciprofloxacin, rifampin and gatifloxacin, streptomycin and ciprofloxacin, streptomycin and gatifloxacin | Checkerboard and TKA |
| **Grassi, Alesina et al. 1983 (87)** | *S. aureus* | Cefotetan and amikacin, cefotetan and piperacillin, cefotetan and tobramycin | Checkerboard and TKA |
| **Grif, Dierich et al. 2001 (88)** | MSSA, MRSA | Fosfomycin and cefazolin, fosfomycin and linezolid, fosfomycin and meropenem, fosfomycin and moxifloxacin, fosfomycin and quinupristin-dalfopristin, fosfomycin and rifampin, fosfomycin and vancomycin | Checkerboard and TKA |
| **Hackemann et al. 2023 (89)** | MSSA | Cefazolin and rifampin, flucloxacillin and rifampin, flucloxacillin and fosfomycin, cefazolin and fosfomycin | Checkerboard |
| **Henson 2017 (90)** | MRSA | Daptomycin and ampicillin, daptomycin and ampicillin-sulbactam, daptomycin and piperacillin, daptomycin and piperacillin-tazobactam | TKA |
| **Hershberger et al. 1999 (91)** | MRSA | Vancomycin and gentamicin, teicoplanin and gentamicin, oritavancin and gentamicin | TKA |
| **Ho and Klempner 1986 (92)** | MSSA, MRSA, *S. aureus* | Oxacillin and clindamycin, vancomycin and clindamycin, vancomycin and rifampin | TKA |
| **Hoogkamp-Korstanje 1985 (93)** | *S. aureus* | Piperacillin and amikacin, piperacillin and dibekacin, piperacillin and gentamicin, piperacillin and netilmicin, piperacillin and tobramycin | Checkerboard |
| **Hosgor-Limoncu, Ermertcan et al. 2008 (94)** | MRSA | Amikacin and ciprofloxacin, amikacin and ertapenem, amikacin and levofloxacin, ciprofloxacin and ertapenem | TKA |
| **Howe et al. 1999 (95)** | MRSA | Vancomycin and methicillin | Checkerboard and E-test |
| **Hutton et al. 2020 (96)** | MSSA, MRSA | Daptomycin and ceftaroline, vancomycin and ceftaroline | TKA |
| **Jacqueline, Caillon et al. 2003 (97)** | MRSA | Linezolid and gentamicin, linezolid and rifampicin, linezolid and vancomycin | TKA |
| **Jacqueline et al. 2005 (98)** | MSSA, MRSA | Linezolid and imipenem | Checkerboard and TKA |
| **Jacqueline et al. 2006 (99)** | MRSA | Linezolid and ertapenem | Checkerboard and TKA |
| **Jiang 2015 (100)** | MRSA | Daptomycin and gentamicin | TKA |
| **Johnson, Fritsche et al. 2006 (101)** | MSSA, MRSA | Clindamycin and dalbavancin, dalbavancin and vancomycin, daptomycin and dalbavancin, gentamicin and dalbavancin, levofloxacin and dalbavancin, linezolid and dalbavancin, oxacillin and dalbavancin, quinupristin-dalfopristin and dalbavancin, rifampin and dalbavancin | Checkerboard |
| **Johnston et al. 1987 (102)** | MRSA | Novobiocin and rifampin | TKA and checkerboard |
| **Jones, Packer et al. 1979 (103)** | *S. aureus* | Piperacillin and gentamicin, carbenicillin and gentamicin | Checkerboard |
| **Joukhadar, Pillai et al. 2010 (104)** | MSSA | Oxacillin and vancomycin | TKA |
| **Just, Becker et al. 1984 (105)** | *S. aureus* | Cefotetan and amikacin, cefotetan and gentamicin, cefotetan and netilmicin, cefotetan and tobramycin | Checkerboard |
| **Kamble et al. 2022 (106)** | MSSA, *S. aureus* | Ciprofloxacin and vancomycin, ciprofloxacin and daptomycin, tobramycin and daptomycin, tobramycin and vancomycin | Checkerboard and TKA |
| **Kang et al. 2016 (107)** | MRSA | Vancomycin and ciprofloxacin, vancomycin and gentamicin, vancomycin and trimethoprim-sulfamethoxazole, vancomycin and rifampin, vancomycin and clindamycin, ciprofloxacin and clindamycin, ciprofloxacin and trimethoprim-sulfamethoxazole, ciprofloxacin and rifampin, rifampin and clindamycin, rifampin and trimethoprim-sulfamethoxazole, clindamycin trimethoprim-sulfamethoxazole | TKA |
| **Kang and Rybak 1997 (108)** | MSSA, MRSA | Gentamicin and vancomycin, ofloxacin and vancomycin, quinupristin-dalfopristin and vancomycin, quinupristin-dalfopristin and gentamicin, quinupristin-dalfopristin and ofloxacin | Checkerboard and TKA |
| **Katou et al. 2005 (109)** | MRSA | Panipenem and arbekacin, panipenem and amikacin, panipenem and netilmicin, panipenem and vancomycin | Checkerboard |
| **Kebriaei, Rice et. al 2020 (110)** | MRSA | Dalbavancin and cefazolin, dalbavancin and cefepime, dalbavancin and ceftaroline, oritavancin and cefazolin, oritavancin and cefepime, oritavancin and ceftaroline, oxacillin and dalbavancin, oxacillin and oritavancin, teicoplanin and cefazolin, teicoplanin and cefepime, teicoplanin and ceftaroline, teicoplanin and oxacillin, telavancin and cefazolin, telavancin and cefepime, telavancin and ceftaroline, telavancin and oxacillin, vancomycin and cefazolin, vancomycin and cefepime, vancomycin and ceftaroline, vancomycin and oxacillin | TKA |
| **Khasawneh et al. 2008 (111)** | MRSA | Daptomycin and rifampin | TKA |
| **Kobayashi 2005 (112)** | MRSA | Doripenem and teicoplanin, doripenem and vancomycin, imipenem and vancomycin, imipenem and teicoplanin, meropenem and vancomycin, meropenem and teicoplanin, panipenem and vancomycin, panipenem and teicoplanin | Checkerboard |
| **Kobayashi, Arai et al. 1989 (113)** | MRSA | Cefazolin and rolitetracycline, cefmetazole and rolitetracycline, cefotaxime and rolitetracycline, ceftrizoxime and rolitetracycline, latamoxef and rolitetracycline, methicillin and rolitetracycline, | Checkerboard and TKA |
| **Komatsuzawa, Suzuki et al. 1994 (114)** | MSSA, MRSA | Oxacillin and bacitracin, oxacillin and cycloserine, oxacillin and enduracidin, oxacillin and flavomycin, oxacillin and fosfomycin, oxacillin and tunicamycin, oxacillin and vancomycin | Checkerboard |
| **Kondo and Tsuchiya 1981 (115)** | *S. aureus* | Mecillinam and cefsulodin | Checkerboard and TKA |
| **Kussmann, Obermueller et al. 2021 (116)** | MSSA, MRSA | Fosfomycin and cefazolin | Checkerboard |
| **Lai et al. 2017 (117)** | MRSA | Vancomycin and cephalothin, vancomycin and cefmetazole, vancomycin and cefotaxime, vancomycin and cefpirome, teicoplanin and cephalothin, teicoplanin and cefmetazole, teicoplanin and cefotaxime, teicoplanin and cefpirome | TKA |
| **Lai et al. 2019 (118)** | MRSA | Daptomycin and cefazolin, daptomycin and cefmetazole, daptomycin and cefotaxime, daptomycin and cefepime | Checkerboard and TKA |
| **Lee 2013 (119)** | MRSA | Spray-dried ciprofloxazin and gatifloxacin | TKA |
| **Lee, Chen et al. 2019 (120)** | MRSA | Daptomycin and fosfomycin, daptomycin and gentamicin, daptomycin and linezolid, daptomycin and oxacillin, daptomycin and rifampin. | Checkerboard |
| **Lee et al. 2006 (121)** | MRSA | Vancomycin and arbekacin, rifampin and arbekacin, ampicillin-sulbactam and arbekacin, teicoplanin and arbekacin, quinupristin-dalfopristin and arbekacin | TKA |
| **Leonard 2012 (122)** | MRSA | Vancomycin and Nafcillin | TKA |
| **Leonard and rolek 2013 (123)** | MRSA | Daptomycin and nafcillin | TKA |
| **Leonard, Cheung et al. 2008 (124)** | MRSA | Ceftobiprole and tobramycin | TKA |
| **Leonard, Kaatz et al. 2008 (125)** | MRSA | Clindamycin and gemifloxacin, rifampin and gemifloxacin, trimethoprim-sulfamethoxazole and gemifloxacin | TKA |
| **Leonard, Supple et al. 2013 (126)** | MSSA, MRSA | Telavancin and gentamicin, telavancin and imipenem, telavancin and nafcillin | TKA |
| **Li, Chen et al. 2020 (127)** | MSSA, MRSA | Fosfomycin and linezolid | Checkerboard and TKA |
| **Liao, Chen et al. 2017 (128)** | MRSA | Vancomycin and cefazolin, vancomycin and cefepime, vancomycin and cefoxitin, vancomycin and ceftazidime, vancomycin and ceftriaxone, vancomycin and oxacillin, vancomycin and penicillin | Checkerboard |
| **Lin et al. 2014 (129)** | MRSA | Oritavancin and gentamicin, oritavancin and linezolid, oritavancin and rifampin | TKA |
| **Lin, Ednie et al. 2010 (130)** | MRSA | ACHN-490 (plasomicin) and daptomycin | TKA |
| **Lin, Pankuch et al. 2010 (131)** | MRSA | Telavancin and ceftriaxone, Telavancin and gentamicin, Telavancin and meropenem, Telavancin and rifampin | TKA |
| **Liu 2016 (132)** | MRSA | Erythromycin and oxacillin | Checkerboard |
| **Lozniewski, Lion et al. 2001 (133)** | MSSA, MRSA | Vancomycin and cefepime | Checkerboard and TKA |
| **Machka and Dietz 1983 (134)** | *S. aureus* | Ceftriaxone and piperacillin, ceftriaxone and netilmicin | Checkerboard |
| **Mackay et al. 2000 (135)** | *S. aureus* | Trimethoprim and ciprofloxacin | Checkerboard and TKA |
| **Maduri Traczewski, Goldmann et al. 1983 (136)** | *S. aureus* | Rifampin and oxacillin | Checkerboard and TKA |
| **Marchese, Saverino et al. 1995 (137)** | MSSA | Ciprofloxacin and cefdinir, clarithromycin and cefdinir, fosfomycin and cefdinir, netilmicin and cefdinir, rifampin and cefdinir, teicoplanin and cefdinir, vancomycin and cefdinir | Checkerboard and TKA |
| **Matsuda, Nakamura et al. 1995 (138)** | MSSA, MRSA | Imipenem and cefotiam | Checkerboard |
| **Matsumoto et al. 1993 (139)** | MRSA | Cefuzonam and minocycline, cefuzonam and fosfomycin | Checkerboard |
| **McConeghy, K. W. and K. L. LaPlante 2010 (140)** | MSSA, MRSA | Tigecycline and gentamicin | Checkerboard and TKA |
| **Mehta 2012 (141)** | MRSA | Amoxicillin-clavulanic acid and daptomycin, cefotaxime and daptomycin, imipenem and daptomycin, oxacillin and daptomycin | Checkerboard and TKA |
| **Mercier, Houlihan et al. 1997 (142)** | MRSA | Oritavancin and gentamicin, oritavancin and rifampin, oritavancin and vancomycin, vancomycin and gentamicin | TKA |
| **Mercier, Kennedy et al. 2002 (143)** | MRSA | Doxycycline and tetracycline, doxycycline and vancomycin, gentamicin and tigecycline, gentimicin and vancomycin, rifampin and tigecycline, rifampin and vancomycin, tigecycline and vancomycin | TKA |
| **Meyers, Srulevitch et al. 1985 (144)** | MRSA | Coumermycin and cephalothin, coumermycin and ciprofloxacin, coumermycin and gentamicin, coumermycin and vancomycin | Checkerboard |
| **Miranda-Novalez et al. 2006 (145)** | MRSA | Dicloxacillin and amikacin, cephalthin and amikacin, cephalothin and vancomycin, imipenem and vancomycin, vancomycin and amikacin. | Checkerboard |
| **Miró, García-de-la-Mària et al. 2009 (146)** | MRSA | Daptomycin and gentamicin, daptomycin and rifampin | TKA |
| **Miró et al. 2012 (147)** | MSSA, MRSA | Fosfomycin and daptomycin | TKA |
| **Mirza and Oguc Sanli 2024 (148)** | MRSA | Trimethoprim-sulfamethoxazole and ceftaroline, Trimethoprim-sulfamethoxaole and ceftobiprole | E-test |
| **Mohammadi-Berenjestanaki, Khori et al. 2020 (149)** | MSSA, MRSA | Imipenem and vancomycin | Checkerboard and TKA |
| **Moody, Peterson et al. 1985 (150)** | *S. aureus* | Azlocillin and ciprofloxacin | Checkerboard |
| **Mulazimoglu, Drenning et al. 1996 (151)** | MRSA | Gentamicin and vancomycin | TKA |
| **Mulazimoglu et al. 1996 (152)** | MRSA | Linezolid and vancomycin, linezolid and rifampin | TKA |
| **Neu, Chin et al. 1984 (153)** | MSSA, MRSA, *S. aureus* | Coumermycin and enoxacin, Coumermycin and nafcillin, Coumermycin and norfloxacin, Coumermycin and ofloxacin, Coumermycin and rifampin, Coumermycin and vancomycin | Checkerboard |
| **Neu et al. 1983 (154)** | MSSA, MRSA | Gentamicin and teichomycin | Checkerboard and TKA |
| **Norden 1975 (155)** | MSSA | Rifampin and gentamicin, rifampin and sisomicin, rifampin and cephalothin | Checkerboard and TKA |
| **Norden 1978 (156)** | MSSA | Oxacillin and sisomicin | Checkerboard and TKA |
| **Oka 1993 (157)** | MRSA | Cefotiam and imipenem | Checkerboard |
| **Osburne, Murphy et al. 2006 (158)** | MSSA | Rifalazil and levofloxacin, rifalazil and linezolid, rifalazil and muriprocin | Checkerboard and TKA |
| **Osburne, Rothstein et al. 2006 (159)** | MSSA | Rifalazil and vancomycin | Checkerboard and TKA |
| **Pachón-Ibáñez et al. 2011 (160)** | MRSA | Fosfomycin and linezolid, fosfomycin and vancomycin, fosfomycin and imipenem | TKA |
| **Palmer and Rybak 1997 (161)** | MRSA | Vancomycin and ampicillin-sulbactam, vancomycin and imipenem, vancomycin and nafcillin, vancomycin and piperacillin-tazobactam | Checkerboard and TKA |
| **Park and Min 2020 (162)** | MSSA | Clindamycin and retapamulin, erythromycin and retapamulin, quinupristin-dalfopristin and retapamulin | Checkerboard |
| **Patel 1993 (163)** | MRSA | Levofloxacin and oxacillin | TKA |
| **Pavie et al. 2002 (164)** | MRSA | Quinupristin-dalfopristin and vancomycin | TKA |
| **Petersen 2006 (165)** | MRSA | Rifampicin and tigecycline, Vancomycin and tigecycline | Checkerboard and TKA |
| **Rand and Houck 2004 (166)** | MRSA | Daptomycin and oxacillin | TKA |
| **Rand et al. 1995 (167)** | MRSA | Imipenem and cefoperazone | Checkerboard |
| **Raymond, Vedel et al. 1998 (168)** | MSSA, MRSA | Vancomycin and cefpirome | TKA |
| **Renneberg, Karlsson et al. 1993 (169)** | MSSA, MRSA | Cloxacillin and netilmicin, clindamycin and rifampin | Checkerboard |
| **Ribes et al. 2010 (170)** | MRSA | Vancomycin and linezolid, vancomycin and imipenem | TKA |
| **Rochon-Edouard, Pestel-Caron et al. 2000 (171)** | MRSA | Netilmicin and cefazolin, netilmicin and imipenem, netilmicin and vancomycin, vancomycin and cefazolin, vancomycin and imipenem | Checkerboard and TKA |
| **Rohner, Herter et al. 1989 (172)** | MSSA, MRSA | Oxacillin and ofloxacin | Checkerboard and TKA |
| **Rose, Berti et al. 2013 (173)** | MRSA | Daptomycin and Rifampin | Checkerboard and TKA |
| **Rose et al. 2009 (174)** | MRSA | Vancomycin and tigecycline, vancomycin and rifampin | TKA |
| **Sabath, Steinhauer et al. 1963 (175)** | MSSA | Methicillin and penicillin G, Oxacillin and penicillin G | Checkerboard |
| **Sahuquillo Arce, Colombo Gainza et al. 2006 (176)** | MSSA | Doxycycline and linezolid, fosfomycin and linezolid, rifampin and linezolid, vancomycin and linezolid, levofloxacin and linezolid | Checkerboard and TKA |
| **Sakoulas, Moise et al. 2014 (177)** | MSSA, MRSA | Daptomycin and ceftaroline, daptomycin and nafcillin | Checkerboard and TKA |
| **Sakoulas, Olson et al. 2016 (178)** | MSSA, MRSA | Cefazolin and ertapenem | Checkerboard and TKA |
| **Sambatakou, Giamarellos-Bourboulis et al. 1998 (179)** | MRSA | Quinupristin-dalfopristin and ciprofloxacin, quinupristin-dalfopristin and rifampin | TKA |
| **Sanal 2018 (180)** | MRSA | Daptomycin and ceftaroline, telavancin and ceftaroline, vancomycin and ceftaroline | E-test |
| **Santimaleeworagun 2020 (181)** | MRSA | Trimethoprim-sulfamethoxazole and vancomycin | Etest and Checkerboard |
| **Saravolatz et al. 2022 (182)** | MRSA | Fosfomycin and daptomycin, fosfomycin and ceftaroline, fosfomycin and cefazolin, fosfomycin and linezolid, fosfomycin and vancomycin | Checkerboard |
| **Saverino, Debbia et al. 1992 (183)** | *S. aureus* | Flurithromycin and amoxicillin, flurithromycin and amoxicillin-clavulanic acid, flurithromycin and cefixime, flurithromycin and ciprofloxacin, flurithromycin and clindamycin, flurithromycin and netilmicin | Checkerboard and TKA |
| **Seibert, Isert et al. 1992 (184)** | MRSA | Vancomycin and cefpirome, vancomycin and cefoperazone | Checkerboard |
| **Shafiq, Bulman et al. 2017 (185)** | MRSA | Cefoxitin and ceftaroline, ceftaroline and daptomycin, ceftaroline and nafcillin, ceftaroline and vancomycin | TKA |
| **Sharma et al. 2023 (186)** | MRSA, MSSA | Ceftobiprole and vancomycin, ceftobiprole and tetracycline, ceftobiprole and doxycycline, ceftobiprole and imipenem, ceftobiprole and meropemen, ceftobiprole and mupirocin, ceftobiprole and ceftaroline fosamil, ceftobiprole and cefoxitin sodium, ceftobiprole and cefotaxime, ceftobiprole and daptomycin, ceftobiprole and gemcitabine, ceftobiprole and cloxacillin, ceftobiprole and oxacillin, ceftobiprole and dicloxacillin, ceftobiprole and nafcillin, ceftobiprole and piperacillin sodium, ceftobiprole and piperacillin-tazobactam, ceftobiprole and rifamixin, ceftobiprole and rifabutin, ceftobiprole and balofloxacin, ceftobiprole and moxifloxacin, ceftobiprole and gatifloxacin, ceftobiprole and sitafloxacin | Checkerboard |
| **Shelburne, Musher et al. 2004 (187)** | MSSA, MRSA | Vancomyin and gentamicin, vancomycin and rifampin | TKA |
| **Shi et al. 2014 (188)** | MRSA | Vancomycin and fosfomycin | TKA |
| **Silva, Araújo et al. 2011 (189)** | MRSA | Imipenem and vancomycin, rifampin and vancomycin, trimethoprim-sulfamethoxazole and vancomycin | Checkerboard and TKA |
| **Silvestri, Cirioni et al. 2012 (190)** | MSSA, MRSA | Amikacin and tigecycline, daptomycin and tigecycline, Rifampicin and tigecycline, tigecycline and imipenem, tigecycline and levofloxacin, tigecycline and teicoplanin | Checkerboard and TKA |
| **Simon and Simon 1991 (191)** | *S. aureus* | Vancomycin and cefazolin, vancomycin and flomoxef | Checkerboard |
| **Simonetti 2018 (192)** | MRSA | Fosfomycin and rifampin, fosfomycin and tigecycline | Checkerboard and TKA |
| **Singh, Bacon et al. 2009 (193)** | MRSA | Vancomycin and linezolid | TKA |
| **Smelter et al. 2022 (194)** | MSSA | Cefazolin and ertapenem | Checkerboard |
| **Smith and Eng 1985 (195)** | MRSA | Ciprofloxacin and vancomycin | TKA |
| **Smith, Eng et al. 1986 (196)** | MRSA | Gentimicin and amikacin, gentamicin and ciprofloxacin | Checkerboard and TKA |
| **Smith et al. 2016 (197)** | MRSA | Nafcillin and oritavancin, cefazolin and oritavancin, ceftaroline and oritavancin | TKA |
| **Snydman, McDermott et al. 2005 (198)** | MSSA, MRSA | Daptomycin and ampicillin, daptomycin and aztreonam, daptomycin and cefepime, daptomycin and ceftriaxone, daptomycin and gentamicin, daptomycin and imipenem, daptomycin and oxacillin | Checkerboard and TKA |
| **Soares and Trabulsi 1979 (199)** | *S. aureus* | Mezlocillin and sisomicin | Checkerboard |
| **Srisrattakarn et al. 2021 (200)** | MRSA | Vancomycin and meropenem, vancomycin and imipenem, vancomycin and cefotaxime | Checkerboard and TKA |
| **Stefani, Debbia et al. 1988 (201)** | MSSA, MRSA | Cefotetan and amikacin, cefotetan and gentamicin, cefotetan and netilmicin, cefotetan and tobramycin | Checkerboard and TKA |
| **Steigbigel, Greenman et al. 1975 (202)** | *S. aureus* | Clindamycin and penicillin, gentamicin and clindamycin, gentamicin and erythromycin, gentamicin and penicillin, penicillin and erythromycin | Checkerboard |
| **Stein, Makarewicz et al. 2016 (203)** | MSSA, MRSA | Daptomycin and rifampin | Checkerboard |
| **Stutman, Welch et al. 1984 (204)** | *S. aureus* | Nafcillin and aztreonam | Checkerboard |
| **Sueke 2010 (205)** | *S. aureus* | Ciprofloxacin and teicoplanin, meropenem and ciprofloxacin, meropenem and moxifloxacin, meropenem and linezolid, meropenem and teicoplanin, moxifloxacin and linezolid, moxifloxacin and teicoplanin | E-test |
| **Sugiura, Jono et al. 1991 (206)** | MRSA | Cefotiam and cefmetazole, cefotiam and enramycin, cefotiam and flomoxef, cefotiam and imipenem | Checkerboard and TKA |
| **Sumita and Mitsuhashi 1991 (207)** | MSSA, MRSA | Imipenem and cefazolin, imipenem and cefpiramide, meropenem and ampicillin, meropenem and ampicillin-clavulanic acid, meropenem and cefazolin, meropenem and cefmetazole, meropenem and cefotiam, meropenem and cefpiramide, meropenem and cefuzonam, meropenem and flomoxef | Checkerboard |
| **Sumon et al. 2019 (208)** | MRSA | Ceftaroline and daptomycin | TKA |
| **Sun 2011 (209)** | MRSA | Fosfomycin and minocycline | Checkerboard |
| **Sweeney and Zurenko 2003 (210)** | *S. aureus* | Amoxicillin and linezolid, amoxicillin-clavulanic acid and linezolid, ampicillin-sulbactam and linezolid, bacitracin and linezolid, cefdinir and linezolid, cefotaxime and linezolid , cefoxitin and linezolid, ceftazidime and linezolid , cephalothin and linezolid , chloramphenicol and linezolid, ciprofloxacin and linezolid, clindamycin and linezolid, difloxacin and linezolid, erythromycin and linezolid, gatifloxacin and linezolid, gentamicin and linezolid, linezolid and imipenem, linezolid and tetracycline, methicillin and linezolid, nalidixic acid and linezolid, norfloxacin and linezolid, ofloxacin and linezolid, oxacillin and linezolid, rifampin and linezolid, sparfloxacin and linezolid, teicoplanin and linezolid, trovafloxacin and linezolid, vancomycin and linezolid | Checkerboard |
| **Sy, Huang et al. 2016 (211)** | MRSA | Vancomycin and cefazolin, vancomycin and cefoxitin, vancomycin and oxacillin | Checkerboard |
| **Tang, Lai et al. 2017 (212)** | MRSA | Teicoplanin and cefazolin, teicoplanin and cefepime, teicoplanin and cefmetazole, teicoplanin and cefotaxime, vancomycin and cefotaxime, vancomycin and cefazolin, vancomycin and cefepime, vancomycin and cefmetazole | Checkerboard and TKA |
| **Thamlikitkul 1991 (213)** | *S. aureus* | Gentamicin and teicoplanin, gentamicin and vancomycin | TKA |
| **Totsuka, Shiseki et al. 1999 (214)** | MRSA | Vancomycin and imipenem | Checkerboard and TKA |
| **Toyokawa, Asari et al. 2003 (215)** | MRSA | Teicoplanin and cefozopran, vancomycin and cefazopran | Checkerboard and TKA |
| **Traub et al. 1986 (216)** | *S. aureus* | Imipenem and vancomycin | Checkerboard and TKA |
| **Tran and Rybak (217)** | MRSA | Vancomycin and cefazolin, vancomycin and cefepime, vancomycin and ceftaroline, vancomycin and nafcillin | TKA |
| **Tsai et al. 2022 (218)** | MRSA | Ceftaroline and linezolid, ceftaroline and daptomycin, ceftaroline and vancomycin | Checkerboard and TKA |
| **Tsuji and rybak 2006 (219)** | MSSA, MRSA | Daptomycin and ampicillin-sulbactam, daptomycin and linezolid, daptomycin and quinupristin-dalfopristin, daptomycin and vancomycin, gentamicin and ampicillin-sulbactam, gentamicin and daptomycin, gentamixin and quinupristin-dalfopristin, gentamicin and vancomycin, linezolid and ampicillin-sulbactam, quinupristin-dalfopristin and ampicillin-sulbactam, quinupristin-dalfopristin and linezolid, rifampin and ampicillin-sulbactam, rifampin and daptomycin, rifampin and gentamicin, rifampin and linezolid, rifampin and quinupristin-dalfopristin, rifampin and vancomycin, vancomycin and ampicillin-sulbactam, vancomycin and linezoli, vancomyin and quinupristin-dalfopristin | TKA and E-test |
| **Tuazon and Miller 1984 (220)** | MSSA, MRSA | Rifampin and vancomycin, rifampin and teichomycin A2 | Checkerboard |
| **Uete and Matsuo 1995 (221)** | MSSA, MRSA | Imipenem and cefazolin, imipenem and cefoperazone, imipenem and cefotiam, imipenem and cephalothin, imipenem and cephamandole | Checkerboard |
| **Uete and Matsuo 1995 (222)** | MSSA, MRSA | Cefamandole and cefmetazole, cefmetazole and cefotiam, cefoperazone and cefmetazole | Checkerboard |
| **Ulloa 2020 (223)** | MSSA | Cefazolin and ertapenem, nafcillin and ertapenem | Checkerboard and TKA |
| **Utsui, 1986 (224)** | MRSA | Fosfomycin and cefmetazole, fosfomycin and cefotaxime, fosfomycin and cephaloridine | Checkerboard and TKA |
| **Valderrama, Alfaro et al. 2020 (225)** | MRSA | Linezolid and amikacin, linezolid and doripenem, linezolid and fosfomycin, linezolid and gentamicin, linezolid and imipenem, linezolid and meropenem, linezolid and plazomicin | Checkerboard and TKA |
| **Van der Auwera and Joly 1987 (226)** | MSSA, MRSA | Ciprofloxacin and LM427 (rifabutin), coumermycin and ciprofloxacin, coumermycin and LM427 (rifabutin), teicoplanin and LM427 (rifabutin) | TKA |
| **Van der Auwera and Klastersky 1986 (227)** | MRSA | Coumermycin and ciprofloxacin, coumermycin and LM427 (rifabutin), coumermycin and rifampin | TKA |
| **Van der Auwera, Vandermies et al. 1987 (228)** | MSSA, MRSA | Coumermycin and merafloxacin | TKA |
| **Verbist and Verhaegen 1984 (229)** | MSSA, MRSA | Temocillin and cephazolin, temocillin and flucloxacillin | Checkerboard |
| **Vidaillac, Leonard et al. 2010 (230)** | MRSA | Tobramycin and ceftaroline, tobramycin and vancomycin | Checkerboard and TKA |
| **Vouillamoz et al. 2000 (231)** | MRSA | Quinupristin-dalfopristin and cefepime | Checkerboard and TKA |
| **Walsh, Auger et al. 1986 (232)** | MRSA | Rifampin and novobiocin, rifampin and vancomycin, vancomycin and novobiocin | Checkerboard and TKA |
| **Watanakunakorm and Glotzbecker 1974 (233)** | MSSA | Nafcillin and tobramycin, nafcillin and gentamicin | Checkerboard and TKA |
| **Watanakunakorn and Glotzbecker 1977 (234)** | MSSA | Nafcillin and netilmicin, nafcillin and sisomicin, oxacillin and netilmicin, oxacillin and sisomicin | TKA |
| **Watanakunakorn and Glotzbecker 1979 (235)** | MSSA | Carbenicillin and amikacin, carbenicillin and gentamicin, carbenicillin and tobramycin, ticarcillin and amikacin, ticarcillin and gentimicin, ticarcillin and tobramycin | TKA |
| **Watanakunakorn and Glotzbecker 1980 (236)** | *S.aureus* | Amikacin and clindamycin, gentamicin and clindamycin, tobramycin and clindamycin | TKA |
| **Watanakunakorn and Guerriero 1981 (237)** | *S. aureus* | Rifampin and vancomycin | TKA |
| **Watanakunakorn and Tisone 1982 (238)** | MSSA, MRSA | Vancomyin and gentamicin, vancomycin and tobramycin | TKA |
| **Watanakunakorn and Tisone 1982 (239)** | MSSA | Rifampin and nafcillin, rifampin and oxacillin | TKA |
| **Wattanapaisal et al. 2022 (240)** | MRSA | Fusidic acid and ciprofloxacin, fusidic acid and clindamycin, fusidic acid and rifampin, fusidic acid and trimethoprim-sulfamethoxazole, fusidic acid and doxycycline | Checkerboard |
| **Weber, Boussougant et al. 1987 (241)** | MSSA, MRSA | Fosfomycin and ofloxacin, vancomycin and ofloxacin | Checkerboard and TKA |
| **Welch, Bawdon et al. 1984 (242)** | MSSA, MRSA | Clindamycin and cefpimizole | Checkerboard |
| **Werth et al. 2013 (243)** | MRSA | Vancomycin and oxacillin, vancomycin and ceftriaxone | TKA |
| **Werth et al. 2017 (244)** | MRSA | Tedizolid and doxycycline, tedizolid and trimethoprim-sulfamethoxazole, Tedizolid and moxifloxacin, tedizolid and rifampin | TKA |
| **White 1996 (245)** | MSSA | Cefepime and ciprofloxacin, Cefepime and tobramycin, Ceftazidime and ciprofloxacin, Ceftazidime and tobramycin | Checkerboard, E-test and TKA |
| **Wicha, Kees et al. 2015 (246)** | MSSA | Meropenem and linezolid, Meropenem and vancomycin | Checkerboard and TKA |
| **Wise, Ashby et al. 1989 (247)** | MSSA, MRSA | Meropenem and vancomycin | TKA |
| **Wise et al. 1979 (248)** | *S. aureus* | Cefoxitin and mezlocillin | Checkerboard |
| **Xhemali, Smith et al. 2019 (249)** | MRSA | Dalbavancin and cefazolin, dalbavancin and cefepime, dalbavancin and ceftaroline, dalbavancin and ertapenem, dalbavancin and oxacillin | TKA |
| **Xie, Jiang et al. 2021 (250)** | MSSA | Fosfomycin and Linezolid | TKA |
| **Xu, Xu et al. 2018 (251)** | MRSA | Roxithromycin and doxycycline, vancomycin and fosfomycin, vancomycin and ofloxacin | Checkerboard |
| **Xu-hong, Falagas et al. 2014 (252)** | MRSA | Fosfomycin and linezolid | Checkerboard |
| **Yang et al. 2010 (253)** | MRSA | Daptomycin and oxacillin | TKA |
| **Yang et al. 2017 (254)** | *S. aureus* | Amoxicillin and ceftiofur, amoxicillin and kanamycin, amoxicillin and colistin sulfate, ceftiofur and kanamycin, ceftiofur and colistin sulfate, ceftiofur and sulfadimidine, ceftiofur and enrofloxacin, ceftiofur and rifampin, kanamycin and colistin sulfate, kanamycin and sulfadimidine, colistin sulfate and doxycycline, colistin sulfate and florfenicol, colistin sulfate and sulfadimidine, doxycycline and rifampin, florfenicol and sulfadimidine | Checkerboard |
| **Yousef, Tawil et al. 1985 (255)** | *S. aureus* | Amoxicillin and dicloxacillin | Checkerboard |
| **You et al. 2000 (256)** | MRSA | Arbekacin and vancomycin, gentamicin and vancomycin | TKA |
| **Yu et al. 2010 (257)** | MRSA | Fusidic acid and fosfomycin | Checkerboard |
| **Yu, Huang et al. 2020 (258)** | MRSA | Clindamycin and oxacillin, levofloxacin and oxacillin, gentamicin and oxacillin, rifampin and vancomycin, vancomycin and oxacillin, clindamycin and fosfomycin | TKA |
| **Zakaria et al. 2012 (259)** | MRSA | Ceftriaxone and moxifloxacin, ceftriaxone and levofloxacin | Checkerboard |
| **Zarrouk, Bozdogan et al. 2001 (260)** | MSSA, MRSA | Quinupristin-dalfopristin and rifampin | TKA |
| **Zhang et al. 2021 (261)** | MRSA | Vancomycin and ceftriaxone, vancomycin and cephalexin, vancomycin and cefoxitin, vancomycin and ceftaroline, vancomycin and nafcillin, vancomycin and meropenem, dalbavancin and ceftriaxone, dalbavancin and cephalexin, dalbavancin and cefoxitin, dalbavancin and ceftaroline, dalbavancin and nafcillin, dalbavancin and meropenem, daptomycin and nafcillin, daptomycin and ceftriaxone, daptomycin and cephalexin, daptomycin and cefoxitin, daptomycin and ceftaroline | TKA |
| **Zhou et al. 2018 (262)** | MRSA | Azithromycin and daptomycin | Checkerboard |
| **Zhou et al. 2019 (263)** | MRSA | Linezolid and rifampin | Checkerboard and TKA |
| **Zhou et al. 2023 (264)** | MSSA, MRSA | Linezolid and rifampin | Checkerboard and TKA |
| **Zinner, Lagast et al. 1981 (265)** | *S. aureus* | Rifampin and vancomycin, rifampin and methicillin | Checkerboard and TKA |

**Supplementary Table 2**. Summary of characteristics of included studies

* Studies evaluating S. aureus included those that evaluated both MRSA and MSSA and pooled the results together.

|  | **TKA** | | **Checkerboard** | | **Combined Gradient Diffusion method** | | **More than one method**** | |
| --- | --- | --- | --- | --- | --- | --- | --- | --- |
| **Isolate (n)** | Standard criteria met  (n, %) | No criteria provided  (n, %) | Standard criteria met  (n, %) | No criteria provided  (n, %) | Standard criteria met (n, %) | No criteria provided  (n, %) | Standard criteria for at least one method (n, %) | No criteria provided for either method  (n, %) |
| **All (314)** | 32 / 125  (25.6%) | 25 / 125  (20.0%) | 74 / 96 (77.1%) | 9 / 96  (9.4%) | 8 / 9 (88.9%) | 1 / 9  (11.1%) | 60 / 84 (71.4%) | 10 / 84 (11.9%) |
| **MRSA (164)** | 25 / 82 (30.5%) | 14 / 82 (17.1)% | 31 / 38 (81.6%) | 3 / 38 (7.9%) | 6 / 7 (85.7%) | 1 / 7 (14.3%) | 29 / 36 (80.6%) | 4 / 36 (8.3%) |
| **MSSA (34)** | 2 / 13 (15.4%) | 5 / 13 (38.5%) | 8 / 9 (88.9%) | 1 / 9 (11.1%) | - | - | 8 / 12 (66.7%) | 1 / 12 (8.3%) |
| **MRSA and MSSA (65)** | 5 / 22 (22.7%) | 3 / 22 (13.6%) | 14 / 18 (77.8%) | 3 / 18 (16.7%) | 1 / 1 (100%) | 0 / 1 (0%) | 16 / 24 (66.7%) | 4 / 24 (16.7%) |
| ***S. aureus** (51)** | 0 / 8 (0%) | 3 / 8 (20%) | 21 / 31 (67.7%) | 2 / 31 (6.5%) | 1 / 1 (100%) | 0 / 1 (0%) | 7 / 11 (63.6%) | 1 / 11 (9.1%) |

**Supplementary Table 3**. Studies meeting standard criteria for synergy testing according to method.

*Studies evaluating *S. aureus* performed synergy testing for both MSSA isolates and MRSA isolates and presented the findings together or did not provide information on the methicillin-resistance profile of the isolates.

**The more than one method category consists of studies using more than one eligible method of synergy assessment in their analysis

**Supplementary References**

1. Abdul-Mutakabbir JC, Kebriaei R, Stamper KC, Sheikh Z, Maassen PT, Lev KL, Rybak MJ. 2020. Dalbavancin, Vancomycin and Daptomycin Alone and in Combination with Cefazolin against Resistant Phenotypes of Staphylococcus aureus in a Pharmacokinetic/Pharmacodynamic Model. Antibiotics (Basel) 9.

2. Aeschlimann JR, Allen GP, Hershberger E, Rybak MJ. 2000. Activities of LY333328 and vancomycin administered alone or in combination with gentamicin against three strains of vancomycin-intermediate Staphylococcus aureus in an in vitro pharmacodynamic infection model. Antimicrob Agents Chemother 44:2991-8.

3. Ahmad NM, Rojtman AD. 2010. Successful treatment of daptomycin-nonsusceptible methicillin-resistant Staphylococcus aureus bacteremia with the addition of rifampin to daptomycin. Ann Pharmacother 44:918-21.

4. Ahmed Z, Khan SS, Khan M. 2013. In vitro trials of some antimicrobial combinations against Staphylococcus aureus and Pseudomonas aeruginosa. Saudi Journal of Biological Sciences 20:79-83.

5. Aktas G. 2017. In-vitro activity of ceftriaxone combined with newer agents against MRSA. J Chemother 29:383-385.

6. Aktas G. 2021. Efficacy of vancomycin in combination with various antimicrobial agents against clinical methicillin resistant Staphylococcus aureus strains. Pak J Med Sci 37:151-156.

7. Aktas G, Derbentli S. 2017. In vitro activity of daptomycin combinations with rifampicin, gentamicin, fosfomycin and fusidic acid against MRSA strains. J Glob Antimicrob Resist 10:223-227.

8. Aktas G, Derbentli S. 2017. In vitro activity of daptomycin combined with dalbavancin and linezolid, and dalbavancin with linezolid against MRSA strains. J Antimicrob Chemother 72:441-443.

9. Alou L, Cafini F, Sevillano D, Unzueta I, Prieto J. 2004. In vitro activity of mupirocin and amoxicillin-clavulanate alone and in combination against staphylococci including those resistant to methicillin. Int J Antimicrob Agents 23:513-6.

10. Arpi M, Jørgensen PE, Pedersen HF. 1986. In vitro studies of the synergism of piperacillin and netilmicin against blood culture isolates. Chemotherapy 32:68-74.

11. Azap OK, Arslan H, Timurkaynak F, Yapar G, Cagir U. 2007. IN VITRO SYNERGY BETWEEN GLYCOPEPTIDES AND CARBAPENEMS AGAINST METHICILLIN-RESISTANT STAPHYLOCOCCUS AUREUS. Gazi Medical Journal 18:159-162.

12. Bai J, Zhu X, Zhao K, Yan Y, Xu T, Wang J, Zheng J, Huang W, Shi L, Shang Y, Lv Z, Wang X, Wu Y, Qu D. 2019. The role of ArlRS in regulating oxacillin susceptibility in methicillin-resistant Staphylococcus aureus indicates it is a potential target for antimicrobial resistance breakers. Emerg Microbes Infect 8:503-515.

13. Bakhtiar M, Selwyn S. 1989. Beta-lactamase stability and antibacterial activity of cefpirome alone and in combination with other antibiotics. Drugs Exp Clin Res 15:477-82.

14. Bakthavatchalam YD, Ralph R, Veeraraghavan B, Babu P, Munusamy E. 2019. Evidence from an In Vitro Study: Is Oxacillin Plus Vancomycin a Better Choice for Heteroresistant Vancomycin-Intermediate Staphylococcus aureus? Infect Dis Ther 8:51-62.

15. Baldoni D, Tafin UF, Aeppli S, Angevaare E, Oliva A, Haschke M, Zimmerli W, Trampuz A. 2013. Activity of dalbavancin, alone and in combination with rifampicin, against meticillin-resistant Staphylococcus aureus in a foreign-body infection model. International Journal of Antimicrobial Agents 42:220-225.

16. Baltch AL, Bassey C, Fanciullo G, Smith RP. 1987. In-vitro antimicrobial activity of enoxacin in combination with eight other antibiotics against Pseudomonas aeruginosa, Enterobacteriaceae and Staphylococcus aureus. J Antimicrob Chemother 19:45-8.

17. Baltch AL, Ritz WJ, Bopp LH, Michelsen PB, Smith RP. 2007. Antimicrobial activities of daptomycin, vancomycin, and oxacillin in human monocytes and of daptomycin in combination with gentamicin and/or rifampin in human monocytes and in broth against Staphylococcus aureus. Antimicrob Agents Chemother 51:1559-62.

18. Baltch AL, Ritz WJ, Bopp LH, Michelsen P, Smith RP. 2008. Activities of daptomycin and comparative antimicrobials, singly and in combination, against extracellular and intracellular Staphylococcus aureus and its stable small-colony variant in human monocyte-derived macrophages and in broth. Antimicrob Agents Chemother 52:1829-33.

19. Banerjee R, Fernandez MG, Enthaler N, Graml C, Greenwood-Quaintance KE, Patel R. 2013. Combinations of cefoxitin plus other β-lactams are synergistic in vitro against community associated methicillin-resistant Staphylococcus aureus. Eur J Clin Microbiol Infect Dis 32:827-33.

20. Barber KE, Werth BJ, Ireland CE, Stone NE, Nonejuie P, Sakoulas G, Pogliano J, Rybak MJ. 2014. Potent synergy of ceftobiprole plus daptomycin against multiple strains of Staphylococcus aureus with various resistance phenotypes. J Antimicrob Chemother 69:3006-10.

21. Barr JG, Smyth ET, Hogg GM. 1990. In vitro antimicrobial activity of imipenem in combination with vancomycin or teicoplanin against Staphylococcus aureus and Staphylococcus epidermidis. Eur J Clin Microbiol Infect Dis 9:804-9.

22. Batard E, Jacqueline C, Boutoille D, Hamel A, Drugeon HB, Asseray N, Leclercq R, Caillon J, Potel G, Bugnon D. 2002. Combination of quinupristin-dalfopristin and gentamicin against methicillin-resistant Staphylococcus aureus: experimental rabbit endocarditis study. Antimicrob Agents Chemother 46:2174-8.

23. Bayer AS, Lam K. 1985. Efficacy of vancomycin plus rifampin in experimental aortic-valve endocarditis due to methicillin-resistant Staphylococcus aureus: in vitro-in vivo correlations. J Infect Dis 151:157-65.

24. Bayer AS, Morrison JO. 1984. Disparity between timed-kill and checkerboard methods for determination of in vitro bactericidal interactions of vancomycin plus rifampin versus methicillin-susceptible and -resistant Staphylococcus aureus. Antimicrob Agents Chemother 26:220-3.

25. Belley A, Neesham-Grenon E, Arhin FF, McKay GA, Parr TR, Jr., Moeck G. 2008. Assessment by time-kill methodology of the synergistic effects of oritavancin in combination with other antimicrobial agents against Staphylococcus aureus. Antimicrob Agents Chemother 52:3820-2.

26. Bergeret M, Raymond J. 1999. In-vitro bactericidal activity of cefpirome and cefamandole in combination with glycopeptides against methicillin-resistant Staphylococcus aureus. J Antimicrob Chemother 43:291-4.

27. Bergeret M, Boutros N, Raymond J. 2004. In vitro combined bactericidal activity of cefpirome and glycopeptides against glycopeptides and oxacillin-resistant staphylococci. Int J Antimicrob Agents 23:247-53.

28. Betts JW, Abdul Momin HF, Phee LM, Wareham DW. 2018. Comparative activity of tedizolid and glycopeptide combination therapies for the treatment of Staphylococcus aureus infections: an in vitro and in vivo evaluation against strains with reduced susceptibility to glycopeptides. J Med Microbiol 67:265-271.

29. Biedenbach DJ, Rhomberg PR, Mendes RE, Jones RN. 2010. Spectrum of activity, mutation rates, synergistic interactions, and the effects of pH and serum proteins for fusidic acid (CEM-102). Diagn Microbiol Infect Dis 66:301-307.

30. Bishr AS, Abdelaziz SM, Yahia IS, Yassien MA, Hassouna NA, Aboshanab KM. 2021. Association of Macrolide Resistance Genotypes and Synergistic Antibiotic Combinations for Combating Macrolide-Resistant MRSA Recovered from Hospitalized Patients. Biology (Basel) 10.

31. Borowski J, Linda H. 1977. Combined action of fosfomycin with beta-lactam and aminoglycoside antibiotics. Chemotherapy 23:82-5.

32. Boudjemaa R, Bri, et R, Fontaine-Aupart MP, Steenkeste K. 2017. How do fluorescence spectroscopy and multimodal fluorescence imaging help to dissect the enhanced efficiency of the vancomycin-rifampin combination against Staphylococcus aureus infections? Photochem Photobiol Sci 16:1391-1399.

33. Boudrioua A, Li Y, Hartke A, Giraud C. 2020. Opposite effect of vancomycin and D-Cycloserine combination in both vancomycin resistant Staphylococcus aureus and enterococci. FEMS Microbiol Lett 367.

34. Brandt CM, Rouse MS, Tallan BM, Wilson WR, Steckelberg JM. 1994. Failure of time-kill synergy studies using subinhibitory antimicrobial concentrations to predict in vivo antagonism of cephalosporin-rifampin combinations against Staphylococcus aureus. Antimicrob Agents Chemother 38:2191-3.

35. Broussou DC, Toutain PL, Woehrle F, El Garch F, Bousquet-Melou A, Ferran AA. 2019. Comparison of in vitro static and dynamic assays to evaluate the efficacy of an antimicrobial drug combination against Staphylococcus aureus. Plos One 14.

36. Bulger RJ. 1967. In-vitro activity of cephalothin/kanamycin and methicillin/kanamycin combinations against methicillin-resistant Staphylococcus aureus. Lancet 1:17-9.

37. Cabellos C, Garrigós C, Taberner F, Force E, Pachón-Ibañez ME. 2014. Experimental study of the efficacy of linezolid alone and in combinations against experimental meningitis due to Staphylococcus aureus strains with decreased susceptibility to beta-lactams and glycopeptides. J Infect Chemother 20:563-8.

38. Campanile F, Bongiorno D, Mongelli G, Zanghi G, Stefani S. 2019. Bactericidal activity of ceftobiprole combined with different antibiotics against selected Gram-positive isolates. Diagn Microbiol Infect Dis 93:77-81.

39. Carricajo A, Vermesch R, Aubert G. 2001. In vitro activity of cefpirome and vancomycin in combination against gentamicin-susceptible and gentamicin-resistant Staphylococcus aureus. Clin Microbiol Infect 7:218-26.

40. Castaneda X, Garcia-De-la-Maria C, Gasch O, Pericas JM, Soy D, Canas-Pacheco MA, Falces C, Garcia-Gonzalez J, Hernandez-Meneses M, Vidal B, Almela M, Quintana E, Tolosana JM, Fuster D, Llopis J, Dahl A, Moreno A, Marco F, Miro JM, Hosp Clinic Endocarditis Study G. 2021. Effectiveness of vancomycin plus cloxacillin compared with vancomycin, cloxacillin and daptomycin single therapies in the treatment of methicillin-resistant and methicillin-susceptible Staphylococcus aureus in a rabbit model of experimental endocarditis. Journal of Antimicrobial Chemotherapy 76:1539-1546.

41. Chai D, Liu X, Wang R, Bai Y, Cai Y. 2016. Efficacy of Linezolid and Fosfomycin in Catheter-Related Biofilm Infection Caused by Methicillin-Resistant Staphylococcus aureus. Biomed Res Int 2016:6413982.

42. Chang SC, Hsieh WC, Luh KT, Ho SW. 1989. Effects of antibiotic combinations on methicillin-resistant Staphylococcus aureus in vitro. Taiwan Yi Xue Hui Za Zhi 88:488-92.

43. Chen H, Li L, Liu Y, Wu M, Xu S, Zhang G, Qi C, Du Y, Wang M, Li J, Huang X. 2018. In vitro activity and post-antibiotic effects of linezolid in combination with fosfomycin against clinical isolates of Staphylococcus aureus. Infect Drug Resist 11:2107-2115.

44. Chen RM, Takahashi K, Kanno H, Kuriyama T. 1988. Synergistic activities of combinations of antibiotics against methicillin-resistant Staphylococcus aureus. Gaoxiong Yi Xue Ke Xue Za Zhi 4:323-8.

45. Chin NX, Neu HC. 1990. Combination of ofloxacin and other antimicrobial agents. J Chemother 2:343-7.

46. Chin NX, Jules K, Neu HC. 1986. Synergy of ciprofloxacin and azlocillin in vitro and in a neutropenic mouse model of infection. Eur J Clin Microbiol 5:23-8.

47. Choi S, Moon SM, Park SJ, Lee SC, Jung KH, Sung HS, Kim MN, Jung J, Kim MJ, Kim SH, Lee SO, Choi SH, Jeong JY, Woo JH, Kim YS, Chong YP. 2020. Antagonistic Effect of Colistin on Vancomycin Activity against Methicillin-Resistant Staphylococcus aureus in In Vitro and In Vivo Studies. Antimicrob Agents Chemother 64.

48. Cilli F, Aydemir S, Tunger A. 2006. In vitro activity of daptomycin alone and in combination with various antimicrobials against Gram-positive cocci. J Chemother 18:27-32.

49. Claeys KC, Smith JR, Casapao AM, Mynatt RP, Avery L, Shroff A, Yamamura D, Davis SL, Rybak MJ. 2015. Impact of the combination of daptomycin and trimethoprim-sulfamethoxazole on clinical outcomes in methicillin-resistant Staphylococcus aureus infections. Antimicrob Agents Chemother 59:1969-76.

50. Climo MW, Patron RL, Archer GL. 1999. Combinations of vancomycin and beta-lactams are synergistic against staphylococci with reduced susceptibilities to vancomycin. Antimicrob Agents Chemother 43:1747-53.

51. Coban AY, Deveci A, Acicbe O, Fisgin NT, Ciftci A, Dokuzoguz B, Durupinar B. 2010. Investigation of in vitro effects of daptomycin, tigecycline and teicoplanin combinations against MRSA, VISA and VRE strains. African Journal of Microbiology Research 4:2269-2274.

52. Credito K, Lin G, Appelbaum PC. 2007. Activity of daptomycin alone and in combination with rifampin and gentamicin against Staphylococcus aureus assessed by time-kill methodology. Antimicrob Agents Chemother 51:1504-7.

53. D'Arezzo S, Mazzarelli A, Venditti C, Nisii C, Petrosillo N, De Giuli C, Vulcano A, Paglia MG, Bordi E, Di Caro A, Taglietti F. 2017. Ceftaroline Plus Ampicillin Against Gram-Positive Organisms: Results from E-Test Synergy Assays. Microb Drug Resist 23:507-515.

54. Darouiche RO, Raad I, Bodey GP, Musher DM. 1995. Antibiotic susceptibility of staphylococcal isolates from patients with vascular catheter-related bacteremia: potential role of the combination of minocycline and rifampin. Int J Antimicrob Agents 6:31-6.

55. Daschner FD. 1976. Combination of bacteriostatic and bactericidal drugs: lack of significant in vitro antagonism between penicillin, cephalothin, and rolitetracycline. Antimicrob Agents Chemother 10:802-8.

56. Dawis MA, Isenberg HD, France KA, Jenkins SG. 2003. In vitro activity of gatifloxacin alone and in combination with cefepime, meropenem, piperacillin and gentamicin against multidrug-resistant organisms. J Antimicrob Chemother 51:1203-11.

57. del Río A, García-de-la-Mària C, Entenza JM, Gasch O, Armero Y, Soy D, Mestres CA, Pericás JM, Falces C, Ninot S, Almela M, Cervera C, Gatell JM, Moreno A, Moreillon P, Marco F, Miró JM. 2016. Fosfomycin plus β-Lactams as Synergistic Bactericidal Combinations for Experimental Endocarditis Due to Methicillin-Resistant and Glycopeptide-Intermediate Staphylococcus aureus. Antimicrob Agents Chemother 60:478-86.

58. Dhand A, Bayer AS, Pogliano J, Yang SJ, Bolaris M, Nizet V, Wang GQ, Sakoulas G. 2011. Use of Antistaphylococcal beta-Lactams to Increase Daptomycin Activity in Eradicating Persistent Bacteremia Due to Methicillin-Resistant Staphylococcus aureus: Role of Enhanced Daptomycin Binding. Clinical Infectious Diseases 53:158-163.

59. Dilworth TJ, Sanchez D, Anderson B, DeAngelis H, Mercier RC. 2019. Exploring the Role of Piperacillin and Tazobactam in Combination with Vancomycin against Methicillin-Resistant Staphylococcus aureus. Chemotherapy 64:233-237.

60. Dilworth TJ, Sliwinski J, Ryan K, Dodd M, Mercier RC. 2014. Evaluation of vancomycin in combination with piperacillin-tazobactam or oxacillin against clinical methicillin-resistant Staphylococcus aureus Isolates and vancomycin-intermediate S. aureus isolates in vitro. Antimicrob Agents Chemother 58:1028-33.

61. Dixson S, Brumfitt W, Hamilton-Miller JM. 1985. In vitro activity of combinations of antibiotics against Staphylococcus aureus resistant to gentamicin and methicillin. Infection 13:35-8.

62. Domaracki BE, Evans AM, Venezia RA. 2000. Vancomycin and oxacillin synergy for methicillin-resistant staphylococci. Antimicrob Agents Chemother 44:1394-6.

63. Domenech A, Ribes S, Cabellos C, Taberner F, Tubau F, Dominguez MA, Montero A, Linares J, Ariza J, Gudiol F. 2005. Experimental study on the efficacy of combinations of glycopeptides and beta-lactams against Staphylococcus aureus with reduced susceptibility to glycopeptides. Journal of Antimicrobial Chemotherapy 56:709-716.

64. Drago L, De Vecchi E, Nicola L, Gismondo MR. 2007. In vitro evaluation of antibiotics' combinations for empirical therapy of suspected methicillin resistant Staphylococcus aureus severe respiratory infections. BMC Infect Dis 7:111.

65. Drusano GL, de Jongh C, Newman K, Joshi J, Wharton R, Moody MR, Schimpff SC. 1985. Moxalactam and piperacillin: a study of in vitro characteristics and pharmacokinetics in cancer patients. Infection 13:20-6.

66. Duez JM, Adochitei A, Péchinot A, Siebor E, Sixt N, Neuwirth C. 2008. In vitro combinations of five intravenous antibiotics with dalfopristin-quinupristin against Staphylococcus aureus in a 3-dimensional model. J Chemother 20:684-9.

67. Dumitrescu O, Badiou C, Bes M, Reverdy ME, enesch F, Etienne J, Lina G. 2008. Effect of antibiotics, alone and in combination, on Panton-Valentine leukocidin production by a Staphylococcus aureus reference strain. Clin Microbiol Infect 14:384-8.

68. Dupieux C, Trouillet-Assant S, Camus C, Abad L, Bes M, Benito Y, Chidiac C, Lustig S, Ferry T, Valour F, Laurent F. 2017. Intraosteoblastic activity of daptomycin in combination with oxacillin and ceftaroline against MSSA and MRSA. J Antimicrob Chemother 72:3353-3356.

69. Entenza JM, Veloso TR, Vouillamoz J, Giddey M, Majcherczyk P, Moreillon P. 2011. In vivo synergism of ceftobiprole and vancomycin against experimental endocarditis due to vancomycin-intermediate Staphylococcus aureus. Antimicrob Agents Chemother 55:3977-84.

70. Ermertcan S, Hosgor-Limoncu M, Tasli H, Cilli F, Cosar G. 2010. In Vitro Activity of Linezolid in Combination with Vancomycin, Teicoplanin, Fusidic Acid, and Ciprofloxacin Against Gram-Positive Pathogens. Turkiye Klinikleri Tip Bilimleri Dergisi 30:59-64.

71. Fantin B, Leclercq R, Duval J, Carbon C. 1993. FUSIDIC ACID ALONE OR IN COMBINATION WITH VANCOMYCIN FOR THERAPY OF EXPERIMENTAL ENDOCARDITIS DUE TO METHICILLIN-RESISTANT STAPHYLOCOCCUS-AUREUS. Antimicrob Agents Chemother 37:2466-2469.

72. Fern, ez J, Abbanat D, Shang W, He W, Amsler K, Hastings J, Queenan AM, Melton JL, Barron AM, Flamm RK, Lynch AS. 2012. Synergistic activity of ceftobiprole and vancomycin in a rat model of infective endocarditis caused by methicillin-resistant and glycopeptide-intermediate Staphylococcus aureus. Antimicrob Agents Chemother 56:1476-84.

73. Ferrara A, Dos Santos C, Cimbro M, Gialdroni Grassi G. 1997. Effect of different combinations of sparfloxacin, oxacillin, and fosfomycin against methicillin-resistant staphylococci. Eur J Clin Microbiol Infect Dis 16:535-7.

74. Ferrara A, Grassi G, Grassi FA, Piccioni PD, Gialdroni Grassi G. 1989. Bactericidal activity of meropenem and interactions with other antibiotics. J Antimicrob Chemother 24:239-50.

75. Flamm RK, Farrell DJ, Sader HS, Jones RN. 2014. Antimicrobial activity of ceftaroline combined with avibactam tested against bacterial organisms isolated from acute bacterial skin and skin structure infections in United States medical centers (2010-2012). Diagn Microbiol Infect Dis 78:449-56.

76. Fuchs PC, Barry AL, Brown SD. 2001. Interactions of quinupristin-dalfopristin with eight other antibiotics as measured by time-kill studies with 10 strains of Staphylococcus aureus for which quinupristin-dalfopristin alone was not bactericidal. Antimicrob Agents Chemother 45:2662-5.

77. García AB, Candel FJ, López L, Chiarella F, Viñuela-Prieto JM. 2016. In vitro ceftaroline combinations against meticillin-resistant Staphylococcus aureus. J Med Microbiol 65:1119-1122.

78. García-de-la-Mària C, Gasch O, García-Gonzalez J, Soy D, Shaw E, Ambrosioni J, Almela M, Pericàs JM, Tellez A, Falces C, Hern, ez-Meneses M, oval E, Quintana E, Vidal B, Tolosana JM, Fuster D, Llopis J, Pujol M, Moreno A, Marco F, Miró JM. 2018. The Combination of Daptomycin and Fosfomycin Has Synergistic, Potent, and Rapid Bactericidal Activity against Methicillin-Resistant Staphylococcus aureus in a Rabbit Model of Experimental Endocarditis. Antimicrob Agents Chemother 62.

79. García-de-la-Mària C, Gasch O, Castañeda X, García-González J, Soy D, Cañas MA, Ambrosioni J, Almela M, Pericàs JM, Téllez A, Falces C, Hernández-Meneses M, oval E, Quintana E, Vidal B, Tolosana JM, Fuster D, Llopis J, Moreno A, Marco F, Miró JM. 2020. Cloxacillin or fosfomycin plus daptomycin combinations are more active than cloxacillin monotherapy or combined with gentamicin against MSSA in a rabbit model of experimental endocarditis. J Antimicrob Chemother 75:3586-3592.

80. Garrigós C, Murillo O, Lora-Tamayo J, Verdaguer R, Tubau F, Cabellos C, Cabo J, Ariza J. 2012. Efficacy of daptomycin-cloxacillin combination in experimental foreign-body infection due to methicillin-resistant Staphylococcus aureus. Antimicrob Agents Chemother 56:3806-11.

81. Gatermann S, Schulz E, Marre R. 1989. The microbiological efficacy of the combination of fosfomycin and vancomycin against clinically relevant staphylococci. Infection 17:35-7.

82. Gil Romero Y, Gómez-Garcés JL. 2020. In vitro activity of ceftaroline in combination with other antimicrobials active against Staphylococcus spp. Enferm Infecc Microbiol Clin (Engl Ed) 38:25-27.

83. Gonzales PR, Pesesky MW, Bouley R, Ballard A, Biddy BA, Suckow MA, Wolter WR, Schroeder VA, Burnham CAD, Mobashery S, Chang M, Dantas G. 2015. Synergistic, collaterally sensitive beta-lactam combinations suppress resistance in MRSA. Nature Chemical Biology 11:855-U71.

84. Goto Y, Hiramatsu K, Nasu M. 1999. Improved efficacy with nonsimultaneous administration of netilmicin and minocycline against methicillin-resistant Staphylococcus aureus in in vitro and in vivo models. International Journal of Antimicrobial Agents 11:39-46.

85. Gould IM, Milne K. 1997. In-vitro pharmacodynamic studies of piperacillin/tazobactam with gentamicin and ciprofloxacin. J Antimicrob Chemother 39:53-61.

86. Gradelski E, Kolek B, Bonner DP, Valera L, Minassian B, Fung-Tomc J. 2001. Activity of gatifloxacin and ciprofloxacin in combination with other antimicrobial agents. Int J Antimicrob Agents 17:103-7.

87. Grassi GG, Alesina R, Ferrara A, Peona V. 1983. In-vitro antibacterial activity of cefotetan. J Antimicrob Chemother 11:45-58.

88. Grif K, Dierich MP, Pfaller K, Miglioli PA, Allerberger F. 2001. In vitro activity of fosfomycin in combination with various antistaphylococcal substances. J Antimicrob Chemother 48:209-17.

89. Hackemann VCJ, Hagel S, Jandt KD, Rödel J, Löffler B, Tuchscherr L. 2023. The Controversial Effect of Antibiotics on Methicillin-Sensitive S. aureus: A Comparative In Vitro Study. Int J Mol Sci 24.

90. Henson KER, Yim J, Smith JR, Sakoulas G, Rybak MJ. 2017. beta-Lactamase Inhibitors Enhance the Synergy between beta-Lactam Antibiotics and Daptomycin against Methicillin-Resistant Staphylococcus aureus. Antimicrobial Agents and Chemotherapy 61.

91. Hershberger E, Aeschlimann JR, Moldovan T, Rybak MJ. 1999. Evaluation of bactericidal activities of LY333328, vancomycin, teicoplanin, ampicillin-sulbactam, trovafloxacin, and RP59500 alone or in combination with rifampin or gentamicin against different strains of vancomycin-intermediate Staphylococcus aureus by time-kill curve methods. Antimicrob Agents Chemother 43:717-21.

92. Ho JL, Klempner MS. 1986. In vitro evaluation of clindamycin in combination with oxacillin, rifampin, or vancomycin against Staphylococcus aureus. Diagn Microbiol Infect Dis 4:133-8.

93. Hoogkamp-Korstanje JA. 1985. In vitro comparison of aminoglycoside activities and their synergistic action with piperacillin. Infection 13:39-42.

94. Hosgor-Limoncu M, Ermertcan S, Tasli H, Yurtman AN. 2008. Activity of amikacin, ertapenem, ciprofloxacin and levofloxacin alone and in combination against resistant nosocomial pathogens by time-kill. West Indian Med J 57:106-11.

95. Howe RA, Wootton M, Bennet PM, MacGowan AP, Walsh TR. 1999. Interactions between methicillin and vancomycin in methicillin-resistant Staphylococcus aureus strains displaying different phenotypes of vancomycin susceptibility. Journal of Clinical Microbiology 37:3068-3071.

96. Hutton MA, Sundaram A, Perri MB, Zervos MJ, Herc ES. 2020. Assessment of invitrosynergy of daptomycin or vancomycin plus ceftaroline for daptomycin non-susceptible Staphylococcus aureus. Diagn Microbiol Infect Dis 98:115126.

97. Jacqueline C, Caillon J, Le Mabecque V, Miegeville AF, Donnio PY, Bugnon D, Potel G. 2003. In vitro activity of linezolid alone and in combination with gentamicin, vancomycin or rifampicin against methicillin-resistant Staphylococcus aureus by time-kill curve methods. J Antimicrob Chemother 51:857-64.

98. Jacqueline C, Navas D, Batard E, Miegeville AF, Le Mabecque V, Kergueris MF, Bugnon D, Potel G, Caillon J. 2005. In vitro and in vivo synergistic activities of linezolid combined with subinhibitory concentrations of imipenem against methicillin-resistant Staphylococcus aureus. Antimicrob Agents Chemother 49:45-51.

99. Jacqueline C, Caillon J, Grossi O, Le Mabecque V, Miegeville AF, Bugnon D, Batard E, Potel G. 2006. In vitro and in vivo assessment of linezolid combined with ertapenem: a highly synergistic combination against methicillin-resistant Staphylococcus aureus. Antimicrob Agents Chemother 50:2547-9.

100. Jiang JH, Peleg AY. 2015. Daptomycin-Nonsusceptible Staphylococcus aureus: The Role of Combination Therapy with Daptomycin and Gentamicin. Genes 6:1256-1267.

101. Johnson DM, Fritsche TR, Sader HS, Jones RN. 2006. Evaluation of dalbavancin in combination with nine antimicrobial agents to detect enhanced or antagonistic interactions. Int J Antimicrob Agents 27:557-60.

102. Johnston BL, Kwok RY, Mulligan ME. 1987. In vitro activity of novobiocin and rifampin alone and in combination against oxacillin-resistant Staphylococcus aureus. Diagn Microbiol Infect Dis 8:137-47.

103. Jones RN, Packer RR, Barry AL, Badal RE, Thornsberry C, Baker C. 1979. Piperacillin (T-1220), a new semisynthetic penicillin. II. In vitro antimicrobial activity and synergy comparison with carbenicillin and gentamicin. J Antibiot (Tokyo) 32:29-35.

104. Joukhadar C, Pillai S, Wennersten C, Moellering RC, Jr., Eliopoulos GM. 2010. Lack of bactericidal antagonism or synergism in vitro between oxacillin and vancomycin against methicillin-susceptible strains of Staphylococcus aureus. Antimicrob Agents Chemother 54:773-7.

105. Just HM, Becker C, Bassler M, Daschner FD. 1984. In vitro combination effects of cefotetan with four aminoglycosides, piperacillin and mezlocillin on gram-positive and gram-negative nosocomial bacteria. Chemotherapy 30:387-91.

106. Kamble E, Sanghvi P, Pardesi K. 2022. Synergistic effect of antibiotic combinations on Staphylococcus aureus biofilms and their persister cell populations. Biofilm 4:100068.

107. Kang YR, Chung DR, Kim J, Baek JY, Kim SH, Ha YE, Kang CI, Peck KR, Song JH. 2016. In vitro synergistic effects of various combinations of vancomycin and non-beta-lactams against Staphylococcus aureus with reduced susceptibility to vancomycin. Diagn Microbiol Infect Dis 86:293-299.

108. Kang SL, Rybak MJ. 1997. In-vitro bactericidal activity of quinupristin/dalfopristin alone and in combination against resistant strains of Enterococcus species and Staphylococcus aureus. J Antimicrob Chemother 39:33-9.

109. Katou K, Nakamura A, Kato T, Tonegawa K, Kutsuna T, Niwa T, Morita H, Itoh M. 2005. Combined effects of panipenem and aminoglycosides on methicillin-resistant Staphylococcus aureus and Pseudomonas aeruginosa in vitro. Chemotherapy 51:387-391.

110. Kebriaei R, Rice SA, Singh NB, Stamper KC, Nguyen L, Sheikh Z, Rybak MJ. 2020. Combinations of (lipo)glycopeptides with beta-lactams against MRSA: susceptibility insights. Journal of Antimicrobial Chemotherapy 75:2894-2901.

111. Khasawneh FA, Ashcraft DS, Pankey GA. 2008. In vitro testing of daptomycin plus rifampin against methicillin-resistant Staphylococcus aureus resistant to rifampin. Saudi Med J 29:1726-9.

112. Kobayashi Y. 2005. Study of the synergism between carbapenems and vancomycin or teicoplanin against MRSA, focusing on S-4661, a carbapenem newly developed in Japan. J Infect Chemother 11:259-61.

113. Kobayashi S, Arai S, Hayashi S. 1989. In vitro and in vivo effects of combinations of cefotaxime or other beta-lactams with rolitetracycline on methicillin-resistant Staphylococcus aureus. Jpn J Antibiot 42:1208-15.

114. Komatsuzawa H, Suzuki J, Sugai M, Miyake Y, Suginaka H. 1994. Effect of combination of oxacillin and non-beta-lactam antibiotics on methicillin-resistant Staphylococcus aureus. J Antimicrob Chemother 33:1155-63.

115. Kondo M, Tsuchiya K. 1981. Effect of combination of cefsulodin and mecillinam. J Antibiot (Tokyo) 34:727-38.

116. Kussmann M, Obermueller M, Karer M, Kriz R, Chen RY, Hohl L, Schneider L, Burgmann H, Traby L, Vossen MG. 2021. Synergistic Effect of Cefazolin Plus Fosfomycin Against Staphylococcus aureus in vitro and in vivo in an Experimental Galleria mellonella Model. Front Pharmacol 12:685807.

117. Lai CC, Chen CC, Chuang YC, Tang HJ. 2017. Combination of cephalosporins with vancomycin or teicoplanin enhances antibacterial effect of glycopeptides against heterogeneous vancomycin-intermediate Staphylococcus aureus (hVISA) and VISA. Sci Rep 7:41758.

118. Lai CC, Chen CC, Lu YC, Lin TP, Chen HJ, Su BA, Chao CM, Chuang YC, Tang HJ. 2019. The Potential Role of Sulbactam and Cephalosporins Plus Daptomycin Against Daptomycin-Nonsusceptible VISA and H-VISA Isolates: An in Vitro Study. Antibiotics (Basel) 8.

119. Lee SH, Teo J, Heng D, Ng WK, Chan HK, Tan RBH. 2013. Synergistic combination dry powders for inhaled antimicrobial therapy: Formulation, characterization and in vitro evaluation. European Journal of Pharmaceutics and Biopharmaceutics 83:275-284.

120. Lee YC, Chen PY, Wang JT, Chang SC. 2019. A study on combination of daptomycin with selected antimicrobial agents: in vitro synergistic effect of MIC value of 1 mg/L against MRSA strains. BMC Pharmacol Toxicol 20:25.

121. Lee JY, Oh WS, Ko KS, Heo ST, Moon CS, Ki HK, Kiem S, Peck KR, Song JH. 2006. Synergy of arbekacin-based combinations against vancomycin hetero-intermediate Staphylococcus aureus. J Korean Med Sci 21:188-92.

122. Leonard SN. 2012. Synergy between vancomycin and nafcillin against Staphylococcus aureus in an in vitro pharmacokinetic/pharmacodynamic model. PLoS One 7:e42103.

123. Leonard SN, Rolek KM. 2013. Evaluation of the combination of daptomycin and nafcillin against vancomycin-intermediate Staphylococcus aureus. J Antimicrob Chemother 68:644-7.

124. Leonard SN, Cheung CM, Rybak MJ. 2008. Activities of ceftobiprole, linezolid, vancomycin, and daptomycin against community-associated and hospital-associated methicillin-resistant Staphylococcus aureus. Antimicrob Agents Chemother 52:2974-6.

125. Leonard SN, Kaatz GW, Rucker LR, Rybak MJ. 2008. Synergy between gemifloxacin and trimethoprim/sulfamethoxazole against community-associated methicillin-resistant Staphylococcus aureus. J Antimicrob Chemother 62:1305-10.

126. Leonard SN, Supple ME, Gandhi RG, Patel MD. 2013. Comparative activities of telavancin combined with nafcillin, imipenem, and gentamicin against Staphylococcus aureus. Antimicrob Agents Chemother 57:2678-83.

127. Li L, Chen H, Liu Y, Xu S, Wu M, Liu Z, Qi C, Zhang G, Li J, Huang X. 2020. Synergistic effect of linezolid with fosfomycin against Staphylococcus aureus in vitro and in an experimental Galleria mellonella model. J Microbiol Immunol Infect 53:731-738.

128. Liao CH, Chen SY, Huang YT, Tsai HY, Hsueh PR. 2017. Comparison of in vitro synergy of various β-lactams with vancomycin against methicillin-resistant Staphylococcus aureus. J Infect 74:324-325.

129. Lin G, Pankuch G, Appelbaum PC, Kosowska-Shick K. 2014. Antistaphylococcal activity of oritavancin and its synergistic effect in combination with other antimicrobial agents. Antimicrob Agents Chemother 58:6251-4.

130. Lin G, Ednie LM, Appelbaum PC. 2010. Antistaphylococcal activity of ACHN-490 tested alone and in combination with other agents by time-kill assay. Antimicrob Agents Chemother 54:2258-61.

131. Lin G, Pankuch GA, Ednie LM, Appelbaum PC. 2010. Antistaphylococcal activities of telavancin tested alone and in combination by time-kill assay. Antimicrob Agents Chemother 54:2201-5.

132. Liu X, Pai PJ, Zhang W, Hu Y, Dong X, Qian PY, Chen D, Lam H. 2016. Proteomic response of methicillin-resistant S. aureus to a synergistic antibacterial drug combination: a novel erythromycin derivative and oxacillin. Sci Rep 6:19841.

133. Lozniewski A, Lion C, Mory F, Weber M. 2001. In vitro synergy between cefepime and vancomycin against methicillin-susceptible and -resistant Staphylococcus aureus and Staphylococcus epidermidis. J Antimicrob Chemother 47:83-6.

134. Machka K, Dietz R. 1983. Comparative synergistic activity of ceftriaxone-piperacillin versus ceftriaxone-netilmicin. Eur J Clin Microbiol 2:496-500.

135. Mackay ML, Milne K, Gould IM. 2000. Comparison of methods for assessing synergic antibiotic interactions. Int J Antimicrob Agents 15:125-9.

136. Maduri Traczewski M, Goldmann DA, Murphy P. 1983. In vitro activity of rifampin in combination with oxacillin against Staphylococcus aureus. Antimicrob Agents Chemother 23:571-6.

137. Marchese A, Saverino D, Debbia EA, Pesce A, Schito GC. 1995. Antistaphylococcal activity of cefdinir, a new oral third-generation cephalosporin, alone and in combination with other antibiotics, at supra- and sub-MIC levels. J Antimicrob Chemother 35:53-66.

138. Matsuda K, Nakamura K, Adachi Y, Inoue M, Kawakami M. 1995. Autolysis of methicillin-resistant Staphylococcus aureus is involved in synergism between imipenem and cefotiam. Antimicrob Agents Chemother 39:2631-4.

139. Matsumoto T, Kubo S, Haraoka M, Takahashi K, Tanaka M, Sakumoto M, Kumazawa J. 1993. Combination chemotherapy for infections due to methicillin-resistant Staphylococcus aureus with combination therapy by cefuzonam and fosfomycin or minocycline in the urologic field. Clin Ther 15:819-28.

140. McConeghy KW, LaPlante KL. 2010. In vitro activity of tigecycline in combination with gentamicin against biofilm-forming Staphylococcus aureus. Diagnostic Microbiology and Infectious Disease 68:1-6.

141. Mehta S, Singh C, Plata KB, Chanda PK, Paul A, Riosa S, Rosato RR, Rosato AE. 2012. beta-Lactams Increase the Antibacterial Activity of Daptomycin against Clinical Methicillin-Resistant Staphylococcus aureus Strains and Prevent Selection of Daptomycin-Resistant Derivatives. Antimicrobial Agents and Chemotherapy 56:6192-6200.

142. Mercier RC, Houlihan HH, Rybak MJ. 1997. Pharmacodynamic evaluation of a new glycopeptide, LY333328, and in vitro activity against Staphylococcus aureus and Enterococcus faecium. Antimicrob Agents Chemother 41:1307-12.

143. Mercier RC, Kennedy C, Meadows C. 2002. Antimicrobial activity of tigecycline (GAR-936) against Enterococcus faecium and Staphylococcus aureus used alone and in combination. Pharmacotherapy 22:1517-23.

144. Meyers BR, Srulevitch ES, Hirschman SZ. 1985. Comparative in vitro activity of coumermycin against methicillin-resistant Staphylococcus aureus. Antimicrob Agents Chemother 28:706-7.

145. Mir, a-Novales G, Leaños-Mir, a BE, Vilchis-Pérez M, Solórzano-Santos F. 2006. In vitro activity effects of combinations of cephalothin, dicloxacillin, imipenem, vancomycin and amikacin against methicillin-resistant Staphylococcus spp. strains. Ann Clin Microbiol Antimicrob 5:25.

146. Miró JM, García-de-la-Mària C, Armero Y, Soy D, Moreno A, del Río A, Almela M, Sarasa M, Mestres CA, Gatell JM, Jiménez de Anta MT, Marco F. 2009. Addition of gentamicin or rifampin does not enhance the effectiveness of daptomycin in treatment of experimental endocarditis due to methicillin-resistant Staphylococcus aureus. Antimicrob Agents Chemother 53:4172-7.

147. Miro JM, Entenza JM, del Rio A, Velasco M, Castaneda X, de la Maria CG, Giddey M, Armero Y, Pericas JM, Cervera C, Mestres CA, Almela M, Falces C, Marco F, Moreillon P, Moreno A, Hosp Clinic Expt E. 2012. High-Dose Daptomycin plus Fosfomycin Is Safe and Effective in Treating Methicillin-Susceptible and Methicillin-Resistant Staphylococcus aureus Endocarditis. Antimicrobial Agents and Chemotherapy 56:4511-4515.

148. Mirza HC, Öğüç Şanlı Ö. 2024. Evaluation of in vitro activity of ceftaroline, ceftobiprole and their combination with trimethoprim/sulfamethoxazole against MRSA isolates: a two center study. J Chemother doi:10.1080/1120009x.2024.2316539:1-8.

149. Mohammadi-Berenjestanaki H, Khori V, Shirzad-Aski H, Ghaemi EA. 2020. In Vitro Synergistic Effect of Vancomycin and Some Antibacterial Agents Against Clinical Methicillin-Resistant and Sensitive Staphylococcus aureus Isolates. Microb Drug Resist 26:218-226.

150. Moody JA, Peterson LR, Gerding DN. 1985. In vitro activity of ciprofloxacin combined with azlocillin. Antimicrob Agents Chemother 28:849-50.

151. Mulazimoglu L, Drenning SD, Muder RR. 1996. Vancomycin-gentamicin synergism revisited: effect of gentamicin susceptibility of methicillin-resistant Staphylococcus aureus. Antimicrob Agents Chemother 40:1534-5.

152. Mulazimoglu L, Drenning SD, Yu VL. 1996. In vitro activities of two novel oxazolidinones (U100592 and U100766), a new fluoroquinolone (trovafloxacin), and dalfopristin-quinupristin against Staphylococcus aureus and Staphylococcus epidermidis. Antimicrob Agents Chemother 40:2428-30.

153. Neu HC, Chin NX, Labthavikul P. 1984. Antibacterial activity of coumermycin alone and in combination with other antibiotics. Antimicrob Agents Chemother 25:687-9.

154. Neu HC, Labthavikul P. 1983. In vitro activity of teichomycin compared with those of other antibiotics. Antimicrob Agents Chemother 24:425-8.

155. Norden CW. 1975. Experimental osteomyelitis. IV. Therapeutic trials with rifampin alone and in combination with gentamicin, sisomicin, and cephalothin. J Infect Dis 132:493-9.

156. Norden CW. 1978. Experimental osteomyelitis. V. Therapeutic trials with oxacillin and sisomicin alone and in combination. J Infect Dis 137:155-60.

157. Oka S, Goto M, Kaji Y, Kimura S, Matsuda K, Asahi Y, Sanada M, Nakagawa S, Inoue M, Shimada K. 1993. SYNERGIC ACTIVITY OF IMIPENEM CILASTATIN COMBINED WITH CEFOTIAM AGAINST METHICILLIN-RESISTANT STAPHYLOCOCCUS-AUREUS. J Antimicrob Chemother 31:533-541.

158. Osburne MS, Murphy CK, Rothstein DM. 2006. Enhanced activity of rifalazil in combination with levofloxacin, linezolid, or mupirocin against Staphylococcus aureus in vitro. J Antibiot (Tokyo) 59:303-8.

159. Osburne MS, Rothstein DM, Farquhar R, Murphy CK. 2006. In vitro time-kill activities of rifalazil, alone and in combination with vancomycin, against logarithmic and stationary cultures of Staphylococcus aureus. J Antibiot (Tokyo) 59:80-5.

160. Pachón-Ibáñez ME, Ribes S, Domínguez MA, Fernández R, Tubau F, Ariza J, Gudiol F, Cabellos C. 2011. Efficacy of fosfomycin and its combination with linezolid, vancomycin and imipenem in an experimental peritonitis model caused by a Staphylococcus aureus strain with reduced susceptibility to vancomycin. Eur J Clin Microbiol Infect Dis 30:89-95.

161. Palmer SM, Rybak MJ. 1997. An evaluation of the bactericidal activity of ampicillin/sulbactam, piperacillin/tazobactam, imipenem or nafcillin alone and in combination with vancomycin against methicillin-resistant Staphylococcus aureus (MRSA) in time-kill curves with infected fibrin clots. J Antimicrob Chemother 39:515-8.

162. Park B, Min YH. 2020. In vitro synergistic effect of retapamulin with erythromycin and quinupristin against Enterococcus faecalis. J Antibiot (Tokyo) 73:630-635.

163. Patel JA, Pachucki CT, Lentino JR. 1993. SYNERGY OF LEVOFLOXACIN (L-OFLOXACIN) AND OXACILLIN AGAINST QUINOLONE-RESISTANT STAPHYLOCOCCUS-AUREUS, MEASURED BY THE TIME-KILL METHOD. Antimicrob Agents Chemother 37:339-341.

164. Pavie J, Lefort A, Zarrouk V, Chau F, Garry L, Leclercq R, Fantin B. 2002. Efficacies of quinupristin-dalfopristin combined with vancomycin in vitro and in experimental endocarditis due to methicillin-resistant Staphylococcus aureus in relation to cross-resistance to macrolides, lincosamides, and streptogramin B- type antibiotics. Antimicrob Agents Chemother 46:3061-4.

165. Petersen PJ, Labthavikul P, Jones CH, Bradford PA. 2006. In vitro antibacterial activities of tigecycline in combination with other antimicrobial agents determined by chequerboard and time-kill kinetic analysis. Journal of Antimicrobial Chemotherapy 57:573-576.

166. Rand KH, Houck HJ. 2004. Synergy of daptomycin with oxacillin and other beta-lactams against methicillin-resistant Staphylococcus aureus. Antimicrob Agents Chemother 48:2871-5.

167. Rand KH, Brown P. 1995. CONCENTRATION-DEPENDENT SYNERGY AND ANTAGONISM BETWEEN CEFOPERAZONE AND IMIPENEM AGAINST METHICILLIN-RESISTANT STAPHYLOCOCCUS-AUREUS. Antimicrobial Agents and Chemotherapy 39:1173-1177.

168. Raymond J, Vedel G, Bergeret M. 1998. In vitro bactericidal activity of cefpirome in combination with vancomycin against Staphylococcus aureus and coagulase-negative Staphylococcus. Diagn Microbiol Infect Dis 31:481-3.

169. Renneberg J, Karlsson E, Nilsson B, Walder M. 1993. Interactions of drugs acting against Staphylococcus aureus in vitro and in a mouse model. J Infect 26:265-77.

170. Ribes S, Pachón-Ibáñez ME, Domínguez MA, Fernández R, Tubau F, Ariza J, Gudiol F, Cabellos C. 2010. In vitro and in vivo activities of linezolid alone and combined with vancomycin and imipenem against Staphylococcus aureus with reduced susceptibility to glycopeptides. Eur J Clin Microbiol Infect Dis 29:1361-7.

171. Rochon-Edouard S, Pestel-Caron M, Lemeland JF, Caron F. 2000. In vitro synergistic effects of double and triple combinations of beta-lactams, vancomycin, and netilmicin against methicillin-resistant Staphylococcus aureus strains. Antimicrob Agents Chemother 44:3055-60.

172. Rohner P, Herter C, Auckenthaler R, Pechère JC, Waldvogel FA, Lew DP. 1989. Synergistic effect of quinolones and oxacillin on methicillin-resistant Staphylococcus species. Antimicrob Agents Chemother 33:2037-41.

173. Rose WE, Berti AD, Hatch JB, Maki DG. 2013. Relationship of in vitro synergy and treatment outcome with daptomycin plus rifampin in patients with invasive methicillin-resistant Staphylococcus aureus infections. Antimicrob Agents Chemother 57:3450-2.

174. Rose WE, Poppens PT. 2009. Impact of biofilm on the in vitro activity of vancomycin alone and in combination with tigecycline and rifampicin against Staphylococcus aureus. J Antimicrob Chemother 63:485-8.

175. Sabath LD, Steinhauer BW, Finland M. 1963. Combined action of penicillin G with methicillin or oxacillin against Staphylococcus aureus. N Engl J Med 268:284-6.

176. Sahuquillo Arce JM, Colombo Gainza E, Gil Brusola A, Ortiz Estévez R, Cantón E, Gobernado M. 2006. In vitro activity of linezolid in combination with doxycycline, fosfomycin, levofloxacin, rifampicin and vancomycin against methicillin-susceptible Staphylococcus aureus. Rev Esp Quimioter 19:252-7.

177. Sakoulas G, Moise PA, Casapao AM, Nonejuie P, Olson J, Okumura CY, Rybak MJ, Kullar R, Dhand A, Rose WE, Goff DA, Bressler AM, Lee Y, Pogliano J, Johns S, Kaatz GW, Ebright JR, Nizet V. 2014. Antimicrobial salvage therapy for persistent staphylococcal bacteremia using daptomycin plus ceftaroline. Clin Ther 36:1317-33.

178. Sakoulas G, Olson J, Yim J, Singh NB, Kumaraswamy M, Quach DT, Rybak MJ, Pogliano J, Nizet V. 2016. Cefazolin and Ertapenem, a Synergistic Combination Used To Clear Persistent Staphylococcus aureus Bacteremia. Antimicrob Agents Chemother 60:6609-6618.

179. Sambatakou H, Giamarellos-Bourboulis EJ, Grecka P, Chryssouli Z, Giamarellou H. 1998. In-vitro activity and killing effect of quinupristin/dalfopristin (RP59500) on nosocomial Staphylococcus aureus and interactions with rifampicin and ciprofloxacin against methicillin-resistant isolates. J Antimicrob Chemother 41:349-55.

180. Sanal L, Yilmaz N, Uludag H, Ozturk R, Sen S, Cesur S. 2018. Detection of Synergistic Antimicrobial Activities of Ceftaroline, Telavancin, Daptomycin, and Vancomycin Against Methicillin-Resistant Staphylococcus aureus Strains in Intensive Care. Jundishapur Journal of Microbiology 11.

181. Santimaleeworagun W, Jitwasinkul T, Preechachuawong P, Samret W. 2020. MONO SULFAMETHOXAZOLE/TRIMETHOPRIM AND VANCOMYCIN COMBINATION ANTIMICROBIAL ACTIVITY AGAINST METHICILLIN- RESISTANT STAPHYLOCOCCUS AUREUS. Southeast Asian Journal of Tropical Medicine and Public Health 51:115-123.

182. Saravolatz LD, Pawlak J. 2022. In vitro activity of fosfomycin alone and in combination against Staphylococcus aureus with reduced susceptibility or resistance to methicillin, vancomycin, daptomycin or linezolid. J Antimicrob Chemother 78:238-241.

183. Saverino D, Debbia EA, Pesce A, Lepore AM, Schito GC. 1992. Antibacterial profile of flurithromycin, a new macrolide. J Antimicrob Chemother 30:261-72.

184. Seibert G, Isert D, Klesel N, Limbert M, Markus A, Schrinner E. 1992. The in-vitro antibacterial activity of a combination of cefpirome or cefoperazone with vancomycin against enterococci and Staphylococcus aureus. J Antimicrob Chemother 29:25-30.

185. Shafiq I, Bulman ZP, Spitznogle SL, Osorio JE, Reilly IS, Lesse AJ, Parameswaran GI, Mergenhagen KA, Tsuji BT. 2017. A combination of ceftaroline and daptomycin has synergistic and bactericidal activity in vitro against daptomycin nonsusceptible methicillin-resistant Staphylococcus aureus (MRSA). Infect Dis (Lond) 49:410-416.

186. Sharma AD, Gutheil WG. 2023. Synergistic Combinations of FDA-Approved Drugs with Ceftobiprole against Methicillin-Resistant Staphylococcus aureus. Microbiol Spectr 11:e0372622.

187. Shelburne SA, Musher DM, Hulten K, Ceasar H, Lu MY, Bhaila I, Hamill RJ. 2004. In vitro killing of community-associated methicillin-resistant Staphylococcus aureus with drug combinations. Antimicrob Agents Chemother 48:4016-9.

188. Shi J, Mao NF, Wang L, Zhang HB, Chen Q, Liu H, Tang X, Jin T, Zhu CT, Li FB, Sun LH, Xu XM, Xu YQ. 2014. Efficacy of combined vancomycin and fosfomycin against methicillin-resistant Staphylococcus aureus in biofilms in vivo. PLoS One 9:e113133.

189. Silva LV, Araújo MT, Santos KR, Nunes AP. 2011. Evaluation of the synergistic potential of vancomycin combined with other antimicrobial agents against methicillin-resistant Staphylococcus aureus and coagulase-negative Staphylococcus spp strains. Mem Inst Oswaldo Cruz 106:44-50.

190. Silvestri C, Cirioni O, Arzeni D, Ghiselli R, Simonetti O, Orlando F, Ganzetti G, Staffolani S, Brescini L, Provinciali M, Offidani A, Guerrieri M, Giacometti A. 2012. In vitro activity and in vivo efficacy of tigecycline alone and in combination with daptomycin and rifampin against Gram-positive cocci isolated from surgical wound infection. Eur J Clin Microbiol Infect Dis 31:1759-64.

191. Simon C, Simon M. 1991. In vitro activity of flomoxef and cefazolin in combination with vancomycin. Infection 19:S276-8.

192. Simonetti O, Morroni G, Ghiselli R, Orlando F, Brenciani A, Xhuvelaj L, Provinciali M, Offidani A, Guerrieri M, Giacometti A, Cirioni O. 2018. In vitro and in vivo activity of fosfomycin alone and in combination with rifampin and tigecycline against Gram-positive cocci isolated from surgical wound infections. Journal of Medical Microbiology 67:139-143.

193. Singh SR, Bacon AE, 3rd, Young DC, Couch KA. 2009. In vitro 24-hour time-kill studies of vancomycin and linezolid in combination versus methicillin-resistant Staphylococcus aureus. Antimicrob Agents Chemother 53:4495-7.

194. Smelter D, Hayney M, Sakoulas G, Rose W. 2022. Is the Success of Cefazolin plus Ertapenem in Methicillin-Susceptible Staphylococcus aureus Bacteremia Based on Release of Interleukin-1 Beta? Antimicrob Agents Chemother 66:e0216621.

195. Smith SM, Eng RH. 1985. Activity of ciprofloxacin against methicillin-resistant Staphylococcus aureus. Antimicrob Agents Chemother 27:688-91.

196. Smith SM, Eng RH, Berman E. 1986. The effect of ciprofloxacin on methicillin-resistant Staphylococcus aureus. J Antimicrob Chemother 17:287-95.

197. Smith JR, Yim J, Raut A, Rybak MJ. 2016. Oritavancin Combinations with β-Lactams against Multidrug-Resistant Staphylococcus aureus and Vancomycin-Resistant Enterococci. Antimicrob Agents Chemother 60:2352-8.

198. Snydman DR, McDermott LA, Jacobus NV. 2005. Evaluation of in vitro interaction of daptomycin with gentamicin or beta-lactam antibiotics against Staphylococcus aureus and Enterococci by FIC index and timed-kill curves. J Chemother 17:614-21.

199. Soares LA, Trabulsi LR. 1979. Synergistic action between sisomicin and mezlocillin against gram-negative bacteria and Staphylococcus aureus. Arzneimittelforschung 29:1934-7.

200. Srisrattakarn A, Chaiyapoke C, Booncharoen S, Wongthong S, Chanawong A, Tippayawat P, Tavichakorntrakool R, Lulitanond A. 2021. Synergistic effect of vancomycin combined with cefotaxime, imipenem, or meropenem against Staphylococcus aureus with reduced susceptibility to vancomycin. Turk J Med Sci 51:2150-2158.

201. Stefani S, Debbia E, Schito GC, Nicoletti G. 1988. In vitro interaction between cefotetan and aminoglycosides on Staphylococcus aureus and coagulase negative staphylococci, both methicillin-susceptible and -resistant. Chemioterapia 7:151-5.

202. Steigbigel RT, Greenman RL, Remington JS. 1975. Antibiotic combinations in the treatment of experimental Staphylococcus aureus infection. J Infect Dis 131:245-51.

203. Stein C, Makarewicz O, Forstner C, Weis S, Hagel S, Löffler B, Pletz MW. 2016. Should daptomycin-rifampin combinations for MSSA/MRSA isolates be avoided because of antagonism? Infection 44:499-504.

204. Stutman HR, Welch DF, Scribner RK, Marks MI. 1984. In vitro antimicrobial activity of aztreonam alone and in combination against bacterial isolates from pediatric patients. Antimicrob Agents Chemother 25:212-5.

205. Sueke H, Kaye SB, Neal T, Hall A, Tuft S, Parry CM. 2010. An In Vitro Investigation of Synergy or Antagonism between Antimicrobial Combinations against Isolates from Bacterial Keratitis. Invest Ophthalmol Vis Sci 51:4151-4155.

206. Sugiura A, Jono K, Kono T, Higashide E. 1991. The effect of combinations of cefotiam and other antibiotics on methicillin-resistant Staphylococcus aureus in vitro. J Antimicrob Chemother 28:707-17.

207. Sumita Y, Mitsuhashi S. 1991. In vitro synergistic activity between meropenem and other beta-lactams against methicillin-resistant Staphylococcus aureus. Eur J Clin Microbiol Infect Dis 10:77-84.

208. Sumon ZE, Berenson CS, Sellick JA, Bulman ZP, Tsuji BT, Mergenhagen KA. 2019. Successful cure of daptomycin-non-susceptible, vancomycin-intermediate Staphylococcus aureus prosthetic aortic valve endocarditis directed by synergistic in vitro time-kill study. Infect Dis (Lond) 51:287-292.

209. Sun CG, Falagas ME, Wang R, Karageorgopoulos DE, Yu XH, Liu YN, Cai Y, Liang BB, Song XJ, Liu ZY. 2011. In vitro activity of minocycline combined with fosfomycin against clinical isolates of methicillin-resistant Staphylococcus aureus. Journal of Antibiotics 64:559-562.

210. Sweeney MT, Zurenko GE. 2003. In vitro activities of linezolid combined with other antimicrobial agents against Staphylococci, Enterococci, Pneumococci, and selected gram-negative organisms. Antimicrob Agents Chemother 47:1902-6.

211. Sy CL, Huang TS, Chen CS, Chen YS, Tsai HC, Wann SR, Wu KS, Chen JK, Lee SS, Liu YC. 2016. Synergy of β-Lactams with Vancomycin against Methicillin-Resistant Staphylococcus aureus: Correlation of Disk Diffusion and Checkerboard Methods. J Clin Microbiol 54:565-8.

212. Tang HJ, Lai CC, Chen CC, Zhang CC, Weng TC, Yu WL, Chen HJ, Chiu YH, Ko WC, Chuang YC. 2017. Cephalosporin-Glycopeptide Combinations for Use against Clinical Methicillin-Resistant Staphylococcus aureus Isolates: Enhanced In vitro Antibacterial Activity. Front Microbiol 8:884.

213. Thamlikitkul V. 1991. Synergy study of vancomycin or teicoplanin plus gentamicin against enterococci, Staphylococcus aureus and coagulase-negative staphylococci by time-kill method. J Med Assoc Thai 74:669-74.

214. Totsuka K, Shiseki M, Kikuchi K, Matsui Y. 1999. Combined effects of vancomycin and imipenem against methicillin-resistant Staphylococcus aureus (MRSA) in vitro and in vivo. J Antimicrob Chemother 44:455-60.

215. Toyokawa M, Asari S, Nishi I, Horikawa M, Tsukamoto H, Sunada A, Ueda A, Iwatani Y. 2003. In vitro combined effects of cefozopran/teicoplanin and cefozopran/vancomycin on methicillin-resistant Staphylococcus aureus. J Chemother 15:31-6.

216. Traub WH, Spohr M, Bauer D. 1986. In vitro additive effect of imipenem combined with vancomycin against multiple-drug resistant, coagulase-negative Staphylococci. Zentralbl Bakteriol Mikrobiol Hyg A 262:361-9.

217. Tran N, Rybak MJ. 2018. β-Lactam Combinations with Vancomycin Show Synergistic Activity against Vancomycin-Susceptible Staphylococcus aureus, Vancomycin-Intermediate S. aureus (VISA), and Heterogeneous VISA. Antimicrob Agents Chemother 62.

218. Tsai CE, Yang CJ, Chuang YC, Wang JT, Sheng WH, Chen YC, Chang SC. 2022. Evaluation of the synergistic effect of ceftaroline against methicillin-resistant Staphylococcus aureus. Int J Infect Dis 122:230-236.

219. Tsuji BT, Rybak MJ. 2006. Etest synergy testing of clinical isolates of Staphylococcus aureus demonstrating heterogeneous resistance to vancomycin. Diagn Microbiol Infect Dis 54:73-7.

220. Tuazon CU, Miller H. 1984. Comparative in vitro activities of teichomycin and vancomycin alone and in combination with rifampin and aminoglycosides against staphylococci and enterococci. Antimicrob Agents Chemother 25:411-2.

221. Uete T, Matsuo K. 1995. Synergistic enhancement of in vitro antimicrobial activity of imipenem and cefazolin, cephalothin, cefotiam, cefamandole or cefoperazone in combination against methicillin-sensitive and -resistant Staphylococcus aureus. Jpn J Antibiot 48:402-8.

222. Uete T, Matsuo K. 1995. Synergistic enhancement of in vitro antimicrobial activity of cefmetazole and cefazolin, cefotiam, cefamandole or cefoperazone in combination against methicillin-sensitive and -resistant Staphylococcus aureus. I. Effect of NaCl. Jpn J Antibiot 48:553-62.

223. Ulloa ER, Singh KV, Geriak M, Haddad F, Murray BE, Nizet V, Sakoulas G. 2020. Cefazolin and Ertapenem Salvage Therapy Rapidly Clears Persistent Methicillin-Susceptible Staphylococcus aureus Bacteremia. Clin Infect Dis 71:1413-1418.

224. Utsui Y, Ohya S, Magaribuchi T, Tajima M, Yokota T. 1986. Antibacterial activity of cefmetazole alone and in combination with fosfomycin against methicillin- and cephem-resistant Staphylococcus aureus. Antimicrob Agents Chemother 30:917-22.

225. Valderrama MJ, Alfaro M, Rodríguez-Avial I, Baos E, Rodríguez-Avial C, Culebras E. 2020. Synergy of Linezolid with Several Antimicrobial Agents against Linezolid-Methicillin-Resistant Staphylococcal Strains. Antibiotics (Basel) 9.

226. Van der Auwera P, Joly P. 1987. Comparative in-vitro activities of teicoplanin, vancomycin, coumermycin and ciprofloxacin, alone and in combination with rifampicin or LM 427, against Staphylococcus aureus. J Antimicrob Chemother 19:313-20.

227. Van der Auwera P, Klastersky J. 1986. In vitro activity of coumermycin alone or in combination against Staphylococcus aureus and Staphylococcus epidermidis. Drugs Exp Clin Res 12:307-11.

228. van der Auwera P, Vandermies A, Grenier P, Klastersky J. 1987. Comparative in vitro activity of CI934, a new fluoroquinolone, alone and in combination with coumermycin, against gram-positive bacteria. Drugs Exp Clin Res 13:125-32.

229. Verbist L, Verhaegen J. 1984. Effect of temocillin in combination with other beta-lactam antibiotics. Antimicrob Agents Chemother 25:142-4.

230. Vidaillac C, Leonard SN, Rybak MJ. 2010. In vitro evaluation of ceftaroline alone and in combination with tobramycin against hospital-acquired meticillin-resistant Staphylococcus aureus (HA-MRSA) isolates. Int J Antimicrob Agents 35:527-30.

231. Vouillamoz J, Entenza JM, Féger C, Glauser MP, Moreillon P. 2000. Quinupristin-dalfopristin combined with beta-lactams for treatment of experimental endocarditis due to Staphylococcus aureus constitutively resistant to macrolide-lincosamide-streptogramin B antibiotics. Antimicrob Agents Chemother 44:1789-95.

232. Walsh TJ, Auger F, Tatem BA, Hansen SL, Standiford HC. 1986. Novobiocin and rifampicin in combination against methicillin-resistant Staphylococcus aureus: an in-vitro comparison with vancomycin plus rifampicin. J Antimicrob Chemother 17:75-82.

233. Watanakunakorm C, Glotzbecker C. 1974. Enhancement of the effects of anti-staphylococcal antibiotics by aminoglycosides. Antimicrob Agents Chemother 6:802-6.

234. Watanakunakorn C, Glotzbecker C. 1977. Enhancement of antistaphylococcal activity of nafcillin and oxacillin by sisomicin and netilmicin. Antimicrob Agents Chemother 12:346-8.

235. Watanakunakorn C, Glotzbecker C. 1979. In vitro activity of carbenicillin, ticarcillin, aminoglycosides and combinations against Staphylococcus aureus. J Antimicrob Chemother 5:151-8.

236. Watanakunakorn C, Glotzbecker C. 1980. Effects of combinations of clindamycin with gentamicin, tobramycin, and amikacin against Staphylococcus aureus. J Antimicrob Chemother 6:785-91.

237. Watanakunakorn C, Guerriero JC. 1981. Interaction between vancomycin and rifampin against Staphylococcus aureus. Antimicrob Agents Chemother 19:1089-91.

238. Watanakunakorn C, Tisone JC. 1982. Synergism between vancomycin and gentamicin or tobramycin for methicillin-susceptible and methicillin-resistant Staphylococcus aureus strains. Antimicrob Agents Chemother 22:903-5.

239. Watanakunakorn C, Tisone JC. 1982. Antagonism between nafcillin or oxacillin and rifampin against Staphylococcus aureus. Antimicrob Agents Chemother 22:920-2.

240. Wattanapaisal D, Pattharachayakul S, Santimaleeworagun W, Jaruratanasirikul S, Hortiwakul T, Ingviriya N, Singkhamanan K, Garey KW. 2022. EFFICACY OF FUSIDIC ACID ALONE AND IN COMBINATION WITH OTHER ORAL ANTIMICROBIAL AGENTS AGAINST CLINICAL METHICILLIN-RESISTANT <i>STAPHYLOCOCCUS AUREUS </i>ISOLATES<i> IN VITRO</i>. Southeast Asian Journal of Tropical Medicine and Public Health 53:357-367.

241. Weber P, Boussougant Y, Ichou F, Dutoit C, Carbon C. 1987. Bactericidal effect of ofloxacin alone and combined with fosfomycin or vancomycin against Staphylococcus aureus in vitro and in sera from volunteers. J Antimicrob Chemother 20:839-47.

242. Welch WD, Bawdon RE, Luttrell B, Goodson S. 1984. Interaction of clindamycin and cefpimizole (U63196E) in vitro against aerobic gram-negative rods and aerobic gram-positive cocci. J Antimicrob Chemother 14:553-6.

243. Werth BJ, Sakoulas G, Rose WE, Pogliano J, Tewhey R, Rybak MJ. 2013. Ceftaroline increases membrane binding and enhances the activity of daptomycin against daptomycin-nonsusceptible vancomycin-intermediate Staphylococcus aureus in a pharmacokinetic/pharmacodynamic model. Antimicrob Agents Chemother 57:66-73.

244. Werth BJ. 2017. Exploring the pharmacodynamic interactions between tedizolid and other orally bioavailable antimicrobials against Staphylococcus aureus and Staphylococcus epidermidis. J Antimicrob Chemother 72:1410-1414.

245. White RL, Burgess DS, Manduru M, Bosso JA. 1996. Comparison of three different in vitro methods of detecting synergy: time-kill, checkerboard, and E test. Antimicrob Agents Chemother 40:1914-8.

246. Wicha SG, Kees MG, Kuss J, Kloft C. 2015. Pharmacodynamic and response surface analysis of linezolid or vancomycin combined with meropenem against Staphylococcus aureus. Pharm Res 32:2410-8.

247. Wise R, Ashby JP, Andrews JM. 1989. The antibacterial activity of meropenem in combination with gentamicin or vancomycin. J Antimicrob Chemother 24:233-8.

248. Wise R, Gillett AP, Andrews JM. 1979. The in vitro activity of mezlocillin when combined with cefoxitin or clavulanic acid. J Antimicrob Chemother 5:301-6.

249. Xhemali X, Smith JR, Kebriaei R, Rice SA, Stamper KC, Compton M, Singh NB, Jahanbakhsh S, Rybak MJ. 2019. Evaluation of dalbavancin alone and in combination with beta-lactam antibiotics against resistant phenotypes of Staphylococcus aureus. Journal of Antimicrobial Chemotherapy 74:82-86.

250. Xie N, Jiang L, Chen M, Zhang G, Liu Y, Li J, Huang X. 2021. In vitro and in vivo Antibacterial Activity of Linezolid Plus Fosfomycin Against Staphylococcus aureus with Resistance to One Drug. Infect Drug Resist 14:639-649.

251. Xu X, Xu L, Yuan G, Wang Y, Qu Y, Zhou M. 2018. Synergistic combination of two antimicrobial agents closing each other's mutant selection windows to prevent antimicrobial resistance. Sci Rep 8:7237.

252. Xu-hong Y, Falagas ME, Dong W, Karageorgopoulos DE, De-feng L, Rui W. 2014. In vitro activity of fosfomycin in combination with linezolid against clinical isolates of methicillin-resistant Staphylococcus aureus. J Antibiot (Tokyo) 67:369-71.

253. Yang SJ, Xiong YQ, Boyle-Vavra S, Daum R, Jones T, Bayer AS. 2010. Daptomycin-oxacillin combinations in treatment of experimental endocarditis caused by daptomycin-nonsusceptible strains of methicillin-resistant Staphylococcus aureus with evolving oxacillin susceptibility (the "seesaw effect"). Antimicrob Agents Chemother 54:3161-9.

254. Yang B, Lop ZX, Zhao YS, Ahmed S, Wang CQ, Zhang SS, Fu SL, Cao JY, Qiu YS. 2017. Combination Susceptibility Testing of Common Antimicrobials in Vitro and the Effects of Sub-MIC of Antimicrobials on Staphylococcus aureus Biofilm Formation. Frontiers in Microbiology 8.

255. Yousef RT, Tawil G, Abou-Shleib H. 1985. Combined action of amoxycillin and dicloxacillin against Staphylococcus aureus in vitro. Pharmazie 40:650-1.

256. You I, Kariyama R, Zervos MJ, Kumon H, Chow JW. 2000. In-vitro activity of arbekacin alone and in combination with vancomycin against gentamicin- and methicillin-resistant Staphylococcus aureus. Diagn Microbiol Infect Dis 36:37-41.

257. Yu XH, Song XJ, Cai Y, Liang BB, Lin DF, Wang R. 2010. In vitro activity of two old antibiotics against clinical isolates of methicillin-resistant Staphylococcus aureus. Journal of Antibiotics 63:657-659.

258. Yu Y, Huang HL, Ye XQ, Cai DT, Fang JT, Sun J, Liao XP, Liu YH. 2020. Synergistic Potential of Antimicrobial Combinations Against Methicillin-Resistant Staphylococcus aureus. Front Microbiol 11:1919.

259. Zakaria AS, Melake NA, Baky NA, El Rasheed NM, Ibrahim NH. 2012. In vitro and in vivo studies of antibacterial effect of ceftriaxone moxifloxacin combination against methicillin resistant Staphylococcus aureus biofilms formed on biomedical implants. African Journal of Microbiology Research 6:5399-5409.

260. Zarrouk V, Bozdogan B, Leclercq R, Garry L, Feger C, Carbon C, Fantin B. 2001. Activities of the combination of quinupristin-dalfopristin with rifampin in vitro and in experimental endocarditis due to Staphylococcus aureus strains with various phenotypes of resistance to macrolide-lincosamide-streptogramin antibiotics. Antimicrob Agents Chemother 45:1244-8.

261. Zhang R, Barreras Beltran IA, Ashford NK, Penewit K, Waalkes A, Holmes EA, Hines KM, Salipante SJ, Xu L, Werth BJ. 2021. Synergy Between Beta-Lactams and Lipo-, Glyco-, and Lipoglycopeptides, Is Independent of the Seesaw Effect in Methicillin-Resistant Staphylococcus aureus. Front Mol Biosci 8:688357.

262. Zhou YF, Xiong YQ, Tao MT, Li L, Bu MX, Sun J, Liao XP, Liu YH. 2018. Increased activity of linezolid in combination with rifampicin in a murine pneumonia model due to MRSA. J Antimicrob Chemother 73:1899-1907.

263. Zhou YF, Li L, Tao MT, Sun J, Liao XP, Liu YH, Xiong YQ. 2019. Linezolid and Rifampicin Combination to Combat cfr-Positive Multidrug-Resistant MRSA in Murine Models of Bacteremia and Skin and Skin Structure Infection. Front Microbiol 10:3080.

264. Zhou Y, Liu MJ, Liao XY, Chen YT, Liao QX, Lin JD, Lin HR, Huang YH. 2023. New Attempts to Inhibit Methicillin-Resistant Staphylococcus aureus Biofilm? A Combination of Daptomycin and Azithromycin. Infect Drug Resist 16:7029-7040.

265. Zinner SH, Lagast H, Klastersky J. 1981. Antistaphylococcal activity of rifampin with other antibiotics. J Infect Dis 144:365-71.
